# *Aureolib* — A Proteome Signature Library: Towards an Understanding of *Staphylococcus aureus* Pathophysiology

**DOI:** 10.1371/journal.pone.0070669

**Published:** 2013-08-13

**Authors:** Stephan Fuchs, Daniela Zühlke, Jan Pané-Farré, Harald Kusch, Carmen Wolf, Swantje Reiß, Le Thi Nguyen Binh, Dirk Albrecht, Katharina Riedel, Michael Hecker, Susanne Engelmann

**Affiliations:** Institut für Mikrobiologie, Ernst-Moritz-Arndt-Universität, Greifswald, Germany; University of Liverpool, United Kingdom

## Abstract

Gel-based proteomics is a powerful approach to study the physiology of *Staphylococcus aureus* under various growth restricting conditions. We analyzed 679 protein spots from a reference 2-dimensional gel of cytosolic proteins of *S. aureus* COL by mass spectrometry resulting in 521 different proteins. 4,692 time dependent protein synthesis profiles were generated by exposing *S. aureus* to nine infection-related stress and starvation stimuli (H_2_O_2_, diamide, paraquat, NO, fermentation, nitrate respiration, heat shock, puromycin, mupirocin). These expression profiles are stored in an online resource called *Aureolib* (http://www.aureolib.de). Moreover, information on target genes of 75 regulators and regulatory elements were included in the database. Cross-comparisons of this extensive data collection of protein synthesis profiles using the tools implemented in *Aureolib* lead to the identification of stress and starvation specific marker proteins. Altogether, 226 protein synthesis profiles showed induction ratios of 2.5-fold or higher under at least one of the tested conditions with 157 protein synthesis profiles specifically induced in response to a single stimulus. The respective proteins might serve as marker proteins for the corresponding stimulus. By contrast, proteins whose synthesis was increased or repressed in response to more than four stimuli are rather exceptional. The only protein that was induced by six stimuli is the universal stress protein SACOL1759. Most strikingly, cluster analyses of synthesis profiles of proteins differentially synthesized under at least one condition revealed only in rare cases a grouping that correlated with known regulon structures. The most prominent examples are the GapR, Rex, and CtsR regulon. In contrast, protein synthesis profiles of proteins belonging to the CodY and σ^B^ regulon are widely distributed. In summary, *Aureolib* is by far the most comprehensive protein expression database for *S. aureus* and provides an essential tool to decipher more complex adaptation processes in *S. aureus* during host pathogen interaction.

## Introduction

*Staphylococcus aureus* is an emerging pathogen and a leading cause of nosocomial infections worldwide [1]. It is responsible for a wide variety of infections ranging from mild skin diseases (furuncles, carbuncles) to life-threatening systemic infections such as bacteremia. The pathogenic diversity of *S. aureus* is mediated by a large set of virulence factors (for review see [2]) differently produced in different combinations in various isolates [3]. In addition, it becomes more and more evident that specific metabolic traits and their regulatory systems are crucial for fitness and survival of the pathogen during the infectious process and thus indirectly impact its virulence potential (for review see [4]). For instance, a nitric oxide inducible lactate dehydrogenase activity has been shown to be critical for *S. aureus* resistance against innate immunity [5].

For many years, gel-based proteomics has been extensively used to obtain meaningful insights into the adaptation of bacteria to various stressors and nutrient limitations. However, there are limitations to 2-dimensional (2D) protein gels that make only a part of the proteome accessible. Proteins with extreme p*I*s and molecular weights, very low abundant proteins and hydrophobic proteins cannot be detected using the 2D gel based approach. Hence, mass spectrometry (MS) based approaches relying on separation of complex protein or peptide mixtures by liquid chromatography or 1D SDS gel electrophoresis have been playing a pivotal role in proteomics during the last years (for review see [6]).

Despite covering only a subproteomic fraction gel based proteomics is still a powerful tool to address physiological issues (for review see [7]). For low complexity organisms like bacteria the majority of metabolic enzymes can be visualized by 2D polyacrylamide gel electrophoresis (PAGE) within the standard analytical window (pI 4–7; MW 10–150 kDa) [8], [9]. Different labeling techniques enable us to follow the entire life of the proteins from birth (synthesis) via adolescence (accumulation, modification) to death (damage, degradation). To date, gel based proteomics is the most sensitive technique to visualize newly synthesized proteins by radioactive pulse labeling. In order to study stress responses, protein synthesis patterns can be recorded before and immediately after exposure to changing environmental conditions without any interference from pre-accumulated proteins. Moreover, protein isoforms which differ in p*I* and molecular weight can be visualized and separately analyzed. For example, using gel based proteomics it has been shown that following hydrogen peroxide treatment, the glyceraldehyde-3-phosphate dehydrogenase is inactivated by thiol oxidation of a critical cysteine residue [10] and that the pyruvate-formate-lyase PflB is activated under anaerobic conditions by glycyl radicalization [11], [12]. In summary, gel-based proteomics remains an extremely valuable tool not only for (patho-) physiological studies [6], [13] but also for systems biology approaches [14] and antibiotic research [15].

Proteins whose synthesis is significantly changed under a defined condition compared to the control condition constitute the *proteomic signature* for the respective condition [16]. This characteristic set of proteins reflects the cell's state under a particular condition and they can be used as biomarkers. Meanwhile an impressive number of publications is available which describe the adaptation of *S. aureus* to various growth restricting conditions using a proteomic approach. Besides gel-based techniques, gel-free techniques are also being more frequently applied to identify proteins that might play a role during adaptation. In particular conditions have been examined which mimic the *in vivo* challenges *S. aureus* has to cope with during colonization or infection of the host such as oxidative stress [10], [17], [18], glucose starvation [8], [19], anaerobiosis [20], iron limitation [21], biofilm formation [22], and low temperatures [23] as well as exposure to antimicrobial agents [24], [25], [26], [27] and internalization by host cells [28]. A global comparison of these data sets is extremely difficult and to date an integrative approach to link expression data from different experiments has been missing.

In the present study, protein synthesis data of *S. aureus* COL obtained under nine different infection related conditions and covering 521 individual proteins and their isoforms (in total 679 protein spots) were inter-experimentally analyzed using an identification based approach. For instance, reactive oxygen and nitrogen species are effector molecules, which are involved in killing of bacteria after phagocytosis by neutrophils and macrophages. During the acute phase of an infection, invasion of neutrophils into infected tissues results in the reduction of the blood flow that in turn leads to oxygen concentrations below 1% [29]. Finally, *S. aureus* was exposed to several antibiotics including mupirocin which is used for elimination of staphylococci from nasal mucosa or skin. To process, screen, and analyze this huge amount of expression data and to visualize protein expression profiles a dedicated data management platform, *Aureolib*, was created. In this way, a more detailed view of general and specific gene expression programs of *S. aureus* under different growth-limiting conditions has been established.

## Results and Discussion

### Aureolib - a proteomic signature library for inter-experimental expression data analyses

Over the past years, we have intensively studied the response of *S. aureus* COL to various growth-limiting stimuli using a proteomics approach. The data generated up till now were more or less separately analyzed and subsequently compared by using lists of induced and/or repressed proteins [17], [18], [30]. This procedure, however, allows only a rather limited distinction between stress/starvation-specific and more general (*e.g.* growth rate dependent) effects and, hence, a more global approach for inter-experimental data analyses was urgently needed. In the present study, we used protein synthesis data of seven previously published stress experiments including oxidative stress (H_2_O_2_, diamide, paraquat), nitrosative stress (NO), oxygen limitation (fermentation, nitrate respiration), and sub-inhibitory concentrations of mupirocin (Table 1) [17], [18], [20], [24]. This data set was complemented by protein synthesis data in response to stimuli known to induce protein stress: heat shock and puromycin. For each experiment we used the same experimental design (Table S1) and applied growth restricting stimuli that led to a growth rate reduction of 50% or more during the early phase of stress (Fig. S1).

**Table 1:** Stress experiments included in the proteome signature library.

experiment	stressor/stimulus	medium^1^	reference
hydrogen peroxide	H_2_O_2_ [10 mM]	CDM	Wolf *et al*., 2008 [18]
diamide	diamide [1 mM]	CDM	Wolf *et al*., 2008 [18]
paraquat	paraquat [0.01 µM]	CDM	Wolf *et al*., 2008 [18]
nitric oxide	NO [500 µM]	CDM	Hochgräfe *et al*., 2008 [17]
fermentation	oxygen limitation	CDM	Fuchs *et al*., 2007 [20]
nitrate respiration	oxygen limitation	CDM + nitrate [8 mM]	Fuchs *et al*., 2007 [20]
heat	shift to 48°C	CDM	this study
puromycin	puromycin [3 µM]	CDM	this study
mupirocin	mupirocin [0.03 µM]	CDM	Reiß *et al*., 2012 [24]

1 CDM = chemically defined medium.

A comparison of the synthesis profiles of each protein spot under different stimuli was made possible by an integrative spot analysis which required the allocation of each protein spot detected under the different conditions to a corresponding spot on a 2D reference map (*master gel*). For establishing the reference map of cytoplasmic proteins of *S. aureus* COL, proteins were separated on 2D gels using a p*I* range of 4 to 7. Altogether, 679 protein spots were analysed by mass spectrometry (Fig. S2, for an interactive view see http://www.aureolib.de/?m1). Since some protein spots included more than one protein species we obtained 728 protein identifications corresponding to 521 protein species. This set of proteins corresponds to 41% of the cytoplasmic proteins of *S. aureus* COL predicted for this analytical proteomic window (Fig. S3, Table S2) and is involved in various cellular functions, such as biosynthetic and metabolic pathways (42%), gene expression and its regulation (13%), protein fate (6%), transport processes (2%) and several others (Fig. S4).

**Figure 1:**
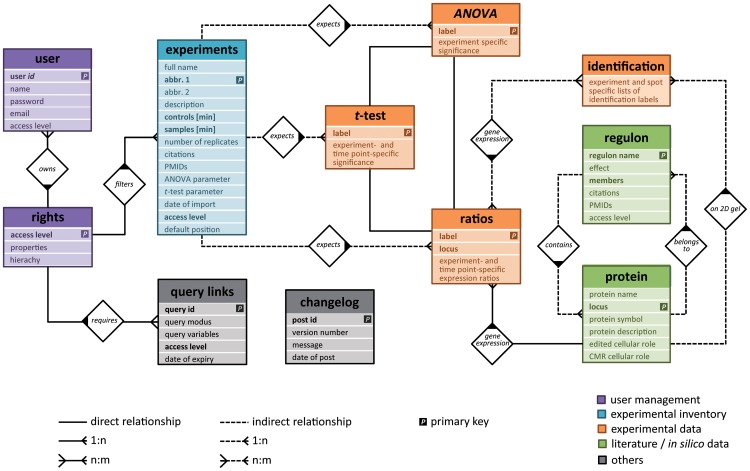
The relational database model. *Aureolib* uses a MySQL database to store expression data and relevant information on protein annotation, statistical analyses, and experimental setups.

**Figure 2:**
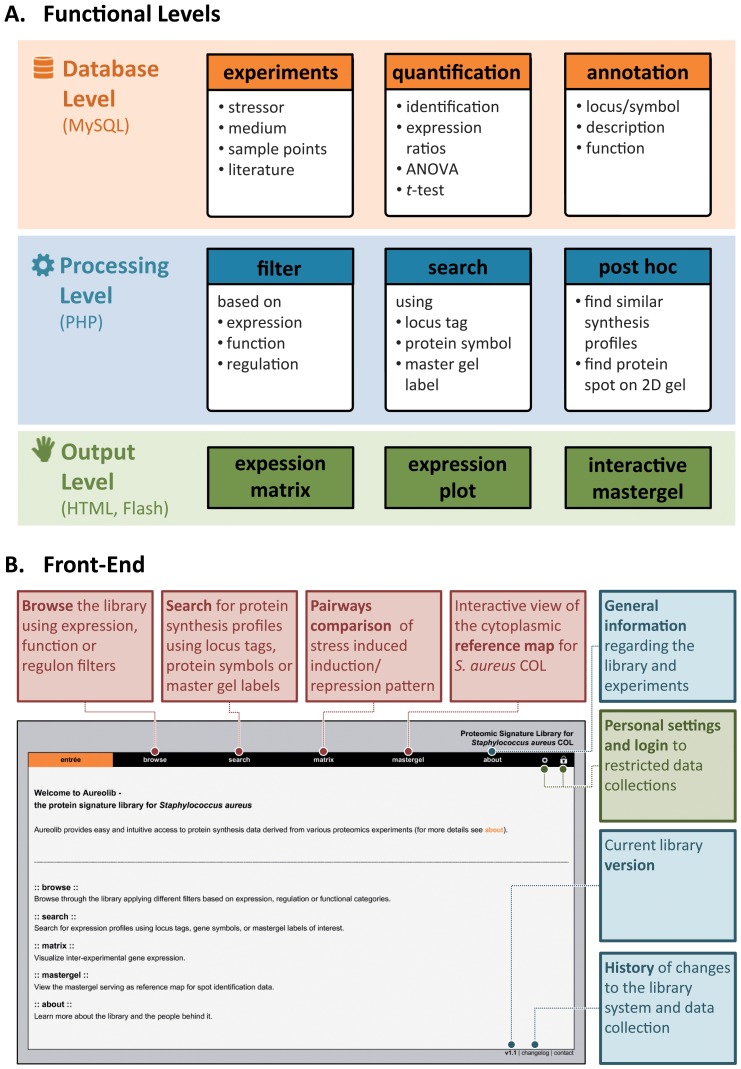
Functions and graphical user interface of *Aureolib*. (A) *Aureolib* consists of three different levels for data management, data processing and visualization of data. (**B**) Combined with an intuitive user interface accessible by generic web browsers *Aureolib* provides helpful tools for expression data analyses and data visualization.

An integrative database system named *Aureolib* based on a “PHP-backbone” communicating with a MySQL database was established to store expression data for *S. aureus* and other relevant information for inter-experimental analyses (Fig. 1 and 2A). The system is easy to handle, is extendable with other OMICS data if required, and allows a continuous implementation of expression profiles. The HTML-based user interface is accessible by generic web browsers (Fig. 2B; http://www.aureolib.de). Expression data can be visualized as inter-experimental protein expression profiles.

To select specific expression profiles, *Aureolib* provides complex search and filter options. There are three modes for data retrieval: (i) the browse mode, (ii) the search mode, and (iii) the matrix mode. The browse mode provides a filter-based presentation of synthesis profiles of proteins which belong to single or overlapping stimulons (*expression filters*). In addition, it is possible to search for expression profiles of proteins belonging to defined functional categories (*function filter*) such as glycolysis, TCC and fermentation pathways or to single regulons such as the Rex, SigB, CodY, CcpA, Fur, and PerR regulon (*regulation filter*). Using the search mode, synthesis profiles of selected proteins can be visualized.

Results obtained by the browse or search mode are shown in bar charts presenting synthesis data for all conditions stored in the library. Additionally, other relevant information such as statistical significance and protein annotation is provided. Using the interactive master gel viewer, protein spots can be localized on the master gel and in this way, the most abundant spot(s) for proteins present as multiple spots can be identified. Finally, the matrix mode can be used to perform a pair wise comparison of expression profiles of two experiments so that overlapping marker proteins can be detected for the respective stimuli.

### General aspects of studying adaptation to stress and starvation in *S. aureus*

**Figure 3:**
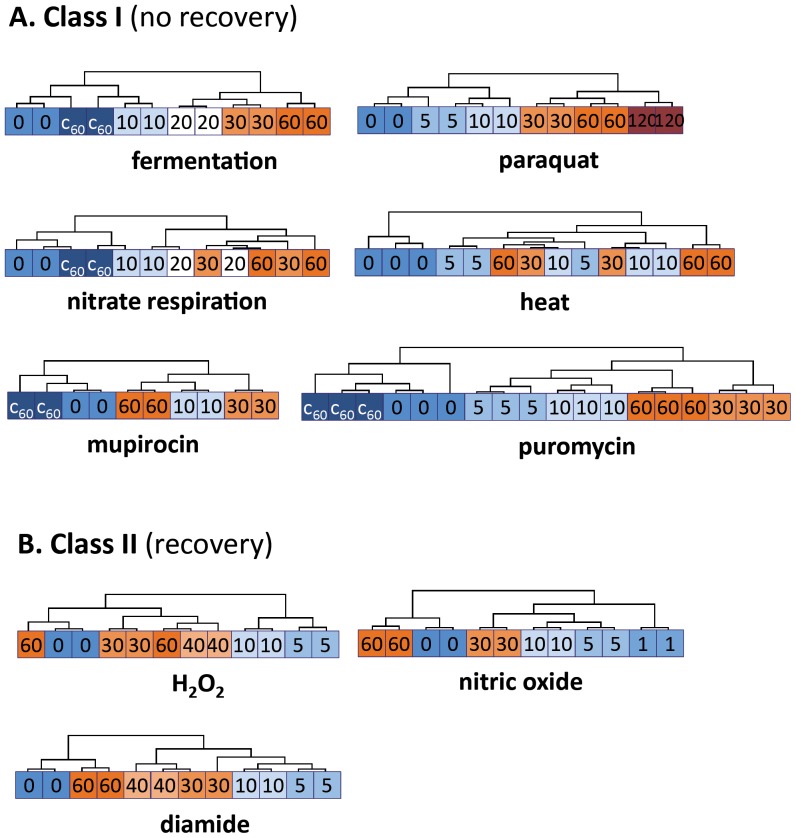
Intra-experimental comparison of protein synthesis patterns in response to different stimuli. Quantification data for all detected protein spots were normalized (total) and standardized (z-score). Significant changes of spot intensities were determined for each experiment (ANOVA, α = 0.1, distribution based on 1000 permutations) and the corresponding expression values were used for hierarchical sample clustering (HCL, Euclidean distance, complete linkage). Accordingly, the stimuli can be divided into two classes: (**A**) Stimuli causing continuous changes of the protein synthesis pattern and (**B**) stimuli transiently affecting the protein synthesis pattern.

Adaptation to the nine different stimuli was characterized by dramatic changes of the protein synthesis pattern immediately after exposure to stress. Hierarchical sample clustering based on differentially expressed proteins revealed two classes of stimuli (Fig. 3). Exposure to class I stimuli (H_2_O_2_, diamide, nitric oxide) was characterized by transient changes of the global protein synthesis pattern, while class II stimuli (heat, oxygen limitation, paraquat, puromycin, mupirocin) caused a prolonged reorganization of cytoplasmic protein synthesis. Presumably, those stimuli that belong to the first group are efficiently eliminated, for example by degradation, which allows restoration of the growth rate after adaptation (Fig. S1). This was particularly evident for nitric oxide and hydrogen peroxide stress: Immediately after exposure to these stimuli, cells stopped growth and the protein synthesis pattern was dramatically changed. Sixty minutes later, the growth rate recovered to the pre-stressed level and the protein synthesis pattern shifted almost completely back to that of the unstressed state (Fig. S1 and Fig. 3).

Each stressor induced and repressed the synthesis of a typical set of protein spots which is defined as its proteome signature [16]. A comparison of protein synthesis patterns before and after exposure to the respective stimuli allows a rapid assignment of proteins to stimulons. These proteins might be involved in adaptation to the respective stimulus. Compared to previous publications from our group [17], [18], [20] the number of identified protein spots induced and repressed by these stimuli has been significantly increased because this present study benefits from the more sensitive protein identification techniques applied to the 2D reference gel. This study shows that the synthesis of every protein was affected by at least one of the stressors. Of importance, we note that GyrB, whose transcript level is widely used as a reference, seems to be differently synthesized in response to hydrogen peroxide, nitric oxide, and mupirocin conditions. In future, more attention should be focused on the selection of genes used as references for transcriptomic data, and *Aureolib* may assist with this process. Altogether, 226 protein synthesis profiles showed induction ratios of 2.5-fold or higher under at least one of the tested conditions. For instance, 73 protein synthesis profiles show an induction during diamide stress followed by hydrogen peroxide (n = 48), nitrogen oxide stress (n = 47) and fermentation (n = 44).

**Figure 4:**
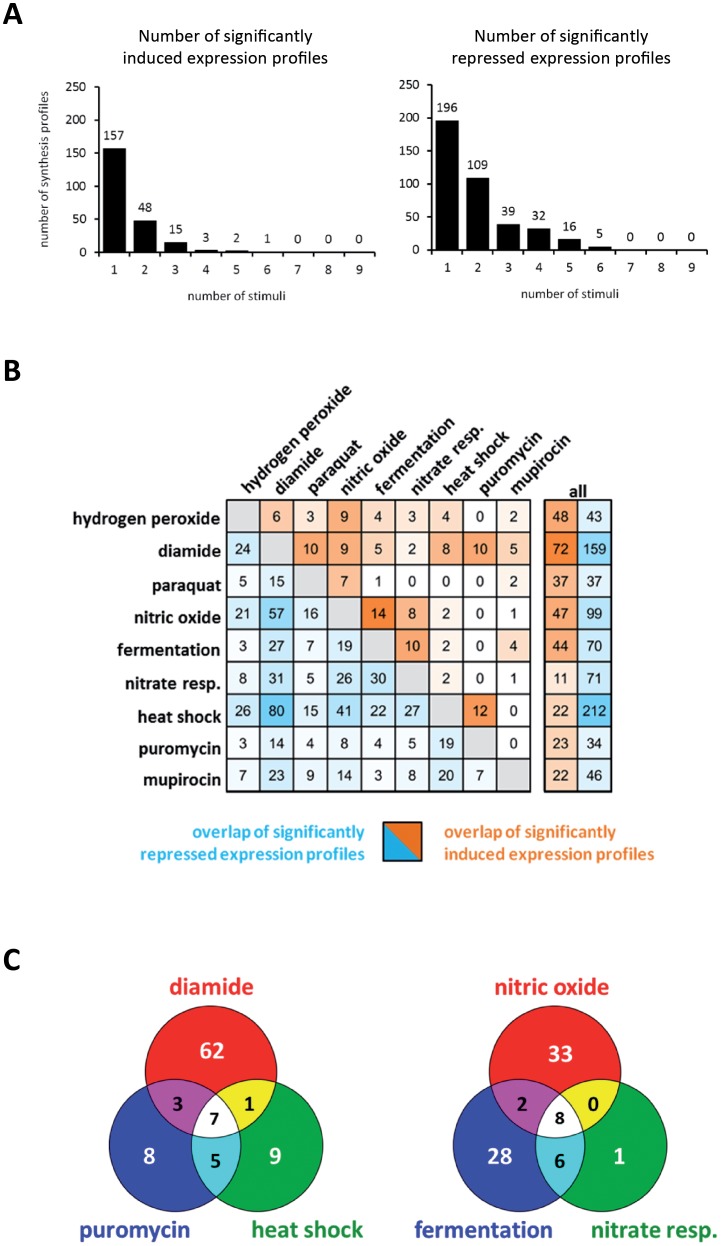
General aspects of *S. aureus* stress response. Inter-experimental analyses of stress induced protein synthesis patterns revealed general and specific effects on global protein synthesis. (**A**) The majority of the synthesis profiles showed a significant (ANOVA, α = 0.1, p-values based on 1000 permutations, absolute threshold of 2.5) induction or repression rate in response to one or two stimuli. (**B**) To identify more general effects a pairwise comparison of all experiments was performed using protein synthesis profiles significantly changed at least one time point following exposure to the different stimuli (t-test, α = 0.1; p-values based on all possible permutations, adjusted Bonferroni correction). The number of induced (orange) and repressed (blue) protein synthesis profiles shared by the respective stimuli is shown. The total number of synthesis profiles significantly induced or repressed by the respective stimulus are given on the right side. (**C**) Overlap of marker proteins induced in response to diamide, puromycin, and heat as well as to nitric oxide, fermentation, and nitrate respiration are shown.

**Figure 5:**
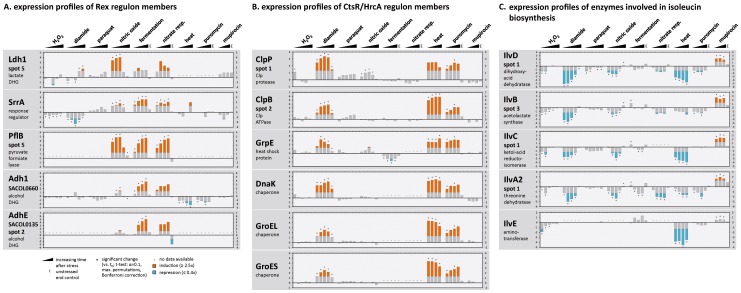
Inter-experimental expression profiles of selected proteins. Synthesis ratios (stress *vs.* control) are shown as log_2_ values. Fold changes above 2.5 and below 0.4 are colored in orange and blue, respectively. Significant changes are marked by an asterisk (*). Synthesis profiles of proteins belonging to the Rex (**A**) and Ctsr/HrcA (**B**) regulon as well as proteins involved in leucine biosynthesis (**C**) are shown in more detail.

By comparing different proteome signatures, proteins with specifically changed synthesis profiles can be clearly distinguished from proteins whose synthesis was rather generally induced or repressed irrespective of the stimulus. Specific effects on protein synthesis can be ascribed to a defined signal which is associated with only a limited number of closely related stimuli, while non-specific effects that are independent of the specific stimulus such as the *stringent response* are generally observed under growth restricting conditions. The synthesis of the majority of the proteins was changed by one or two of the stimuli (Fig. 4A). Altogether, 157 protein synthesis profiles have been shown to be induced at least 2.5-fold in response to a single stimulus and an additional 195 were repressed. The corresponding proteins might serve as marker proteins for the respective stimulus. For instance, positive effects caused by mupirocin are highly specific (Fig. 4B; http://www.aureolib.de/?m2). This includes the strong induction of enzymes involved in isoleucine biosynthesis (IlvD, IlvB, IlvC, IlvA2) and isoleucyl-tRNA charging (IleS). These enzymes are clearly repressed by other growth-limiting stimuli tested in this study (Fig. 5C). An induction of these proteins thus specifically indicates branched chain amino acid starvation.

By contrast, proteins whose synthesis was increased or repressed in response to more than four stimuli are rather exceptional. Only one protein, the universal stress protein SACOL1759, was induced by six stimuli and a very heterogeneous group of five proteins including aconitase (AcnA), dihydroxy-acid dehydratase (IlvA), IMP cyclohydrolase (PurH), carbamoyl-phosphate synthase (CarB), and SACOL0430 was repressed in response to six stimuli.

**Figure 6:**
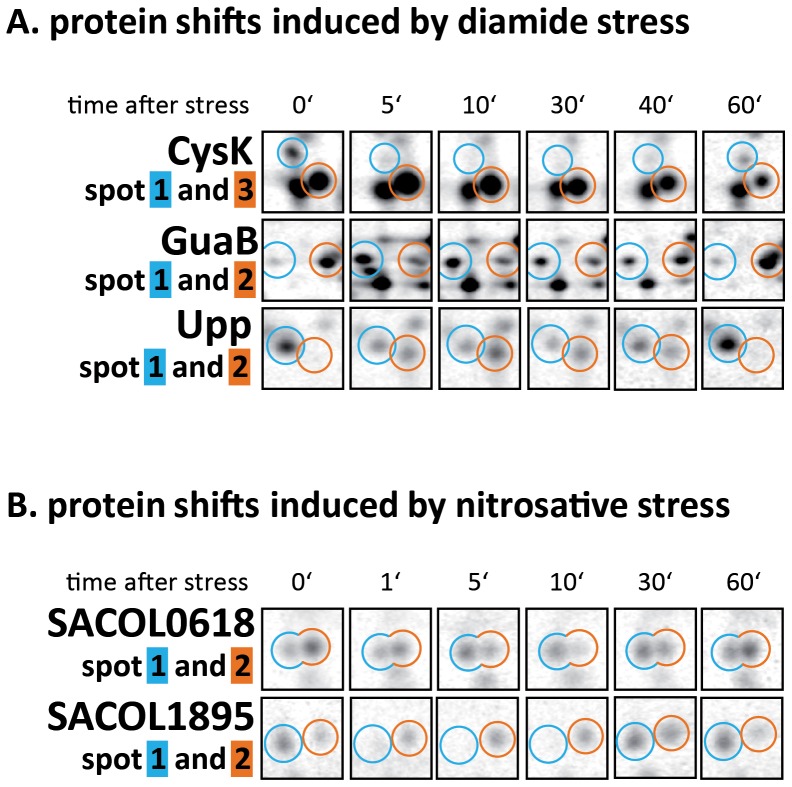
Differential expression of protein isoforms in response to diamide (A) and nitrosative stress (B). Sectors of 2D gels covering the regions where CysK, GuaB, Upp, SACOL0618 and SACOL1895 are located. Proteins represented by multiple spots indicate post translational modifications. In the present approach the synthesis of protein isoforms differing in p*I* and/or molecular weight was separately analyzed.

Interestingly, 133 proteins are represented by multiple spots on the gel indicating post translational modifications (Fig. S3). In the present approach, expression data of protein isoforms were separately analyzed resulting in more than one synthesis profile for the respective proteins. In this way, we were able to ascertain whether the same stimulus can differentially affect isoforms of a certain protein. For instance exposure to diamide and NO stress differently affected the expression profiles of the isoforms of CysK, GuaB, and Upp and of SACOL0618 and SACOL1895 (Fig. 6). As a result one protein isoform clearly increased whereas the synthesis rate of the second isoform was diminished. Similar observations have been described previously for Gap, AhpC and MvaS in response to hydrogen peroxide treatment induced by thiol oxidation of the cysteinyl residue [10]. To understand the cell physiology consequences associated with isoform changes, post-translational modifications of these particular proteins will be investigated in future studies.

### Integration of stimulons into regulatory networks to elucidate signal transduction under stress and starvation conditions

Highly sophisticated regulatory networks are involved in a multitude of specific and general adaptation and defense mechanisms. A comprehensive gene expression data analysis under defined growth restricting conditions may help to unravel these regulatory networks.

We uploaded available information on 75 known regulons of *S. aureus* [31], [32], [33] (http://regprecise.lbl.gov) into the database. For 43 regulons gene products of at least one regulon member were identified on the master gel. Accordingly, 40% of the identified proteins are regulated by at least one of these regulators. For instance, 48 proteins belong to the CodY regulon, 41 proteins to the SigB regulon, 18 proteins to the CcpA regulon, eight proteins to the CtsR regulon (including five proteins additionally regulated by HrcA), and eight proteins to the Rex regulon (Table 2). The expression of 39 proteins has been shown to be influenced by more than one of the known regulators. Comparing the protein synthesis profiles using hierarchical clustering, proteins of only a few regulons clustered closely together. These are members of the GapR, Rex and CtsR regulon. In contrast, protein synthesis profiles of proteins belonging to the CodY and σ^B^ regulon are widely distributed (Fig. S5). These results suggest that additional regulators and/or post transcriptional events play a more significant role in gene expression in *S. aureus*.

**Table 2:** Regulons in *S. aureus*.

regulator or regulatory element	total^1^	H_2_O_2_		diamide		paraquat		NO		ferm		NO_3_ resp.		heat		puromycin		mupirocin	
		up	down	up	down	up	down	up	down	up	down	up	down	up	down	up	down	up	down
ArgR^a^	2	0	0	0	1	0	0	0	0	0	0	0	0	0	0	0	0	0	0
CcpA^a^	18	2	1	1	8	1	2	2	5	3	3	2	5	1	10	0	1	0	3
CodY^a^	49	2	14	5	13	4	5	3	11	2	4	0	6	2	26	1	7	5	4
CtsR^a^	8	0	0	7	0	1	0	0	1	0	1	0	1	7	0	7	0	0	0
CymR^a^	4	1	0	3	1	0	0	0	0	0	0	0	0	0	2	0	0	0	0
FapR^a^	6	1	0	0	2	0	1	0	0	0	1	0	2	0	3	0	0	0	1
FMN^a^	2	1	0	1	0	0	0	0	0	0	0	0	0	1	0	0	0	0	0
Fur^a^	3	2	0	0	1	0	0	0	2	0	1	0	2	0	1	1	0	0	1
GapR^a^	6	2	0	1	3	0	1	4	0	6	1	5	0	1	2	0	1	0	1
GlmS^a^	1	0	0	0	1	0	0	0	0	1	0	1	0	0	0	0	0	0	0
GlnR^a^	1	0	1	0	1	0	0	0	1	0	0	0	0	0	1	0	0	0	0
GltC^a^	1	0	0	0	1	0	0	0	0	0	0	0	0	0	0	0	0	0	0
Glycine^a^	1	0	0	0	0	0	0	0	0	0	0	0	0	0	1	0	0	0	0
HrcA^a^	5	0	0	4	0	1	0	0	0	0	1	0	0	4	0	4	0	0	0
L10_leader^a^	2	0	0	0	2	0	0	0	1	0	1	0	1	0	0	0	0	0	0
LexA^a^	6	1	0	0	1	0	0	0	0	1	1	0	0	0	2	0	0	1	0
Lysine^a^	7	0	2	1	1	1	0	0	0	0	0	0	1	0	5	0	0	0	0
MtlR^a^	2	0	0	0	1	0	0	0	0	0	0	0	0	0	1	0	0	0	0
NrdR^a^	2	1	2	0	2	0	0	0	2	0	0	0	0	0	2	0	1	0	0
PdxR^a^	1	0	0	0	0	0	0	0	0	0	1	0	0	0	0	0	0	0	0
PerR^a^	15	3	0	3	5	2	1	1	4	1	3	0	3	0	6	1	1	1	0
Purine^a^	3	0	1	1	2	2	0	0	1	0	1	0	0	0	1	0	0	0	0
PurR^a^	12	2	1	2	6	0	2	0	4	1	2	0	4	0	7	0	1	0	6
PyrR^a^	6	5	0	0	2	0	4	0	1	0	3	0	3	0	2	0	3	0	2
Rex^b^	6	0	0	1	2	0	1	5	1	4	0	2	0	0	2	0	1	0	0
SaeR^a^	1	0	0	0	0	0	0	0	0	0	0	0	0	0	0	0	0	0	0
SAM^a^	2	0	0	1	1	0	0	0	1	0	0	0	0	0	0	0	0	0	0
ScrR^a^	2	0	0	0	0	0	0	0	0	0	0	0	0	0	0	0	0	0	0
SigB−c	4	3	0	0	3	0	0	0	3	0	0	0	0	0	2	0	1	0	1
SigB+^c^	40	3	3	3	15	0	3	3	8	2	3	0	2	0	15	0	3	1	5
T-box(Ala)^a^	1	0	0	0	0	0	0	0	0	0	0	0	0	0	1	0	0	0	0
T-box(Asn)^a^	1	0	0	0	1	0	0	0	1	0	1	0	1	0	1	0	0	0	1
T-box(Cys)^a^	1	0	0	0	0	0	0	0	0	0	0	0	0	0	0	0	0	0	0
T-box(Gly)^a^	1	0	1	0	1	0	0	0	1	0	0	0	1	0	1	0	0	0	0
T-box(Ile)^a^	1	0	1	0	1	0	0	0	1	1	1	0	1	0	1	0	0	1	0
T-box(Leu)^a^	1	0	0	0	1	0	0	0	0	0	0	0	0	0	1	0	0	0	0
T-box(Met)^a^	5	1	2	0	1	0	3	0	2	0	0	0	0	2	2	1	0	0	4
T-box(Phe)^a^	2	0	1	0	0	0	0	0	2	0	0	0	0	0	1	0	0	0	0
T-box(Ser)^a^	1	0	0	0	0	0	0	0	0	0	0	0	0	0	1	0	0	0	0
T-box(Thr)^a^	1	0	0	0	1	0	0	0	1	0	0	0	0	0	1	0	0	0	0
T-box(Tyr)^a^	1	0	1	0	1	0	0	1	0	0	0	0	0	0	1	0	0	0	1
T-box(Val)^a^	1	0	0	0	0	0	0	0	1	0	0	0	0	0	0	0	0	0	0
Zur^a^	1	0	0	0	0	0	0	0	0	0	1	0	0	0	0	0	0	0	0

Regulators or regulatory elements and the number of assigned proteins which are significantly and at least 2.5-fold induced (up) or repressed (down) on synthesis level.

1 number of corresponding proteins identified on the master gel belonging to the respective regulon.

a source: http://regprecise.lbl.gov [32].

b source: Pagels *et al.* (2010) [36].

c source: Bischoff et al. 2004 [48], Pané-Farré et al. 2006 [49], Ziebandt et al. 2001 [71], Ziebandt et al. 2004 [72].

Different stress conditions sharing a defined set of marker proteins might activate the same regulatory networks. Hence, it seems very likely that these stimuli are sensed and transduced by the same pathways. Using *Aureolib* a pairwise comparison of all tested conditions was performed to reveal such common effects on protein synthesis (Fig. 4B). Two groups of stimuli attracted our special attention: (i) fermentation, nitrate respiration, and nitric oxide and (ii) heat, diamide, and puromycin. The first group of stimuli shares eight induced proteins which belong to the Rex regulon and the second group induces seven marker proteins each of which is regulated by CtsR (Fig. 4C).

On the other hand there are proteome signatures that are very distinct. For instance mupirocin and puromycin have only a few repressed and none of the induced marker proteins in common (Fig. 4B). Although both antibiotics terminate translation, the physiological consequences for the cell are obviously different. While mupirocin increases the pool of uncharged tRNAs which is related to the stringent response, puromycin induces damaged proteins by binding to the nascent peptide chain [34], [35]. This example highlights the ability of proteomic signatures to discriminate the mode of action of antibiotic substances even if they act on the same process in the cell but at different steps.

#### The Rex modulon is specifically induced by stimuli associated with reduced respiratory activity

The redox sensitive regulator Rex has been considered as a central regulator for anaerobic gene expression and an essential component for NO stress resistance in *S. aureus* [5], [36]. Altogether, 66 proteins have been postulated whose synthesis might be directly affected by Rex [36]. Among them are 36 proteins that are predicted to fall within the analytical window of our gel based approach and 20 of these were expressed at detectable amounts. De-repression of Rex controlled proteins might be excellent marker proteins for an increasing NADH pool. As expected, expression profiles of these proteins are characterized by an induction in response to oxygen limitation (fermentation and nitrate respiration) and to NO stress [17], [20], [37], but remained unaffected after exposure to the other stimuli examined in this study (Fig. 5A).

Interestingly, the flavohemoprotein Hmp and several glycolytic enzymes show a similar expression pattern as found for Rex regulated proteins. Recently we showed that although a Rex binding site has been found 363 bp upstream of *hmp*, the expression of the gene was not de-repressed by the inactivation of *rex* [36]. Consequently, at least one additional regulatory mechanism might exist that mediates Hmp induction independently of Rex and hence regulators involved in *hmp* expression will be of special interest to gain better understanding of signaling and signal transduction under these conditions.

#### Protein stress induced the CtsR/HrcA regulon

Heat shock, diamide, and puromycin share a group of seven induced proteins (Fig. 4C). This cluster contains the most prominent heat shock proteins ClpP, ClpB, ClpC, DnaK, GroEL, GroES, and GrpE [38], [39]. The respective expression profiles are remarkably similar and show a maximal induction after heat stress (Fig. 5B; http://www.aureolib.de/?m3).The synthesis of these proteins is regulated by CtsR known as a global heat shock repressor in low GC, Gram-positive bacteria [40]. The CtsR encoding gene is autoregulated and forms a tetracistronic operon together with *mcsA*, *mcsB*, and *clpC*. The synthesis profile of CtsR is similar to that of the heat shock proteins with a maximal induction ratio in response to heat stress (40-fold) and clearly reduced induction ratios after the addition of diamide (up to 18-fold) and puromycin (up to 5-fold). Based on the expression profiles of CtsR-dependent proteins we suggest that CtsR is inactivated most efficiently by heat stress in *S. aureus*.

In *Bacillus subtilis*, a dual activity control of CtsR activity has been described. Under heat stress, CtsR acts as an intrinsic thermosensor and is degraded by a two-step mechanism [41], [42] while during oxidative stress CtsR activity is subjected to a thiol-dependent regulatory pathway [41]. The induction profiles of CtsR and CtsR regulated proteins in response to heat and diamide imply a similar dual control of CtsR activity in *S. aureus*. Interestingly, both stressors share the highest number of proteins with reduced synthesis rates (Fig. 4B).

Puromycin led to the weakest induction of CtsR and its regulon. The aminonucleoside antibiotic resembles the 3′-end (CCA) of aminoacyl-tRNAs and binds to the A-site of the ribosome [35]. The subsequent incorporation of puromycin into the nascent polypeptide chain causes abortion of translation and the release of the peptide from the ribosome. As a consequence, misfolded and non-functional peptides accumulate in the cell.

#### The stringent response

The stringent response is characterized by a massive down-regulation of the translational machinery and other energy consuming processes to prevent waste of nutrients during starvation and other growth restricting conditions (for review see [43]) and it is triggered by an accumulation of uncharged tRNAs. Mupirocin is an antagonist of the isoleucyl-tRNA synthetase [34] and it has been demonstrated that the synthesis of 20 proteins was induced while the synthesis of 60 proteins was significantly repressed in response to mupirocin [24]. Since *stringent control* represents a crucial adaptive strategy for survival of bacteria under growth restricting conditions, the synthesis of these proteins may also be affected by other growth limiting conditions. In fact, 53 of the repressed protein spots were also found to be repressed under at least one additional stress condition. The most prominent functional categories were nucleotide biosynthesis, energy metabolism and translation. Remarkably, signal intensities of seven protein spots were reduced following treatment with five additional stress stimuli. These are AcnA spot 1, CarB, PurH spot 1 and spot 3, PurQ, PyrF and SACOL0430 spot 1 which are mainly involved in nucleotide biosynthesis. A pair wise comparison of all tested conditions revealed the most significant overlap of repressed marker proteins to heat shock (28 of 212 protein spots repressed by heat) and diamide stress (24 of 158 protein spots repressed by diamide) followed by NO (19 of 99 protein spots repressed by NO) and paraquat stress (14 of 37 protein spots repressed by paraquat) (Fig. 4B).

#### The global repressor CodY

CodY is a global transcriptional repressor in Gram-positive bacteria [44], [45], which shapes the expression of a multitude of genes involved in adaptation to stationary phase. Altogether 48 gene products of CodY target genes in *S. aureus* were identified in the present approach (Table 2). Expression profiles of these proteins are highly heterogeneous (http://www.aureolib.de/?m12). CodY synthesis itself was repressed in response to mupirocin as well as under heat shock and slightly after exposition to diamide and hydrogen peroxide. However, with the exception of mupirocin, repression of CodY is not correlated with an increased synthesis of proteins under negative control of CodY. On the contrary, under these conditions proteins repressed by CodY [46], [47] (http://regprecise.lbl.gov) show even stronger (at least 2.5 fold) and significant repression (13 proteins by diamide, 26 proteins by heat stress and 17 proteins by hydrogen peroxide) and only a small number was found with significantly increased (at least 2.5 fold) translational activity (seven protein spots by diamide, and two protein spots by both heat and hydrogen peroxide). The synthesis of twelve proteins belonging to the CodY regulon was significantly and at least 2.5-fold repressed by more than one of these conditions. These are mainly proteins involved in amino acid biosynthesis while the synthesis of the other proteins was more specifically affected by one of these stressors.

Most strikingly, the expression of 21 CodY dependent proteins has been described to be affected by one or two further regulators which might explain the varying synthesis profiles of these proteins (Fig. S6).

#### In contrast to *B. subtilis*, the σ^B^ regulon in *S. aureus* is not generally activated in response to stress

**Figure 7:**
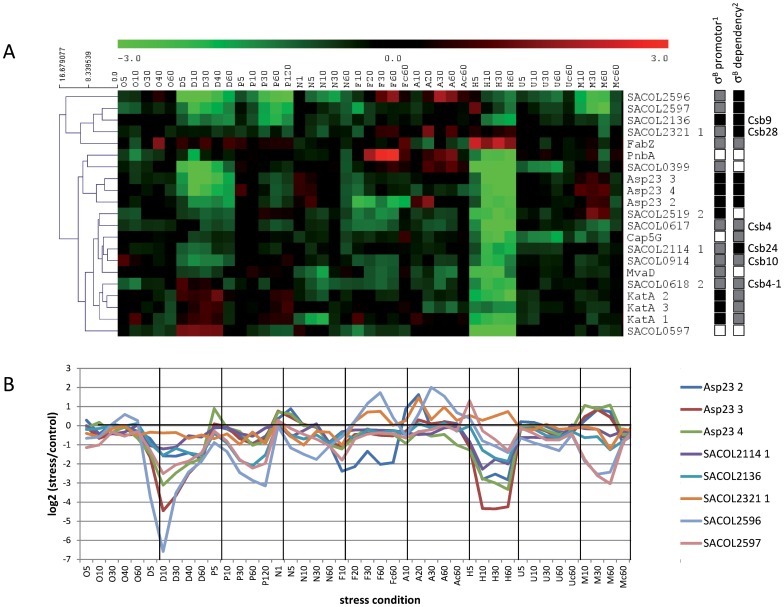
Expression clusters of σ^B^-dependent proteins. (**A**) Hierarchical clustering (Euclidian distance/complete linkage) of synthesis profiles of proteins for which transcription of the respective genes has been shown to be influenced by σ^B^ [48], [49], [50]. (**B**) Expression profiles of solely σ^B^-dependent proteins. Abbreviations for stress conditions are: O (H_2_O_2_), D (diamide), P (paraquat), N (nitrogen monoxide), F (fermentation), A (nitrate respiration), H (heat stress), U (puromycin), and M (mupirocin). ^1)^ Support for a σ^B^-dependent transcriptional start point: experimental (black square), predicted (gray square), not identified yet (white square) [37], [43], [70]. ^2)^ Impact of σ^B^: solely σ^B^-dependent/major effect on expression (black square), co-regulated by additional factors (gray squares), or not determined (white square) [50], [70]. Csb (controlled by sigma B) nomenclature based on Gertz *et al.* [50].

In *S. aureus* an influence of σ^B^ on transcription has been reported for about 230 genes [48], [49], [50]. For 40 of these genes at least one corresponding protein spot was identified on our protein reference map (Table 2). Since proteins can occur on 2D gels as multiple spots due to protein modification, a total number of 63 protein spots was assigned to the σ^B^-regulon. For 21 protein spots, representing 17 individual proteins, complete synthesis profiles were available for all tested stress conditions. In order to analyze whether distinct classes of σ^B^-regulated proteins can be identified, the 21 synthesis profiles were grouped using hierarchical clustering. Surprisingly, the expression patterns for these 21 protein spots were rather heterogeneous (Fig. 7A). Furthermore, if the presence of a σ^B^-promoter or the degree of σ^B^-dependency (complete dependency versus partial dependency) was taken into account, no clear correlation between the clustering pattern and the extent of σ^B^ contribution to expression was evident. In this context it is important to note that compared to growth in complex media (*e.g.* LB-medium), the basal level of σ^B^-activity in cells grown in the synthetic medium used for the radioactive labeling experiments is very high and may thus obscure further up-regulation of σ^B^ in response to stress. However, expression of the majority of the proteins with a strong σ^B^-dependency (Asp23, SACOL2114/Csb24, SACOL2136/Csb9 SACOL2321/Csb28, SACOL2596, SACOL2597) was actually down-regulated under several of the stress conditions investigated in this study (Fig. 7B). Together these results do not provide clear support for the function of σ^B^ as regulator of a general stress response in *S. aureus*. In fact, these observations indicate that the composition of the σ^B^-regulon, the regulation of σ^B^-activity and thus the function of the σ^B^-regulon, has been adapted to match the specific needs of *S. aureus*.

### New insights into the response of *S. aureus* to damage induced by hydrogen peroxide

Oxidative stress represents one of the challenges *S. aureus* has to cope with under *in vivo* conditions. During infection, bacteria are ingested by phagocytic cells such as neutrophils and macrophages and thus exposed to the oxidative burst, a microbicidal system using reactive oxygen and nitrogen species which are mainly generated by the phagocyte NADPH oxidase and inducible nitric oxide synthase, respectively. In order to understand host pathogen interactions more comprehensively the oxidative stress response of *S. aureus* has been for us a matter of special attention for several years [10], [18].

Using the 2D reference map established in the present study, the number of identified protein spots whose synthesis was at least 2-fold induced by hydrogen peroxide treatment has been extended from 28 to 97 [18] (http://www.aureolib.de/?m4). Among them are 20 proteins whose synthesis was specifically and at least 2.5 fold induced by this stimulus and can therefore be viewed as marker proteins (http://www.aureolib.de/?m5). The synthesis of the remaining proteins was induced by at least one additional stimulus. The strongest overlap was found with the nitric oxide stress stimulon (9 proteins) and the diamide stress stimulon (6 proteins).

**Figure 8:**
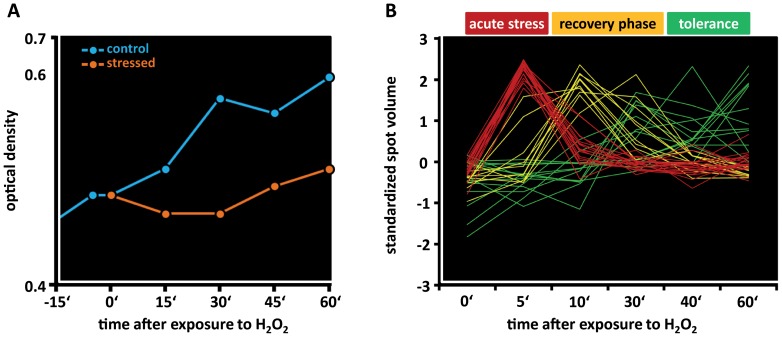
Response of *S. aureus* to H_2_O_2_. (**A**) *S. aureus* COL was cultivated in synthetic medium at 37°C and treated with 10 mM H_2_O_2_ at OD_500_ 0.5 (0 min). (**B**) Protein synthesis profiles were normalized using total normalization and standardized (z-score). Significantly changed synthesis profiles were determined by ANOVA (α = 0.1, distribution based on all permutations, absolute threshold of 2.5). Based on their expression kinetics in response to H_2_O_2_, proteins can be allocated to three groups: proteins which are induced during the acute phase immediately after imposition of stress (red lines), proteins induced during resumption of growth (blue lines) and proteins highly expressed in the tolerance phase (green lines).

Analyzing the expression kinetics of H_2_O_2_ induced proteins, the present analysis revealed that adaptation of *S. aureus* to hydrogen peroxide stress proceeds in at least three different phases: (i) acute phase, (ii) recovery phase, and (iii) tolerance phase (Fig. 8, Fig. S7). The acute phase is characterized by an immediate growth arrest accompanied by a strong induction of RecF (SACOL0004), GyrB and of enzymes involved in energy metabolism. Since RecB, RecC, and RecD are missing in *S. aureus*, the RecF system provides probably the major repair system for single strand DNA gaps and double strand DNA breaks [51]. Recently, the initial steps of DNA repair were elucidated for the Gram-negative model organism *E. coli* [52]. Briefly, single strand DNA is primarily bound by Ssb and the helicase RecQ. RecQ stimulates the exonuclease activity of recruited RecJ which expands the DNA gap to a single strand region. A protein complex consisting of RecF, RecO, and RecR binds to this region and recruits RecA which in turn mediates homologous recombination and repair of DNA. The immediate induction of SACOL0917 which contains a NifU domain known to bind iron [53] showed that this protein might also be involved in adaptation to H_2_O_2_ induced oxidative stress.

The recovery phase is characterized by resumption of growth and down-regulation of RecF synthesis. At the same time synthesis of the exconuclease UvrABC and RecA was induced up to 8-fold. The substrate of UvrABC is the dsDNA heteroduplex molecule resulting from RecA mediated pairing of the ssDNA with its undamaged homologous sequences [54]. This might be the main repair mechanism of DNA damage that arrests replication. The increased synthesis of proteins involved in purine and pyrimidine ribonucleotide biosynthesis (DeoB, CarA, CarB, PurA, PurK, PyrF, PyrC, PyrB) indicates an increased demand for nucleotides in response to oxidative stress. Interestingly, SACOL1985 and the GTP-binding protein TypA show a very specific induction during this phase of the oxidative stress response.

The tolerance phase is characterized by a complete recovery of growth and the induction of ten proteins. Among them is the alternative sigma factor B (RpoF; σ^B^) (up to 23-fold). The synthesis of further σ^B^ dependent proteins was not obviously affected under these conditions (Fig. 7A).

Strikingly, with the exception PerR and CymR, respectively, none of the known regulators might be clearly involved in the expression of the early phase proteins. For the synthesis of proteins of the recovery phase, there is first evidence that LexA, PyrR, PurR and GapR (CggR) inactivation might be important. Moreover, repression of a multitude of CodY dependent proteins has been observed in response to hydrogen peroxide stress (Fig. S7).

Interestingly, expression of most of the classical oxidative stress proteins involved in detoxification of oxygen radicals (KatA, SodA1, and SodA2) or in protection from damage caused by these radicals (TrxB) belonging to the PerR regulon was not significantly induced by H_2_O_2_ (http://www.aureolib.de/?m6). Considering the increased resistance of growing *S. aureus* cells to hydrogen peroxide it has been previously suggested that detoxifying enzymes might be present in significantly higher amounts than in sensitive bacteria such as *B. subtilis* [18]. Indeed, based on the absolute protein amounts for cytoplasmic proteins [55], the cellular concentration of KatA in exponentially growing cells was 2.9 fold higher in *S. aureus* than in *B. subtilis*. Similar observations were made for superoxide dismutases: while in *B. subtilis* for SodA 31,941 copies/µm^3^ were found, the copy number for SodA2 in *S. aureus* was significantly higher (73,779 copies/µm^3^).

Besides proteins of known functions, several so far completely uncharacterized proteins were induced by H_2_O_2_. Based on their expression kinetics they are associated either with the acute stress phase (SACOL1386, SACOL1447, SACOL2106, SACOL2722, SACOL0618, SACOL0917, SACOL1445; http://www.aureolib.de/?m7) or with the tolerance phase (SACOL0427, SACOL0457, SACOL1483, SACOL1768, SACOL2171; http://www.aureolib.de/?m8).

### Stress-specific induction patterns of the synthesis of hypothetical proteins provide first hints for the function of these proteins

Of the predicted cytoplasmic proteins in *S. aureus* COL, 276 were annotated as hypothetical proteins and have been only derived from the genome sequence thus far. In the present study, 77 of these proteins were identified indicating that they are really expressed (Table S3). Functional characterization of these particular proteins will be a challenging task for future studies. In this context, expression data for these proteins are of special interest since they provide an informative basis for their function.

51 of these proteins showed a differential expression under at least one of the tested condition (2.5-fold change, statistically significant). For instance, SACOL0457 representing a “hypothetical protein” with no reasonable functional prediction was specifically induced in response to H_2_O_2_ stress. Additional uncharacterized proteins that may play an important role during adaptation to H_2_O_2_ exposure are SACOL1445 and SACOL1768. While SACOL1445 was predicted to be a member of a new class of chaperone systems mediating insertion of metal cofactors into substrate molecules [56], SACOL1768 displays homology to cGMP specific phosphodiesterases and may thus have a regulatory function. Up-regulation of SACOL1768 synthesis was not restricted to H_2_O_2_ exposure but was also found in response to paraquat, diamide, and nitric oxide. This suggests a more general role of the protein in oxidative stress adaptation and resistance. Interestingly, paraquat, diamide, and nitric oxide stress also caused a strong induction of SACOL0614 predicted to have GlcNAc deacetylase activity. The *B. subtilis* homolog of this protein, YojG, was recently renamed BshB2 and demonstrated to be essential for the synthesis of bacillithiol a low-molecular-weight thiol with a key role in the detoxification of electrophiles [57]. Further proteins identified with a possible role in the adaptation of *S. aureus* to oxidative stress are SACOL1447 (a putative glyoxalase) and SACOL2722 (a putative arylamine N-acetyltransferase).

A NifU homologue, SACOL0917, was also found to be induced in response to oxidative stress. The respective gene is part of a five gene cluster with a putative function in FeS center assembly. The present study revealed an increased expression of all members of the *SACOL0917* encoding operon (*SACOL0914*, *SACOL0915*, *SACOL0916*, *SACOL0917*, and *SACOL0918*) (http://www.aureolib.de/?m10), demonstrating the power of this integrative approach. In *B. subtilis*, a homologous system is involved in FeS center assembly [58]. Inspection of the regulatory region of the *S. aureus* operon revealed a putative regulatory binding site possibly recognized by PerR and/or Fur. Again this supports the critical role the system might play during H_2_O_2_ stress. In *B. subtilis* as well as in *S. aureus*, the system seems to be essential and might thus be an interesting target for new antibacterial substances [59], [60].

In response to diamide stress the synthesis of the bacterioferritin comigratory protein (Bcp) was up to 5-fold induced. The corresponding gene is co-transcribed with *fur* and *SACOL1920* which encode an iron-dependent regulator and a 2-hydroxyacid dehydrogenase, respectively. The staphylococcal Bcp protein shows 38% identity (55% similarity) to Bcp of *E. coli* which has been extensively investigated [61]. In *E. coli* Bcp contains three cysteine residues at positions 45, 50, and 99 whereas only the first cysteine is essential for peroxidase activity. Cys-45 and Cys-50 are also present in Bcp of *S. aureus*.

In addition to Bcp, six proteins were specifically induced by diamide treatment: YycF, SACOL0157, SACOL0467, SACOL0931, SACOL1894, and SACOL1891 implying a specific function for these proteins under thiol-oxidizing conditions. The very similar expression pattern of these proteins also suggests a common regulatory mechanism mediating gene expression in response to thiol oxidation (http://www.aureolib.de/?m11). Unfortunately, an alignment of upstream regions of the respective genes did not reveal the presence of common regulatory DNA motifs.

## Conclusions

With 4,692 time dependent expression profiles for 521 cytoplasmic proteins under nine infection relevant, well defined laboratory conditions *Aureolib* represents by far the most comprehensive protein expression database for *S. aureus*. We demonstrate here that *Aureolib* is a unique tool for (i) functional predictions of so far uncharacterized proteins based on their expression kinetics, (ii) the integration of stimulons into regulatory networks and (iii) for showing signal transduction systems operating under defined and also non-defined stress and starvation conditions. Moreover, the proteome signature library allows us to determine and visualize similarities between different signatures, and this can provide deeper insights into bacterial gene expression programs and signal transduction pathways. Surprisingly, cluster analyses revealed only in rare cases a grouping of protein synthesis profiles that correlated with known regulon structures (Fig. S5). These results suggest that synthesis of most proteins is under control of multiple transcriptional regulators. Furthermore, post-transcriptional events might play a more impressive role in gene expression in *S. aureus* than expected. These results clearly indicate that proteomics showing all proteins present under certain conditions represents the method of choice for a comprehensive understanding of cell physiology. However, for deciphering regulatory networks active under given environmental conditions, data on transcriptional and translational activities as well as information on the stability of both transcripts and proteins are required. Hence, only a combination of proteomics and transcriptomics will provide all essential information to successfully unravel signal transduction and regulatory mechanisms involved in gene expression in *S. aureus* under *in vitro* and *in vivo* conditions.

During colonization and infection, *S. aureus* is confronted with a multitude of signals including growth-limiting factors and life-threatening host defense mechanisms. Therefore, adaptation of bacterial gene expression in their natural habitat is a multi-signal response and it will be a challenging task for future studies to unravel this diverse network. Using *Aureolib* as a tool box, it is now possible to move to more complex, infection-relevant experimental models ranging from host cell tissue cultures, to various animal infection models and even to the analysis of *S. aureus* isolated from human body fluids and tissue samples. This should be accompanied by a further extension of the database by additional proteome signatures obtained under well defined host-mimicking conditions such as the presence of alternative carbon sources and antimicrobial peptides and nutrient limitations (e.g. carbon sources, iron). By the integration of *in vitro* and *in vivo* data, a new window of staphylococcal virulence and physiology will be opened. However, these data will not only enhance our understanding of *S. aureus* infection biology, but will also decipher the process of *S. aureus* nose colonization. This is of special importance, since about one-third of the human population is permanently colonized with *S. aureus* though usually showing no signs of disease. For systems biology approaches under defined laboratory and also under more complex infection conditions in the host protein information can be easily complemented by transcriptomic and metabolomic data generated under identical conditions.

## Materials and Methods

### Strains, growth, and stress conditions

Proteomic data used to establish the proteomic signature library are listed in table 1. All experiments were done with *S. aureus* COL grown in chemically defined medium (CDM) [62] with some modifications [63]. In each experiment cells were cultivated at 37°C under vigorous agitation starting at an optical density of 0.075 (λ = 500 nm; OD_500_). At OD_500_ 0.5 (exponential growth phase) cells were exposed to 3 µM puromycin or changed to 48°C. Other stressors were applied as described previously (see references in Table 1). In each case, the growth rate was reduced to at least 50% during the early phase of stress compared to the untreated culture which served as a control (Fig. S1). To monitor the synthesis of proteins, radioactively labeled L-[^35^S]-methionine was added to the control culture as well as to the stressed cultures at different time points after switching to growth restricting conditions (see Table S1 for details) [63].

### 2-dimensional (2D) SDS polyacrylamide gel electrophoresis (PAGE) and protein identification

Cytoplasmic proteins of *S. aureus* COL were separated using the 2D gel electrophoresis technique as previously described [63]. Briefly, 100 to 200 µg of the protein extracts were loaded onto commercially available IPG strips covering the p*I* range of 4–7. Afterwards, proteins were separated on polyacrylamide gels according to their molecular weight. The resulting gels were stained with the fluorescent dye Krypton™ according to the manufacturer's instructions (Thermo Scientific, Waltham (MA), USA) and scanned using Typhoon 9400 Variable Mode Imager. For protein identification, proteins of interest were cut from the gel using an Ettan spot picker (GE Healthcare, Little Chalfont, UK) with a picker head of 2 mm and transferred into 96-well microtiter plates. In-gel digestion and extraction of peptides were accomplished with the Ettan Spot Handling workstation (GE Healthcare, Little Chalfont, UK) using a protocol described by Eymann and coworkers [64]. Identification of *S. aureus* proteins by MALDI-TOF and MALDI-TOF-TOF MS was carried out as described previously [18]. MALDI-TOF-MS analyses of spotted peptide solutions were carried out on a Proteome-Analyzer 4800 (Applied Biosystems, Foster City, CA, USA). Spectra were recorded in reflector mode (mass range: 900–3700 Da). After calibration, peak lists were created using the “peak to MASCOT” script of the 4700 Explorer software. From the TOF-spectra the three strongest peaks were subjected to MALDI-TOF-TOF MS and calibration peak lists were created by using the script of the GPS Explorer™ Software Version 3.6. Peak lists were analyzed by using the MASCOT search engine (Matrix Science, London, UK), GPMAW 4.1 (Lighthouse data). Peptide mixtures that yielded at least twice a Mowse score of at least 49 and a sequence coverage of at least 30% were regarded as positive identifications. For a definite identification, each protein had to be identified at the same position on 2D gels using two biological replicates. Details on the mass spectrometry results for the identified proteins are presented in Table S4. All MS/MS data described in this paper are available through PRIDE [65] (http://tinyurl.com/73camxx, Accession numbers: 23457-24064). Identified protein spots were labeled with the respective protein names or locus numbers on the master gel. Multiple spots of the same protein were numbered consecutively. The master gel was used as a reference for spot identification on all images of the experiments.

### Radioactive labeling of newly synthesized proteins and 2D SDS PAGE

Newly synthesized proteins were visualized by L-[^35^S]-methionine pulse labeling at different time points after stress (Table S1) as described previously [18], [63]. Briefly, L-[^35^S]-methionine was added to the culture and thereby incorporated into newly synthesized proteins. The labeling reaction was stopped after five minutes by adding chloramphenicol (0.1 mg/ml) and unlabeled L-methionine (1 mM) and by transferring samples to ice. After centrifugation (5 min, 21,000× g, 4°C) cell pellets were washed twice with TE buffer (10 mM Tris HCl, 1 mM EDTA, pH 7.5) and resuspended in 400 µl TE buffer. For cell lysis, cells were pretreated with lysostaphin (0.025 mg/ml, 10 minutes on ice) and subsequently disrupted by sonication. Cell debris was removed by two centrifugation steps (first step 10 min, 21,000× g, 4°C; second step 30 min, 21,000× g, 4°C). Protein concentration was measured using Roti Nanoquant (Roth, Karlsruhe, Germany). 2D SDS PAGE was performed as described [66]. The resulting gels were stained either with silver nitrate or the fluorescent dye Krypton™ according to the manufacturer's instructions (Thermo Scientific, Waltham (MA), USA) and scanned using Typhoon 9400 Variable Mode Imager. Protein gels were then dried on a vacuum dryer and fixed onto Whatman paper. Dried gels were exposed to storage phosphor screens (Molecular Dynamics, Krefeld, Germany) which were subsequently scanned using a Typhoon 9400 Variable Mode Imager.

### Analysis of protein synthesis patterns

#### Quantification of protein spots

All experiments were analyzed using Delta2D 4.0 (Decodon GmbH, Greifswald, Germany). First, gel to gel variations of spot positions on 2D gel images (autoradiograms) were compensated by warping (exact mode) of all images within each stress experiment. Subsequently, images were fused intra-experimentally to a virtual fusion gel (union mode). This fusion gel was used for protein spot detection. Automatically calculated spot borders were corrected and optimized manually if necessary. The final spot mask was transferred from the fusion gel to each gel image of the corresponding experiment (for further details see [67]).

#### Data transformation

Spot intensities were normalized using total normalization. For each protein spot, synthesis ratios of stressed samples and unstressed control samples were calculated and presented as logarithm to the base 2 values.

For statistical analyses relative spot volumes were standardized using z-score. Standardization was performed using Microsoft Excel.

#### Statistical analyses

For statistical analyses standardized data were loaded into TIGR Multiexperiment Viewer 4.4.1 [68]. Each experiment was analyzed separately. To test whether the synthesis rate of a given protein spot changes in response to stress we used one-way ANOVA (α = 0.1, distribution based on 1000 permutations). Significantly changed synthesis profiles were subsequently used for t-test analysis (α = 0.1, distribution based on all possible permutations, adjusted Bonferroni correction) to compare the standardized spot volumes of stressed samples at each time point with those of the control sample at t_0_. Only protein spots whose synthesis changed at least 2.5-fold were considered as differentially expressed. Hierarchical sample clustering (HCL) performed in this study is based on complete linkages and Euclidean distances.

### Inter-experimental analysis

For the inter-experimental analysis, we used the previously described identification-based approach to fuse the expression profiles of different experiments [37]. Briefly, 728 protein identifications were manually transferred from the master gel to the experiment-specific fusion gels (see above). Position of transferred spot labels were checked and corrected if necessary. In case of failed spot identifications due to variations of spot positions the respective expression profiles of the involved protein spots are missing in the database presentation. In some experiments, gel to gel variations result in co-localization of two proteins in one protein spot. As a consequence identification of the involved spots can vary between the individual experiments and the identification-based operation failed. In these cases, expression data of the protein spots is presented for each protein identification. All information regarding experiment-specific co-localizations of proteins are stored and highlighted in the respective expression profile.

### Protein annotation and regulon structure information

Annotation and functional classification of proteins is based on The Comprehensive Microbial Resource [69] (http://cmr.jcvi.org/tigr-scripts/CMR/CmrHomePage.cgi) with some modifications. Data on the regulon structure was derived from the Regprecise database [31], [32], [33] (http://regprecise.lbl.gov) based on the *S. aureus* N315 genome sequence information. Corresponding *S. aureus* COL genes were determined using NCBI's GenePlot.

## Supporting Information

Figure S1 **Growth of** ***S. aureus*** **COL in response to different growth restricting conditions.** Cells were grown in synthetic medium at 37°C to an optical density of 0.5 at 500 nm. Subsequently 10 ml of the culture were exposed to the different stress conditions (• control culture, ° stressed culture).
(PDF)

Figure S2 **Reference map of cytoplasmic proteins of** ***S. aureus*** **COL.** Cells were grown aerobically in chemically defined medium at 37°C to an optical density of 0.5 at 500 nm (OD500). Cytoplasmic proteins were separated on 2D gels in a p*I* range of 4–7. Proteins were stained with Krypton™ Protein Stain (Thermo Scientific). The reference gel is presented in four sections (1, 2, 3, and 4). In total, 679 protein spots were identified by MALDI-TOF MS/MS. We obtained 728 protein identifications resulting in 521 protein species. Identified protein spots are labeled with the respective gene symbol or locus tag as listed in Table S2. Multiple spots of the same protein were numbered.
(PDF)

Figure S3 **The theoretical cytoplasmic proteome of** ***S. aureus*** **COL.** Theoretical molecular weights and isoelectric points of all predicted cytoplasmic proteins of *S. aureus* COL were calculated from the amino acid sequences. The analytical window of the 2D gel analysis is highlighted in grey. Proteins identified on the reference gel are shown in blue and orange. Proteins in blue were identified in one protein spot while those shown in orange appeared as multiple spots on the reference gel. Proteins that were not identified on the gel appear in grey.
(PDF)

Figure S4 **Functional categories of the identified proteins.** The circle diagram shows the number of the identified proteins belonging to the indicated functional groups. A more detailed functional classification of proteins belonging to the functional groups (i) stress response (orange), (ii) gene expression (red), (iii) metabolism (green), and (iv) biosynthetic pathways (blue) is presented in bar charts.
(PDF)

Figure S5 **Comparison of synthesis profiles of differently expressed proteins in the library.** Hierarchical clustering (Euclidian distance/complete linkage) of synthesis profiles of proteins showing significant changes (≥2.5-fold) in response to at least one stimulus applied in the present study.
(PNG)

Figure S6 **The CodY regulon.** Hierarchical clustering (Euclidian distance/complete linkage) of synthesis profiles of CodY dependent proteins in response to different stimuli.
(PNG)

Figure S7 **The hydrogen peroxide stimulon.** Hierarchical clustering (Euclidian distance/complete linkage) of synthesis profiles of proteins significantly changed (≥2.5-fold) in response to hydrogen peroxide.
(PNG)

Table S1 Sample points for proteome analyses of the different experiments.
(PDF)

Table S2 Proteins identified on the reference map.
(PDF)

Table S3 Hypothetical proteins identified on the reference map.
(PDF)

Table S4 Mass spectrometry results for the identified proteins.
(PDF)
