# Supplementary material for: Aureolib — A Proteome Signature Library: Towards an Understanding of Staphylococcus aureus Pathophysiology
Source: PLoS One. 2013 Aug 13;8(8):e70669. doi: 10.1371/journal.pone.0070669 (PMC3742771; doi:10.1371/journal.pone.0070669)
Supplement: Figure S1 — Growth of S. aureus COL in response to different growth restricting conditions. Cells were grown in synthetic medium at 37°C to an optical density of 0.5 at 500 nm. Subsequently 10 ml of the culture were exposed to the different stress conditions (• control culture, ° stressed culture). (PDF) [file pone.0070669.s001.pdf]

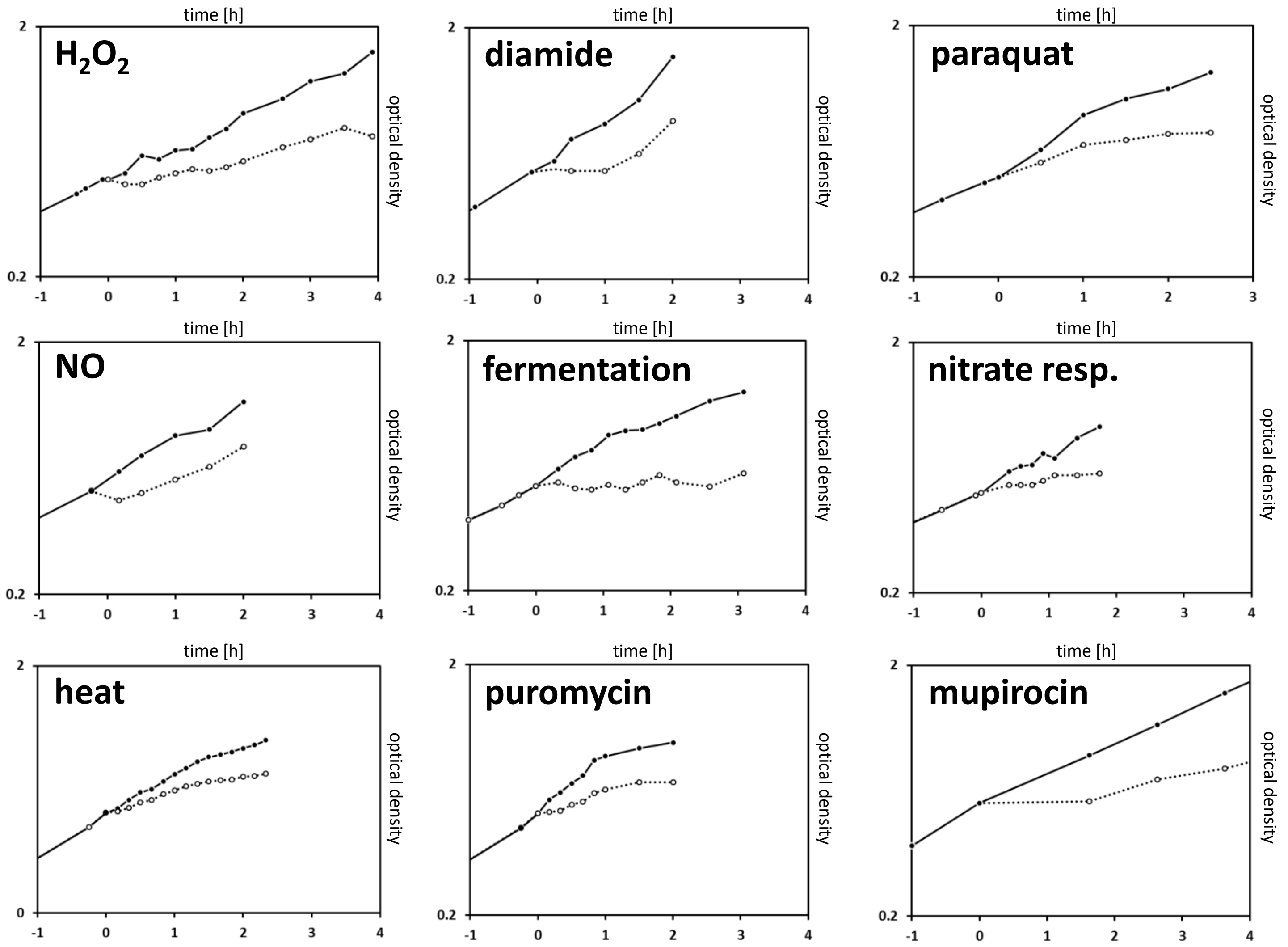

Supplementary Fig. S1: Growth of *S. aureus* COL in response to different growth restricting conditions. Cells were grown in synthetic medium at 37°C to an optical density of 0.5 at 500 nm. Subsequently 10 ml of the culture were exposed to the different stress conditions (• control culture,˚ stressed culture).
