# Supplementary material for: Aureolib — A Proteome Signature Library: Towards an Understanding of Staphylococcus aureus Pathophysiology
Source: PLoS One. 2013 Aug 13;8(8):e70669. doi: 10.1371/journal.pone.0070669 (PMC3742771; doi:10.1371/journal.pone.0070669)
Supplement: Figure S2 — Reference map of cytoplasmic proteins of S. aureus COL. Cells were grown aerobically in chemically defined medium at 37°C to an optical density of 0.5 at 500 nm (OD500). Cytoplasmic proteins were separated on 2D gels in a pI range of 4–7. Proteins were stained with Krypton™ Protein Stain (Thermo Scientific). The reference gel is presented in four sections (1, 2, 3, and 4). In total, 679 protein spots were identified by MALDI-TOF MS/MS. We obtained 728 protein identifications resulting in 521 protein species. Identified protein spots are labeled with the respective gene symbol or locus tag as listed in Table S2. Multiple spots of the same protein were numbered. (PDF) [file pone.0070669.s002.pdf]

1

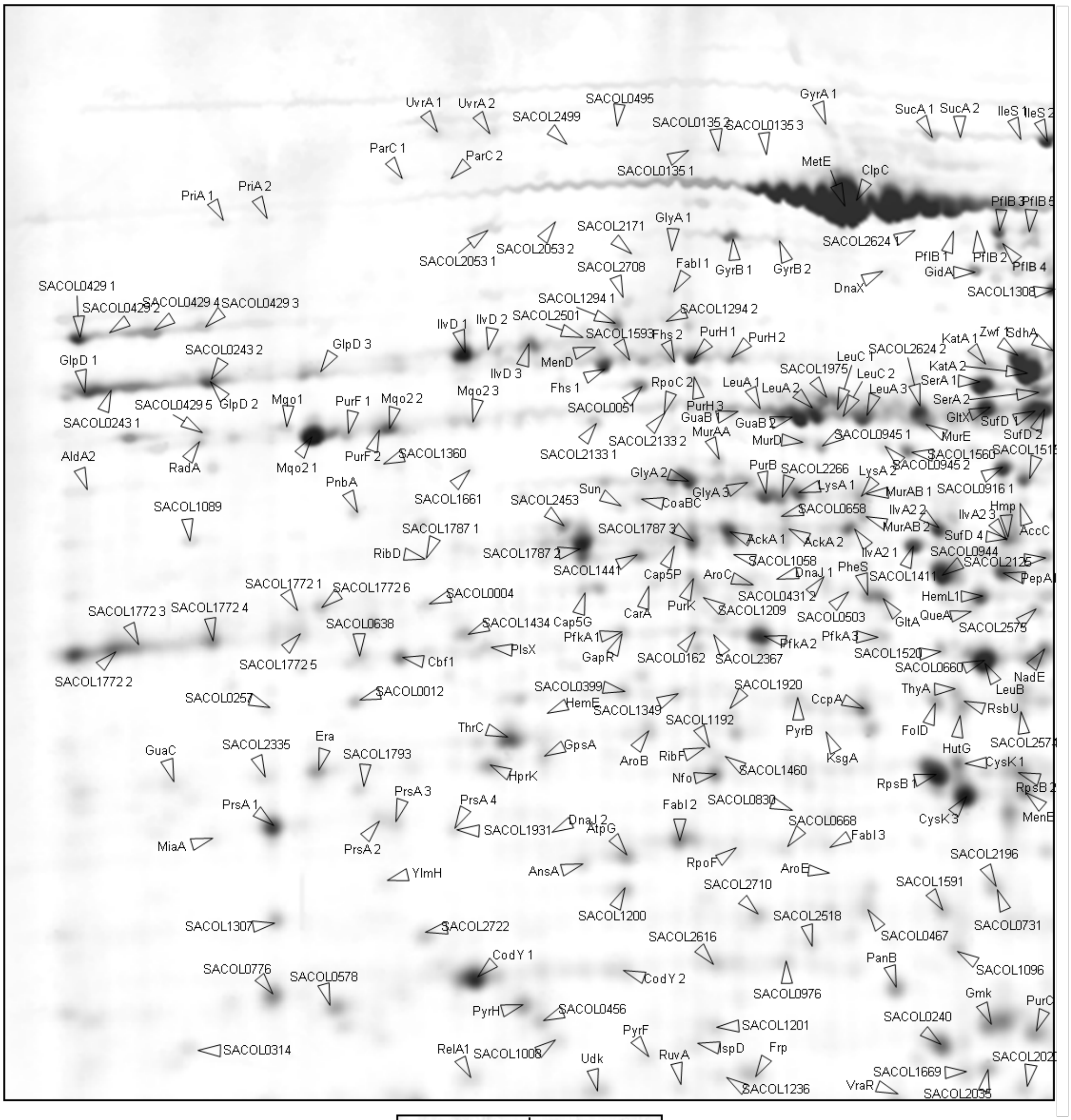
UvrA 1
UvrA 2
SACOL0495
GyrA 1
SucA 1
SucA 2
IleS 1
IleS 2
SACOL2499
SACOL0135 2
SACOL0135 3
ParC 1
ParC 2
MetE
ClpC
SACOL0135 1
PriA 1
PriA 2
PflB 3
PflB 5
GlyA 1
SACOL2171
SACOL2624 1
SACOL2053 2
SACOL2053 1
PflB 1
PflB 2
PflB 4
GidA
SACOL2708
FabI 1
GyrB 1
GyrB 2
DnaX
SACOL1308
SACOL0429 1
SACOL1294 1
SACOL1294 2
SACOL0429 2
SACOL0429 4
SACOL0429 3
SACOL2501
IlvD 1
IlvD 2
SACOL1593
Fhs 2
PurH 1
PurH 2
KatA 1
Zwf 1
SdhA
GlpD 3
SACOL2624 2
SACOL0243 2
GlpD 1
MenD
LeuC 1
KatA 2
IlvD 3
SACOL1975
LeuC 2
SerA 1
Fhs 1
RpoC 2
LeuA 1
LeuA 2
LeuA 3
Mqo2 3
SerA 2
SACOL0429 5
GlpD 2
Mqo1
PurF 1
Mqo2 2
SACOL0051
PurH 3
GltX
SufD 1
GuaB 1
GuaB 2
SACOL0243 1
MurE
SufD 2
MurAA
SACOL2133 2
MurD
SACOL0945 1
SACOL1560
SACOL151
AldA2
RadA
Mqo2 1
PurF 2
SACOL1360
SACOL2133 1
PurB
SACOL2266
LysA 2
SACOL0945 2
GlyA 2
LysA 1
PnbA
Sun
GlyA 3
MurAB 1
SACOL0916 1
SACOL1661
SACOL2453
CoaBC
SACOL0658
IlvA2 2
IlvA2 3
Hmp
SACOL1089
SACOL1787 1
SACOL1787 3
MurAB 2
AccC
AckA 1
AckA 2
SufD 4
RibD
SACOL1787 2
IlvA2 1
SACOL0944
SACOL1058
SACOL2125
SACOL1441
Cap5P
AroC
DnaJ 1
PheS
SACOL1411
PepA
SACOL1772 1
SACOL1772 6
SACOL0004
SACOL0431 2
HemL1
PurK
CarA
SACOL1209
SACOL1772 3
SACOL1772 4
SACOL0503
GltA
QueA
SACOL0638
SACOL1434
Cap5G
SACOL2575
PfkA 1
PfkA2
PfkA3
PlsX
Cbf1
GapR
SACOL0162
SACOL2367
SACOL1520
SACOL1772 5
SACOL0660
SACOL1772 2
NadE
SACOL0399
SACOL1920
ThyA
LeuB
SACOL0012
SACOL0257
HemE
SACOL1349
CcpA
RsbU
SACOL1192
PyrB
FolD
ThrC
HutG
SACOL2574
Era
SACOL2335
GuaC
SACOL1793
GpsA
AroB
RibF
KsgA
CysK 1
SACOL1460
HprK
Nfo
RpsB 1
RpsB 2
PrsA 3
PrsA 4
FabI 2
SACOL0830
CysK 3
MenE
PrsA 1
DnaJ 2
AtpG
SACOL1931
SACOL0668
FabI 3
MiaA
PrsA 2
SACOL2196
RpoF
AroE
YlmH
AnsA
SACOL2710
SACOL1591
SACOL1200
SACOL2518
SACOL0731
SACOL1307
SACOL2722
SACOL0467
SACOL2616
CodY 1
PanB
SACOL1096
SACOL0776
SACOL0578
CodY 2
SACOL0976
Gmk
PurC
PyrH
SACOL0456
SACOL0240
PyrF
SACOL1201
SACOL0314
RelA1
SACOL1008
Udk
RuvA
IspD
Frp
SACOL202
SACOL1669
VraR
SACOL1236
SACOL2035

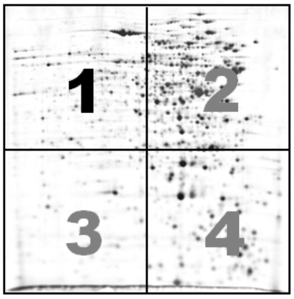
1
2
3
4

2

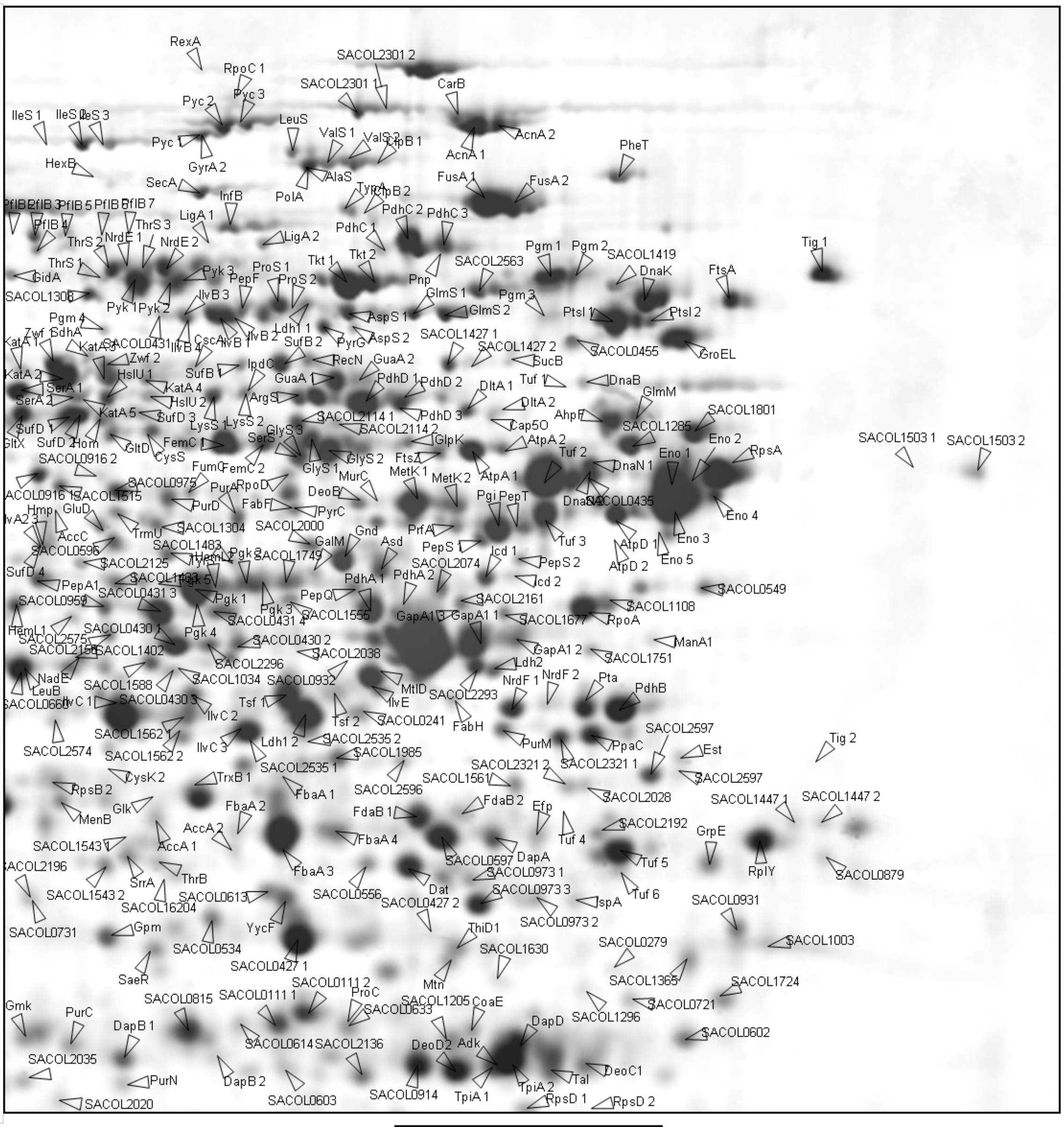
RexA
RpoC 1
SACOL2301 2
SACOL2301
CarB
Pyc 2
Pyc 3
IleS 1
IleS 2
IleS 3
LeuS
AcnA 2
Pyc 1
ValS 1
ValS 2
ClpB 1
GyrA 2
AcnA 1
PheT
HexB
AlaS
FusA 1
FusA 2
SecA
InfB
PolA
TyrA
ClpB 2
PflB 2
PflB 3
PflB 5
PflB 6
PflB 7
LigA 1
PdhC 2
PdhC 3
PflB 4
ThrS 3
PdhC 1
ThrS 2
NrdE 1
NrdE 2
LigA 2
Tig 1
Pgm 1
Pgm 2
SACOL1419
ThrS 1
Tkt 1
Tkt 2
SACOL2563
DnaK
FtsA
GidA
Pyk 3
ProS 1
PepF
ProS 2
Pnp
SACOL1308
GlmS 1
Pgm 3
IlvB 3
Pyk 1
Pyk 2
GlmS 2
PtsI 1
PtsI 2
AspS 1
Pgm 4
Ldh1 1
SdhA
Zwf
KatA 1
KatA 3
SACOL0431
CscA
IlvB 1
IlvB 2
PyrG
AspS 2
SACOL1427 1
IlvB 4
SufB 2
SACOL1427 2
SACOL0455
GroEL
Zwf 2
RecN
GuaA 2
SucB
IpdC
KatA 2
HslU 1
SufB 1
GuaA 1
PdhD 1
PdhD 2
DltA 1
Tuf 1
DnaB
GlmM
SerA 1
KatA 4
SerA 2
HslU 2
ArgS
DltA 2
KatA 5
SufD 3
SACOL2114 1
PdhD 3
AhpF
SACOL1801
LysS 1
LysS 2
SACOL2114 2
Cap5O
SufD 1
GlyS 3
SACOL1285
GltX
SufD 2
Hom
GltD
FemC 1
SerS
GlpK
AtpA 2
Eno 2
RpsA
SACOL1503 1
SACOL1503 2
CysS
GlyS 2
FtsZ
Tuf 2
Eno 1
SACOL0916 2
FumC
FemC 2
GlyS 1
MurC
MetK 1
DnaN 1
SACOL0975
PurA
RpoD
MetK 2
AtpA 1
SACOL0916 1
SACOL1515
DeoB
Pgi
PepT
DnaN 2
SACOL0435
Hmp
GluD
PurD
FabF
PyrC
Eno 4
IlvA2 3
SACOL1304
SACOL2000
PrfA
Tuf 3
Eno 3
AccC
TrmU
Gnd
GalM
Asd
PepS 1
Icd 1
AtpD 1
SACOL0596
SACOL1483
Eno 5
SACOL2125
HemL 2
Pgk 2
SACOL1749
SACOL2074
PepS 2
AtpD 2
SufD 4
SACOL1433
TyrS
Pgk 5
PdhA 1
PdhA 2
Icd 2
PepA1
SACOL0431 3
SACOL0549
SACOL0959
Pgk 1
PepQ
SACOL2161
SACOL1108
Pgk 3
SACOL1555
SACOL0431 4
GapA1 3
GapA1 1
SACOL1677
RpoA
HemL 1
SACOL0430 1
Pgk 4
ManA1
SACOL2575
SACOL0430 2
SACOL2158
SACOL1402
SACOL2038
GapA1 2
SACOL1751
SACOL2296
Ldh2
NadE
SACOL1034
SACOL0932
MtlD
NrdF 1
NrdF 2
Pta
SACOL1588
LeuB
IlvE
SACOL2293
PdhB
SACOL0660
IlvC 1
SACOL0430 3
Tsf 1
IlvC 2
Tsf 2
SACOL0241
FabH
SACOL2597
SACOL1562 1
IlvC 3
Ldh1 2
SACOL2535 2
PurM
PpaC
Est
Tig 2
SACOL2574
SACOL1562 2
SACOL1985
SACOL2321 2
SACOL2321 1
SACOL2535 1
CysK 2
TrxB 1
SACOL1561
SACOL2597
RpsB 2
FbaA 1
SACOL2596
SACOL2028
SACOL1447 1
SACOL1447 2
Glk
FbaA 2
FdaB 2
Efp
MenB
FdaB 1
SACOL2192
AccA 2
GrpE
SACOL1543 1
AccA 1
FbaA 4
Tuf 4
DapA
SACOL2196
SACOL0597
Tuf 5
RplY
SrrA
ThrB
FbaA 3
SACOL0973 1
SACOL0879
Dat
SACOL1543 2
SACOL0613
SACOL0556
SACOL0427 2
SACOL0973 3
Tuf 6
IspA
SACOL0931
SACOL16204
SACOL0973 2
SACOL0731
Gpm
YycF
ThiD1
SACOL0534
SACOL1630
SACOL0279
SACOL1003
SACOL0427 1
SaeR
Mtn
SACOL1365
SACOL1724
SACOL0111 2
SACOL0815
SACOL0111 1
ProC
SACOL1205
CoaE
SACOL1296
SACOL0721
Gmk
PurC
SACOL0633
DapB 1
DapD
SACOL0602
SACOL2035
SACOL0614
SACOL2136
DeoD2
Adk
DeoC1
PurN
DapB 2
Tal
SACOL0603
SACOL0914
TpiA 1
TpiA 2
SACOL2020
RpsD 1
RpsD 2

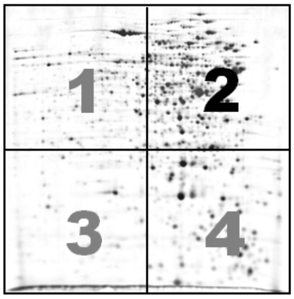
1
2
3
4

Supplementary Figure S2

3

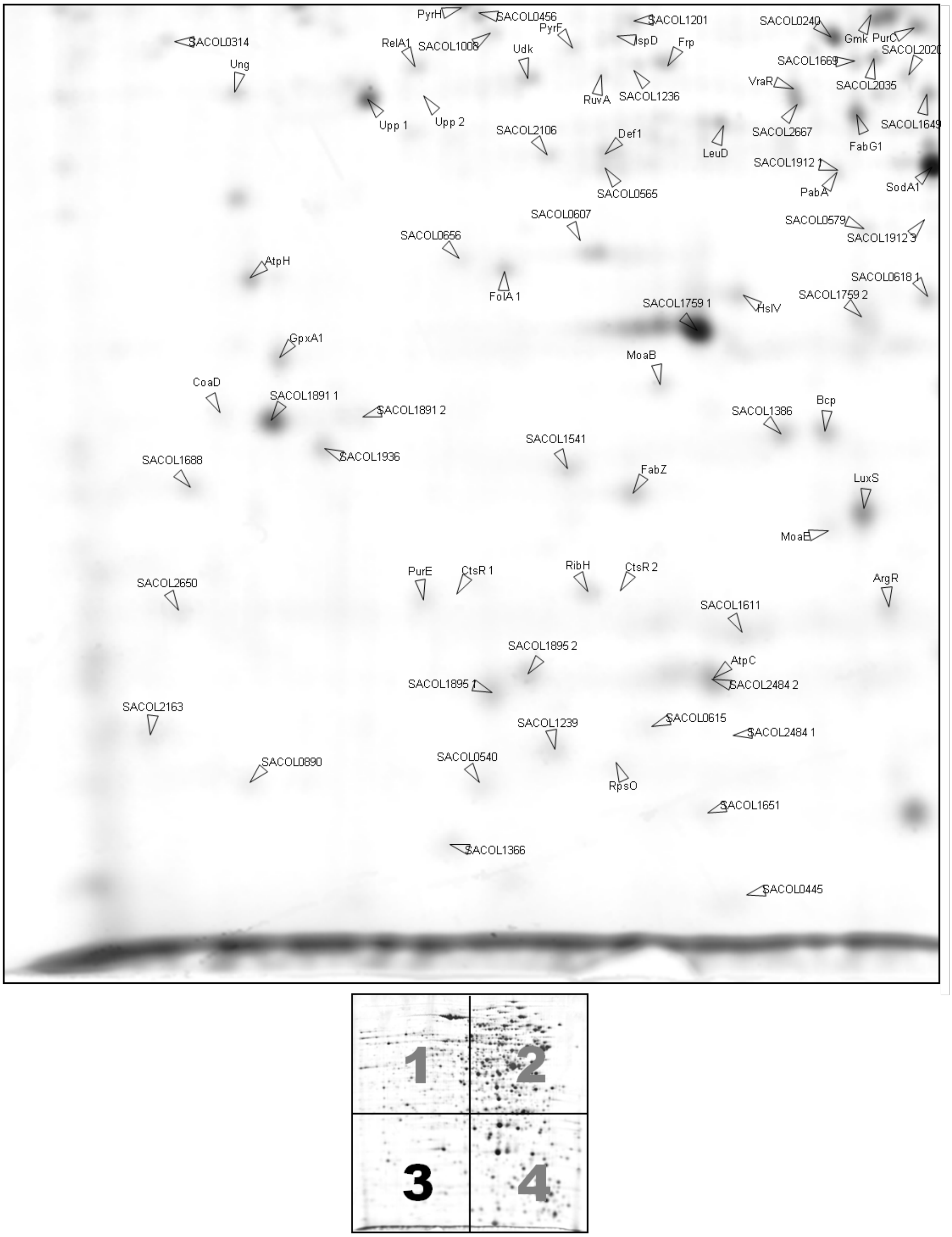

4

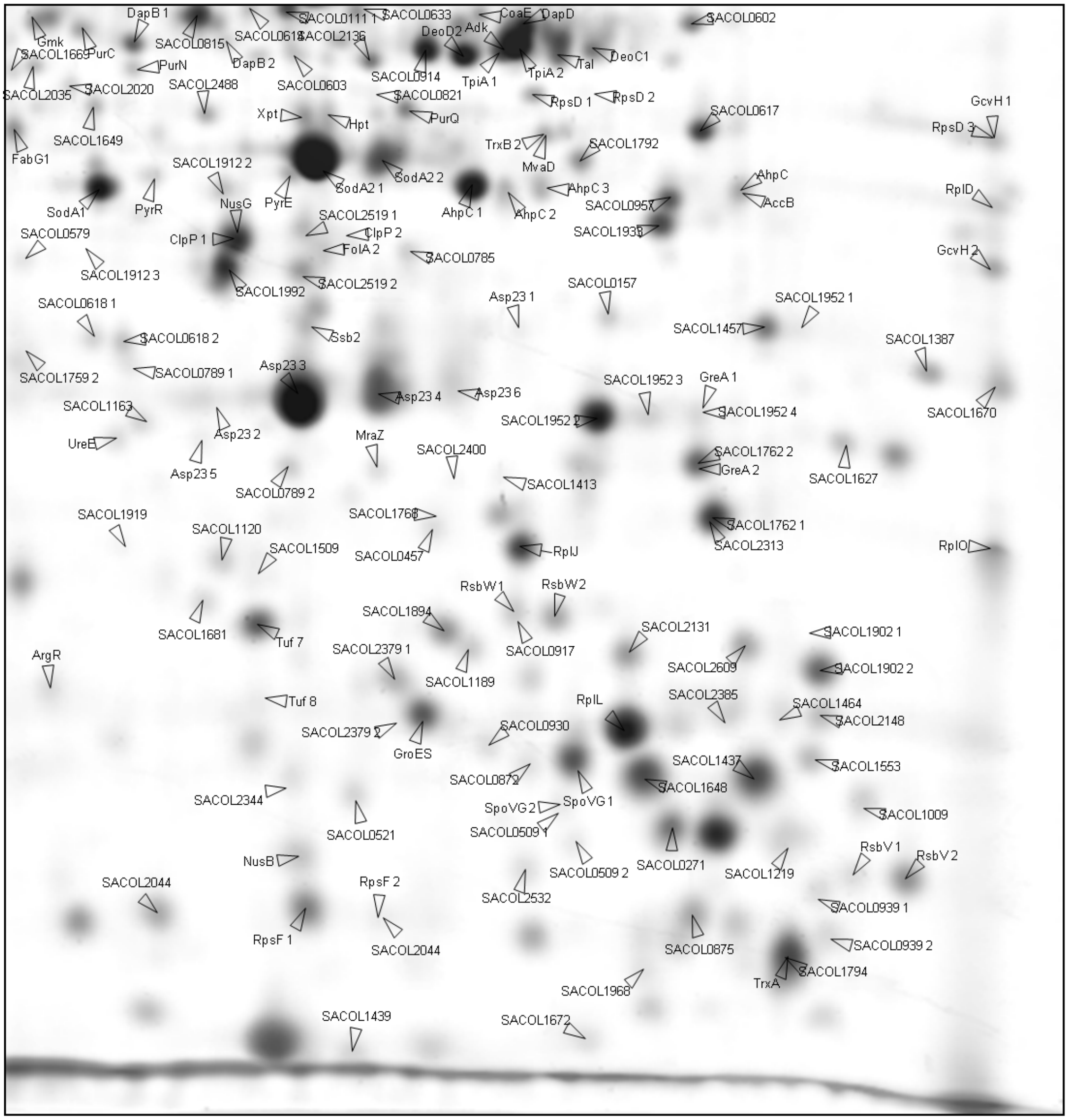

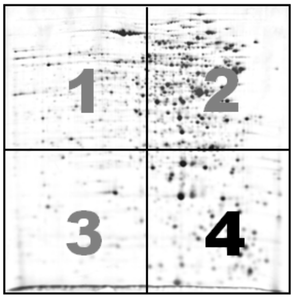

Supplementary Fig. S2: Reference map of cytoplasmic proteins of *S. aureus* COL. Cells were grown aerobically in chemically defined medium at 37°C to an optical density of 0.5 at 500 nm (OD_{500}). Cytoplasmic proteins were separated on 2D gels in a pI range of 4-7. Proteins were stained with Krypton^{TM} Protein Stain (Thermo Scientific). The reference gel is presented in four sections (1, 2, 3, and 4). In total, 679 protein spots were identified by Maldi-TOF MS/MS. We obtained 728 protein identifications resulting in 521 protein species. Identified protein spots are labeled with the respective gene symbol or locus tag as listed in the Supporting Information Table S2. Multiple spots of the same protein were numbered.
