# Supplementary material for: Aureolib — A Proteome Signature Library: Towards an Understanding of Staphylococcus aureus Pathophysiology
Source: PLoS One. 2013 Aug 13;8(8):e70669. doi: 10.1371/journal.pone.0070669 (PMC3742771; doi:10.1371/journal.pone.0070669)
Supplement: Figure S3 — The theoretical cytoplasmic proteome of S. aureus COL. Theoretical molecular weights and isoelectric points of all predicted cytoplasmic proteins of S. aureus COL were calculated from the amino acid sequences. The analytical window of the 2D gel analysis is highlighted in grey. Proteins identified on the reference gel are shown in blue and orange. Proteins in blue were identified in one protein spot while those shown in orange appeared as multiple spots on the reference gel. Proteins that were not identified on the gel appear in grey. (PDF) [file pone.0070669.s003.pdf]

Supplementary Figure S3

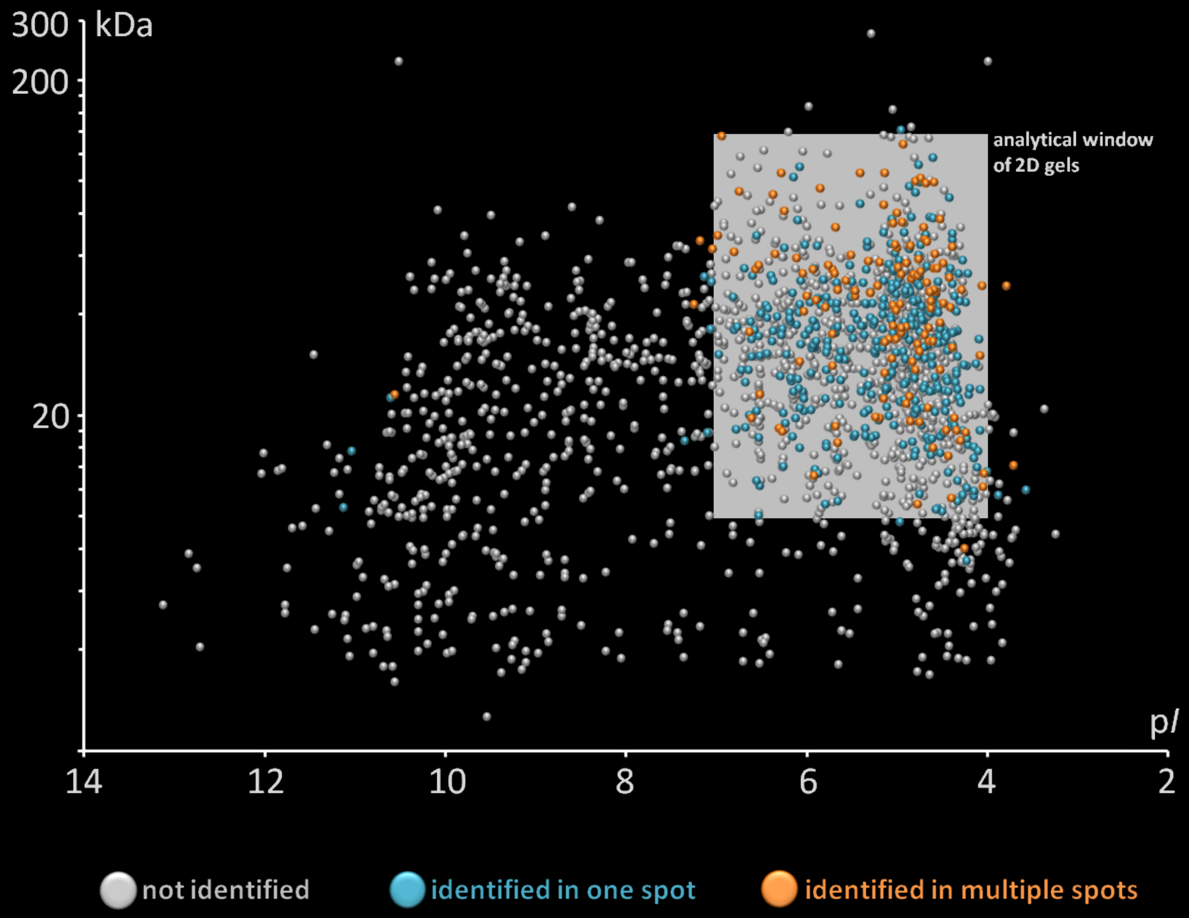

Supplementary Fig. S3: The theoretical cytoplasmic proteome of *S. aureus* COL. Theoretical molecular weights and isolectric points of all predicted cytoplasmic proteins of *S. aureus* COL were calculated from the amino acid sequences. The analytical window of the 2D gel analysis is highlighted in grey. Proteins identified on the reference gel are shown in blue and orange. Proteins in blue were identified in one protein spot while those shown in orange appeared as multiple spots on the reference gel. Proteins that were not identified on the gel appear in grey.
