# Supplementary material for: Aureolib — A Proteome Signature Library: Towards an Understanding of Staphylococcus aureus Pathophysiology
Source: PLoS One. 2013 Aug 13;8(8):e70669. doi: 10.1371/journal.pone.0070669 (PMC3742771; doi:10.1371/journal.pone.0070669)
Supplement: Figure S4 — Functional categories of the identified proteins. The circle diagram shows the number of the identified proteins belonging to the indicated functional groups. A more detailed functional classification of proteins belonging to the functional groups (i) stress response (orange), (ii) gene expression (red), (iii) metabolism (green), and (iv) biosynthetic pathways (blue) is presented in bar charts. (PDF) [file pone.0070669.s004.pdf]

Supplementary Figure S4

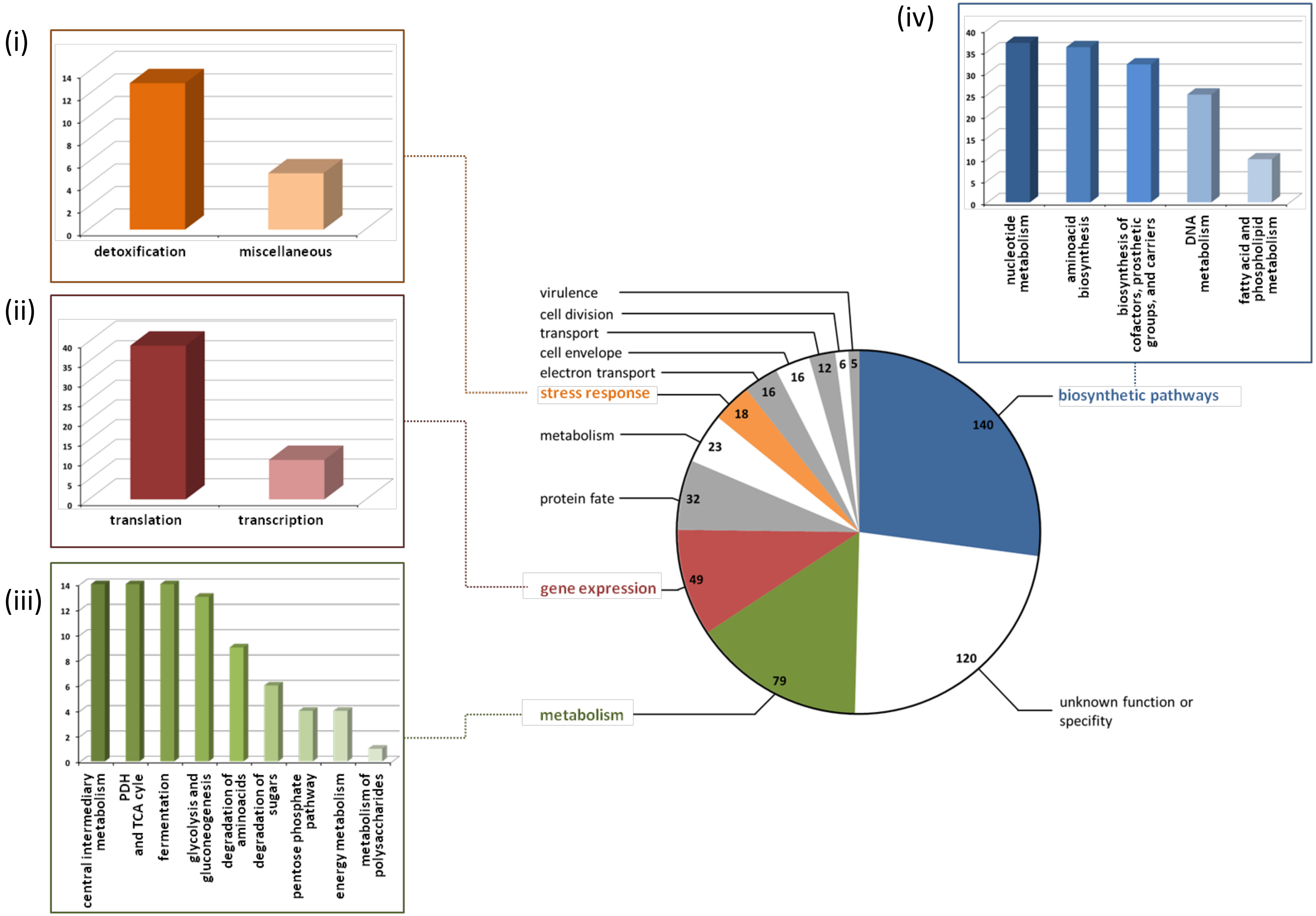

Supplementary Fig. S4: Functional categories of the identified proteins. The circle diagram shows the number of the identified proteins belonging to the indicated functional groups. A more detailed functional classification of proteins belonging to the functional groups (i) stress response (orange), (ii) gene expression (red), (iii) metabolism (green), and (iv) biosynthetic pathways (blue) is presented in bar charts.
