# Supplementary figures and images for: Aureolib — A Proteome Signature Library: Towards an Understanding of Staphylococcus aureus Pathophysiology
Source: PLoS One. 2013 Aug 13;8(8):e70669. doi: 10.1371/journal.pone.0070669 (PMC3742771; doi:10.1371/journal.pone.0070669)

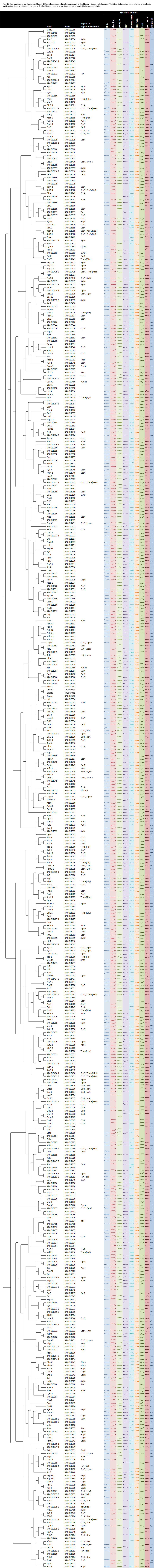

Supplement: Figure S5 — Comparison of synthesis profiles of differently expressed proteins in the library. Hierarchical clustering (Euclidian distance/complete linkage) of synthesis profiles of proteins showing significant changes (≥2.5-fold) in response to at least one stimulus applied in the present study. (PNG) [file pone.0070669.s005.png]

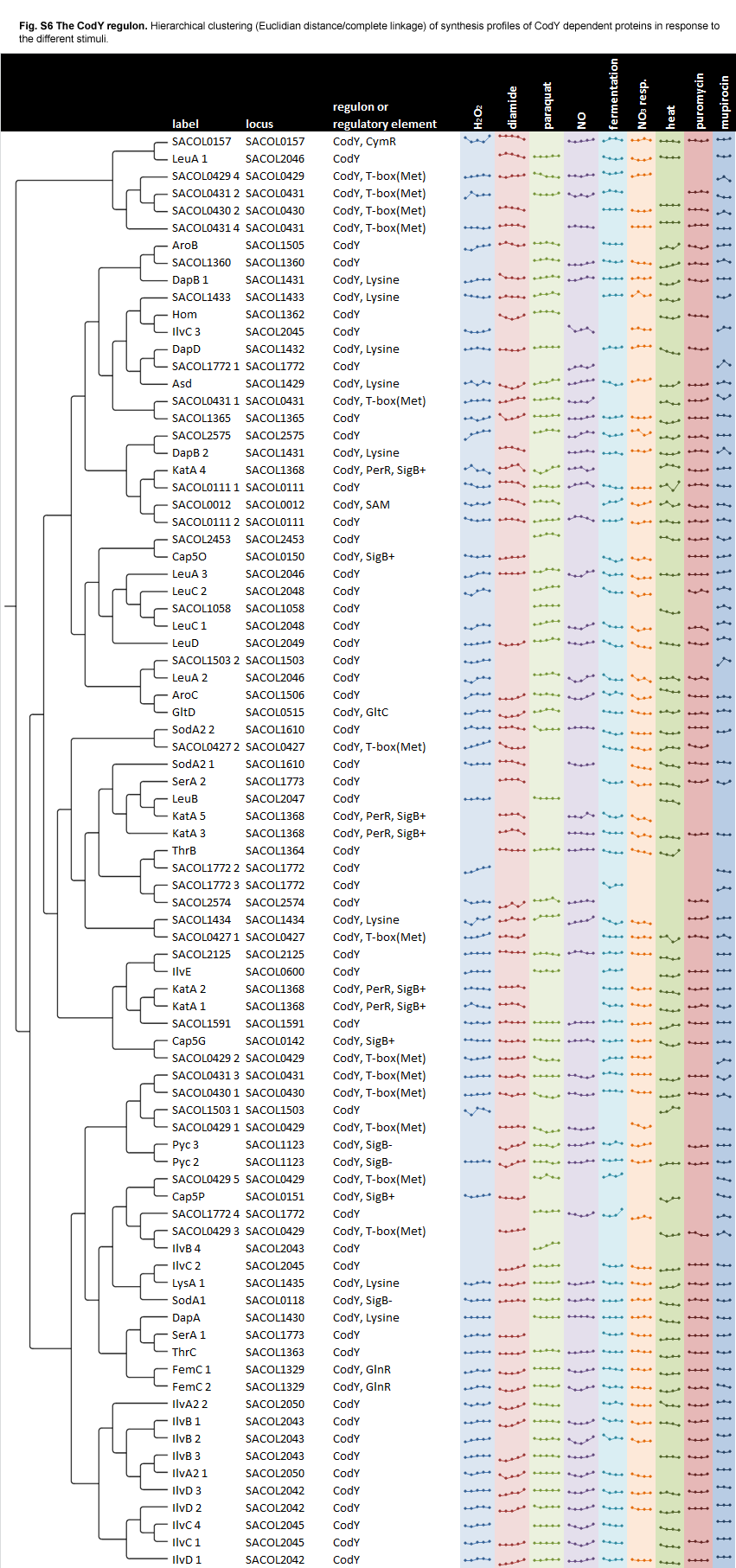

Supplement: Figure S6 — The CodY regulon. Hierarchical clustering (Euclidian distance/complete linkage) of synthesis profiles of CodY dependent proteins in response to different stimuli. (PNG) [file pone.0070669.s006.png]

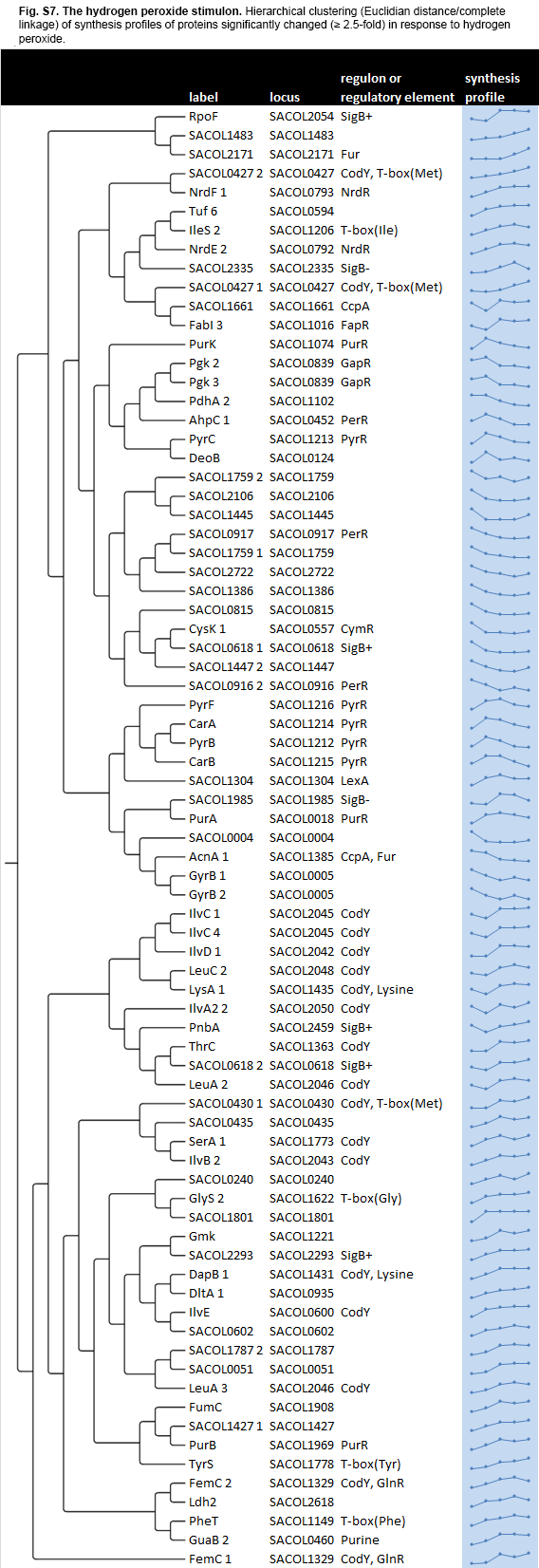

Supplement: Figure S7 — The hydrogen peroxide stimulon. Hierarchical clustering (Euclidian distance/complete linkage) of synthesis profiles of proteins significantly changed (≥2.5-fold) in response to hydrogen peroxide. (PNG) [file pone.0070669.s007.png]
