# Supplementary material for: Aureolib — A Proteome Signature Library: Towards an Understanding of Staphylococcus aureus Pathophysiology
Source: PLoS One. 2013 Aug 13;8(8):e70669. doi: 10.1371/journal.pone.0070669 (PMC3742771; doi:10.1371/journal.pone.0070669)
Supplement: Table S1 — Sample points for proteome analyses of the different experiments. (PDF) [file pone.0070669.s008.pdf]

Supplementary Table S1. Sample points for proteome analyses of the different experiments.

**experiment**	**control samples[1]**		**stressed samples[1]**							
	**0‘**	**60‘**	**1‘**	**5‘**	**10‘**	**20‘**	**30‘**	**40‘**	**60‘**	**120‘**
hydrogen peroxide	●●			●●	●●		●●	●●	●●	
diamide	●●			●●	●●		●●	●●	●●	
paraquat	●●			●●	●●		●●		●●	●●
nitric oxide	●●		●●	●●	●●		●●		●●	
fermentation	●●	●●			●●	●●	●●		●●	
nitrate respiration	●●	●●			●●	●●	●●		●●	
heat	●●●			●●●	●●●		●●		●●●	
puromycin	●●●	●●●		●●●	●●●		●●●		●●●	
mupirocin	●●				●●		●●		●●	

[1] Samples were harvested at the indicated time points (in minutes) before and after exposing the culture to the different conditions at OD_{500nm} 0.5 (number of • indicates the number of biological replicates).
