# Supplementary material for: Aureolib — A Proteome Signature Library: Towards an Understanding of Staphylococcus aureus Pathophysiology
Source: PLoS One. 2013 Aug 13;8(8):e70669. doi: 10.1371/journal.pone.0070669 (PMC3742771; doi:10.1371/journal.pone.0070669)
Supplement: Table S2 — Proteins identified on the reference map. (PDF) [file pone.0070669.s009.pdf]

Supplementary Table S2. Proteins identified on the reference map

#	locus[1]	AC[2]	protein symbol	protein spots[3]	description	pI	MW [kDa]
1	SACOL0002	Q5HJZ4	DnaN	1	DNA polymerase III, beta subunit	4.4	41.91
2	SACOL0004	Q5HJZ2	-	1	recF protein	6.45	42.42
3	SACOL0005	Q5HJZ1	GyrB	1	DNA gyrase, B subunit	5.68	72.51
4	SACOL0006	Q5HJZ0	GyrA	1	DNA gyrase, A subunit	4.8	99.35
5	SACOL0009	Q5HJY7	SerS	1	seryl-tRNA synthetase	4.75	48.64
6	SACOL0012	Q5HJY4	-	1	homoserine O-acetyltransferase, putative	6.62	37.85
7	SACOL0016	Q5HJY0	DnaB	1	replicative DNA helicase	4.42	52.57
8	SACOL0018	Q5HJX8	PurA	1	adenylosuccinate synthetase	4.86	47.58
9	SACOL0019	Q5HJX7	YycF	1	DNA-binding response regulator YycF	4.81	27.19
10	SACOL0051	Q5HJU6	-	2	conserved hypothetical protein	5.88	63.74
11	SACOL0111	Q5HJP2	-	1	acetoin reductase	4.79	27.22
12	SACOL0118	Q5HJN5	SodA1	1	superoxide dismutase	5.28	23.04
13	SACOL0123	Q5HJN0	DeoC1	2	deoxyribose-phosphate aldolase	4.43	23.47
14	SACOL0124	Q5HJM9	DeoB	2	phosphopentomutase	4.77	43.8
15	SACOL0135	Q5HJM2	-	1	alcohol dehydrogenase, iron-containing	5.85	94.94
16	SACOL0142	Q5HJL5	Cap5G	1	UDP-N-acetylglucosamine 2-epimerase Cap5G	6.07	42.85
17	SACOL0150	Q5HJK7	Cap5O	1	capsular polysaccharide biosynthesis protein Cap5O	4.58	46.8
18	SACOL0151	Q5HJK6	Cap5P	2	UDP-N-acetylglucosamine 2-epimerase Cap5P	5.88	44.31
19	SACOL0157	Q5HJK0	-	1	conserved hypothetical protein	4.4	19.26
20	SACOL0162	Q5HJJ6	-	1	formate dehydrogenase, NAD-dependent	5.93	38.12
21	SACOL0173	Q5HJI5	IpdC	4	indole-3-pyruvate decarboxylase	5.02	60.54
22	SACOL0204	Q5HJF4	PflB	1	formate acetyltransferase	5.15	84.86
23	SACOL0220	Q5HJD8	Hmp	2	flavohemoprotein, putative	5.17	42.91
24	SACOL0222	Q5HJD7	Ldh1	2	L-lactate dehydrogenase	4.69	34.58
25	SACOL0236	Q5HJC5	IspD	1	4-diphosphocytidyl-2C-methyl-D-erythritol synthase, putative	5.95	26.57
26	SACOL0240	Q5HJC1	-	1	4-diphosphocytidyl-2C-methyl-D-erythritol synthase, putative	5.36	26.66
27	SACOL0241	Q5HJC0	-	1	alcohol dehydrogenase, zinc-containing	4.72	38.45
28	SACOL0243	Q5HJB8	-	1	glycosyl transferase, group 2 family protein	7.18	66.21
29	SACOL0257	Q5HJA5	-	1	ribose operon repressor, putative	6.76	36.66
30	SACOL0271	Q5HJ91	-	1	conserved hypothetical protein	4.32	11.04
31	SACOL0279	Q5HJ83	-	2	hypothetical protein	4.34	26.41
32	SACOL0314	Q5HJ51	-	2	conserved hypothetical protein	6.97	30.26
33	SACOL0399	Q5HIW6	-	1	oxidoreductase, putative	5.9	37.75
34	SACOL0427	Q5HIT9	-	1	conserved hypothetical protein	4.83	28.75
35	SACOL0428	Q5HIT8	MetE	2	5-methyltetrahydropteroyltriglutamate--homocysteine methyltransferase	5.41	85.08
36	SACOL0429	Q5HIT7	-	2	5-methyltetrahydrofolate--homocysteine methyltransferase, putative	6.99	68.48
37	SACOL0430	Q5HIT6	-	1	trans-sulfuration enzyme family protein	5.04	42.49
38	SACOL0431	Q5HIT5	-	1	trans-sulfuration enzyme family protein	5.01	41.07
39	SACOL0435	Q5HIT1	-	1	conserved hypothetical protein TIGR00092	4.34	40.59
40	SACOL0437	Q5HIS9	RpsF	1	ribosomal protein S6	4.77	10.82
41	SACOL0438	Q5HIS8	Ssb2	1	single-stranded DNA-binding protein	4.73	18.54

#	locus[1]	AC[2]	protein symbol	protein spots[3]	description	pI	MW [kDa]
42	SACOL0445	Q5HIS2	-	1	conserved domain protein	5.79	10.79
43	SACOL0451	Q5HIR6	AhpF	1	alkyl hydroperoxide reductase, subunit F	4.41	54.72
44	SACOL0452	Q5HIR5	AhpC	1	alkyl hydroperoxide reductase, C subunit	4.66	20.98
45	SACOL0455	Q5HIR2	-	2	conserved hypothetical protein	4.46	35.61
46	SACOL0456	Q5HIR1	-	1	conserved hypothetical protein	6.18	25.8
47	SACOL0457	Q5HIR0	-	1	conserved hypothetical protein	4.62	15.12
48	SACOL0458	Q5HIQ9	Xpt	1	xanthine phosphoribosyltransferase	4.8	20.88
49	SACOL0460	Q5HIQ7	GuaB	1	inosine-5-monophosphate dehydrogenase	5.69	52.85
50	SACOL0461	Q5HIQ6	GuaA	1	GMP synthase	4.76	58.23
51	SACOL0467	Q5HIQ0	-	2	conserved hypothetical protein	5.77	31.83
52	SACOL0495	Q5HIM4	-	1	conserved hypothetical protein	6.15	102.62
53	SACOL0503	Q5HIL6	-	1	trans-sulfuration enzyme family protein	5.67	41.28
54	SACOL0509	Q5HIL0	-	1	MutT/nudix family protein	4.43	15.05
55	SACOL0515	Q5HIK4	GltD	1	glutamate synthase, small subunit	5.04	54.43
56	SACOL0520	Q5HIJ9	DnaX	1	DNA polymerase III, gamma and tau subunits	5.33	63.5
57	SACOL0521	Q5HIJ8	-	2	conserved hypothetical protein TIGR00103	4.74	11.6
58	SACOL0534	Q5HII5	-	1	deoxyribonuclease, TatD family	4.9	29.28
59	SACOL0536	Q5HII3	KsgA	1	dimethyladenosine transferase	5.49	33.74
60	SACOL0540	Q5HIH9	-	1	endoribonuclease L-PSP, putative	6.25	14
61	SACOL0541	Q5HIH8	SpoVG	1	spoVG protein	4.4	11.28
62	SACOL0544	Q5HIH5	PrsA	1	ribose-phosphate pyrophosphokinase	6.63	35.28
63	SACOL0545	Q5HIH4	RplY	2	ribosomal Protein L25	4.08	23.79
64	SACOL0549	Q5HIH0	-	1	tetrapyrrole methylase family protein	4.36	45.11
65	SACOL0554	Q5HIG5	Hpt	1	hypoxanthine phosphoribosyltransferase	4.81	20.15
66	SACOL0556	Q5HIG3	-	1	chaperonin, 33 kDa	4.71	31.82
67	SACOL0557	Q5HIG2	CysK	1	cysteine synthase	5.13	32.98
68	SACOL0562	Q5HIF7	LysS	1	lysyl-tRNA synthetase	4.89	56.72
69	SACOL0565	Q5HIF4	-	2	conserved hypothetical protein	5.94	20.63
70	SACOL0567	Q5HIF2	CtsR	1	transcriptional regulator CtsR	6.27	17.84
71	SACOL0570	None	ClpC	2	ATP-dependent Clp protease, ATP-binding subunit ClpC, authentic frameshift	5.47	91.04
72	SACOL0572	Q5HIE9	RadA	1	DNA repair protein RadA	7.05	49.86
73	SACOL0574	Q5HIE7	GltX	1	glutamyl-tRNA synthetase	5.09	56.3
74	SACOL0576	Q5HIE5	CysS	1	cysteinyl-tRNA synthetase	5.04	53.69
75	SACOL0578	Q5HIE3	-	1	RNA methyltransferase, TrmH family	6.64	27.21
76	SACOL0579	Q5HIE2	-	2	conserved hypothetical protein	5.36	20.25
77	SACOL0582	Q5HID9	NusG	1	transcription antitermination protein NusG	4.79	20.66
78	SACOL0585	Q5HID6	RplJ	2	ribosomal protein L10	4.49	17.71
79	SACOL0586	Q5HID5	RplL	2	ribosomal protein L7/L12	4.32	12.71
80	SACOL0589	Q5HID2	RpoC	1	DNA-directed RNA polymerase, beta subunit	6.94	135.41
81	SACOL0593	Q5HIC8	FusA	1	translation elongation factor G	4.52	76.61
82	SACOL0594	Q5HIC7	Tuf	1	translation elongation factor Tu	4.48	43.1
83	SACOL0596	Q5HIC5	-	1	aminotransferase, class II	5.01	42.89
84	SACOL0597	Q5HIC4	-	1	conserved hypothetical protein	4.66	32.18
85	SACOL0600	Q5HIC1	IlvE	5	branched-chain amino acid aminotransferase	4.63	40.09

#	locus[1]	AC[2]	protein symbol	protein spots[3]	description	pI	MW [kDa]
86	SACOL0602	Q5HIB9	-	2	hydrolase, haloacid dehalogenase-like family	4.35	24.99
87	SACOL0603	Q5HIB8	-	1	deoxynucleoside kinase family protein	4.8	26.23
88	SACOL0607	Q5HIB5	-	1	azoreductase	6.08	20.91
89	SACOL0613	Q5HIA9	-	1	conserved hypothetical protein TIGR00294	4.86	33.48
90	SACOL0614	Q5HIA8	-	2	conserved hypothetical protein	4.89	24.92
91	SACOL0615	Q5HIA7	-	1	conserved hypothetical protein	5.9	13.69
92	SACOL0617	Q5HIA5	-	1	hexulose-6-phosphate synthase, putative	4.32	22.44
93	SACOL0618	Q5HIA4	-	1	SIS domain protein	5.19	19.56
94	SACOL0626	Q5HI96	ThiD1	2	phosphomethylpyrimidine kinase	4.61	29.86
95	SACOL0627	Q5HI95	Ung	1	uracil-DNA glycosylase	6.68	24.94
96	SACOL0633	Q5HI89	-	1	conserved hypothetical protein	4.91	29.39
97	SACOL0634	Q5HI88	Pta	1	phosphate acetyltransferase	4.46	34.95
98	SACOL0637	Q5HI85	MvaD	1	mevalonate diphosphate decarboxylase	4.84	36.82
99	SACOL0638	Q5HI84	-	1	phosphomevalonate kinase	6.51	40.22
100	SACOL0656	Q5HI66	-	1	conserved hypothetical protein	6.26	20.24
101	SACOL0658	Q5HI65	-	1	HD domain protein	5.81	50.6
102	SACOL0660	Q5HI63	-	1	alcohol dehydrogenase, zinc-containing	5.15	36.05
103	SACOL0663	Q5HI60	ArgS	1	arginyl-tRNA synthetase	4.86	62.38
104	SACOL0668	Q5HI55	-	1	hydrolase, alpha/beta hydrolase fold family	5.62	30.92
105	SACOL0721	Q5HI05	-	1	conserved hypothetical protein	4.33	23.74
106	SACOL0731	Q5HHZ6	-	2	transcriptional regulator, LysR family	5.22	33.24
107	SACOL0766	Q5HHW4	SaeR	1	DNA-binding response regulator SaeR	5.05	26.86
108	SACOL0773	Q5HHV7	PabA	1	para-aminobenzoate synthase, glutamine amidotransferase, component II	5.8	22.38
109	SACOL0776	Q5HHV6	-	1	conserved hypothetical protein TIGR00370	6.73	27.12
110	SACOL0785	Q5HHU8	-	2	conserved hypothetical protein	4.71	20.99
111	SACOL0789	Q5HHU4	-	1	GTP cyclohydrolase I family protein	5.24	19.65
112	SACOL0792	Q5HHU0	NrdE	1	ribonucleoside-diphosphate reductase, alpha subunit	5	80.13
113	SACOL0793	Q5HHT9	NrdF	1	ribonucleoside-diphosphate reductase 2, beta subunit	4.52	37.51
114	SACOL0806	Q5HHS7	PepT	1	peptidase T	4.56	45.85
115	SACOL0815	Q5HHR8	-	1	ribosomal subunit interface protein	4.91	22.22
116	SACOL0816	Q5HHR7	SecA	1	preprotein translocase, SecA subunit	4.87	95.96
117	SACOL0821	Q5HHR2	-	1	HD domain protein	4.8	25.7
118	SACOL0824	Q5HHQ9	UvrA	3	excinuclease ABC, A subunit	6.28	105.37
119	SACOL0825	Q5HHQ8	HprK	1	HPr kinase/phosphatase	6.28	34.48
120	SACOL0829	Q5HHQ4	TrxB	1	thioredoxin-disulfide reductase	5	33.62
121	SACOL0830	Q5HHQ3	-	1	conserved hypothetical protein	5.71	34.81
122	SACOL0833	Q5HHQ0	ClpP	1	ATP-dependent Clp protease, proteolytic subunit ClpP	4.9	21.51
123	SACOL0837	Q5HHP6	GapR	2	gap transcriptional regulator	6.11	37.21
124	SACOL0838	Q5HHP5	GapA1	1	glyceraldehyde 3-phosphate dehydrogenase	4.65	36.28
125	SACOL0839	Q5HHP4	Pgk	2	phosphoglycerate kinase	4.96	42.6
126	SACOL0840	Q5HHP3	TpiA	1	triosephosphate isomerase	4.52	27.26
127	SACOL0841	Q5HHP2	Pgm	1	phosphoglycerate mutase, 2,3-bisphosphoglycerate-independent	4.49	56.42
128	SACOL0842	Q5HHP1	Eno	1	enolase	4.28	47.12

#	locus[1]	AC[2]	protein symbol	protein spots[3]	description	pI	MW [kDa]
129	SACOL0845	Q5HHN8	Est	2	carboxylesterase	4.26	28.09
130	SACOL0872	Q5HHL3	-	1	OsmC/Ohr family protein	4.53	15.34
131	SACOL0875	Q5HHL0	-	2	thioredoxin, putative	4.23	12.14
132	SACOL0877	Q5HHK8	GcvH	2	glycine cleavage system H protein	3.7	14.09
133	SACOL0879	Q5HHK7	-	1	conserved hypothetical protein	4.09	33.5
134	SACOL0890	Q5HHJ6	-	1	transcriptional regulator, Cro/CI family	6.55	12.7
135	SACOL0914	Q5HHH2	-	1	FeS assembly ATPase SufC	4.61	28.28
136	SACOL0915	Q5HHH1	SufD	1	FeS assembly protein SufD	5.21	48.55
137	SACOL0916	Q5HHH0	-	1	cysteine desulfurase, SufS subfamily	5.29	46.28
138	SACOL0917	Q5HHG9	-	1	NifU domain protein	4.44	17.02
139	SACOL0918	Q5HHG8	SufB	1	FeS assembly protein SufB	4.81	52.53
140	SACOL0930	Q5HHF7	-	1	conserved hypothetical protein	4.65	16.78
141	SACOL0931	Q5HHF6	-	1	hydrolase, haloacid dehalogenase-like family	4.23	27.95
142	SACOL0932	Q5HHF5	-	1	D-isomer specific 2-hydroxyacid dehydrogenase family protein	4.75	35.36
143	SACOL0935	Q5HHF2	DltA	1	D-alanine-activating enzyme/D-alanine-D-alanyl carrier protein ligase	4.55	54.67
144	SACOL0939	Q5HHE8	-	1	NifU domain protein	4.25	7.97
145	SACOL0944	Q5HHE4	-	1	NADH dehydrogenase, putative	5.15	44.1
146	SACOL0945	Q5HHE3	-	1	cytosol aminopeptidase	5.71	54.13
147	SACOL0957	Q5HHD1	-	2	peptidyl-prolyl cis-trans isomerase, cyclophilin-type	4.29	21.65
148	SACOL0959	Q5HHC9	-	1	NADH-dependent flavin oxidoreductase, Oye family	5.11	42.11
149	SACOL0961	Q5HHC7	GluD	1	glutamate dehydrogenase, NAD-specific	5	45.76
150	SACOL0966	Q5HHC2	Pgi	1	glucose-6-phosphate isomerase	4.55	49.81
151	SACOL0971	Q5HHB7	RexA	1	exonuclease RexA	4.95	141.26
152	SACOL0973	Q5HHB6	-	1	fumarylacetoacetate hydrolase family protein	4.6	33.11
153	SACOL0975	Q5HHB4	-	1	CoA-disulfide reductase	5.05	49.29
154	SACOL0976	Q5HHB3	-	1	hydrolase, haloacid dehalogenase-like family	5.91	31.67
155	SACOL0979	Q5HHB0	ClpB	1	ATP-dependent Clp protease, ATP-binding subunit ClpB	4.68	98.3
156	SACOL0987	Q5HHA2	FabH	1	3-oxoacyl-(acyl-carrier-protein) synthase III	4.64	33.88
157	SACOL0988	Q5HHA1	FabF	4	3-oxoacyl-(acyl-carrier-protein) synthase II	4.82	43.74
158	SACOL1003	Q5HH86	-	3	negative regulator of competence MecA, putative	4.12	28.31
159	SACOL1005	Q5HH84	PepF	1	oligoendopeptidase F	4.92	69.82
160	SACOL1008	Q5HH81	-	1	conserved hypothetical protein	6.14	23.39
161	SACOL1009	Q5HH80	-	1	conserved hypothetical protein	4	13.53
162	SACOL1010	Q5HH79	RelA1	1	GTP pyrophosphokinase	6.16	25.24
163	SACOL1016	Q5HH75	FabI	1	enoyl-(acyl-carrier-protein) reductase	5.71	27.99
164	SACOL1023	O86491	MurE	1	UDP-N-acetylmuramoylalanyl-D-glutamate--2,6-diaminopimelate ligase	5.34	54.1
165	SACOL1034	Q5HH58	-	1	lipoate-protein ligase A family protein	4.92	37.93
166	SACOL1052	Q5HH40	MenD	1	2-succinyl-6-hydroxy-2,4-cyclohexadiene-1-carboxylic acid synthase/2-oxoglutarate decarboxylase	6.24	63.08
167	SACOL1054	Q5HH38	MenB	2	naphthoate synthase	5.24	30.43
168	SACOL1058	Q5HH34	-	2	aminotransferase, class I	5.67	43.15

#	locus[1]	AC[2]	protein symbol	protein spots[3]	description	pI	MW [kDa]
169	SACOL1072	Q5HH21	FolD	1	methylenetetrahydrofolate dehydrogenase/methenyltetrahydrofolate cyclohydrolase	5.25	30.84
170	SACOL1073	Q5HH20	PurE	1	phosphoribosylaminoimidazole carboxylase, catalytic subunit	6.52	17.07
171	SACOL1074	Q5HH19	PurK	1	phosphoribosylaminoimidazole carboxylase, ATPase subunit	5.83	42.49
172	SACOL1075	Q5HH18	PurC	1	phosphoribosylaminoimidazole-succinocarboxamide synthase	5.07	26.69
173	SACOL1077	Q5HH16	PurQ	1	phosphoribosylformylglycinamidine synthase I	4.7	24.53
174	SACOL1079	Q5HH14	PurF	1	amidophosphoribosyltransferase	6.56	54.4
175	SACOL1080	Q5HH13	PurM	3	phosphoribosylformylglycinamidine cyclo-ligase	4.52	37.02
176	SACOL1081	Q5HH12	PurN	1	phosphoribosylglycinamide formyltransferase	5.11	21.15
177	SACOL1082	Q5HH11	PurH	1	phosphoribosylaminoimidazolecarboxamide formyltransferase/IMP cyclohydrolase	5.93	54.35
178	SACOL1083	Q5HH10	PurD	1	phosphoribosylamine--glycine ligase	4.89	45.86
179	SACOL1089	Q5HH04	-	1	conserved hypothetical protein	6.81	44.77
180	SACOL1092	Q5HH01	PtsI	1	phosphoenolpyruvate-protein phosphotransferase	4.39	63.22
181	SACOL1096	Q5HGZ7	-	1	TrkA potassium uptake family protein	5.33	24.28
182	SACOL1100	Q5HGZ3	Def1	1	polypeptide deformylase	6.03	20.56
183	SACOL1102	Q5HGZ1	PdhA	1	pyruvate dehydrogenase complex E1 component, alpha subunit	4.63	41.38
184	SACOL1103	Q5HGZ0	PdhB	1	pyruvate dehydrogenase complex E1 component, beta subunit	4.36	35.22
185	SACOL1104	Q5HGY9	PdhC	1	pyruvate dehydrogenase complex E2 component, dihydrolipoamide acetyltransferase	4.63	46.38
186	SACOL1105	Q5HGY8	PdhD	2	pyruvate dehydrogenase complex E3 component, lipoamide dehydrogenase	4.67	49.45
187	SACOL1108	Q5HGY5	-	1	spermidine/putrescine ABC transporter, ATP-binding protein	4.37	41.33
188	SACOL1118	Q5HGX5	TypA	1	GTP-binding protein TypA	4.69	69.17
189	SACOL1120	Q5HGX3	-	1	conserved hypothetical protein	5.03	18.58
190	SACOL1123	Q5HGX0	Pyc	1	pyruvate carboxylase	4.93	128.55
191	SACOL1134	Q5HGV9	CoaD	3	phosphopantetheine adenylyltransferase	6.68	18.37
192	SACOL1148	Q5HGU6	PheS	3	phenylalanyl-tRNA synthetase, alpha subunit	5.48	40.11
193	SACOL1149	Q5HGU5	PheT	3	phenylalanyl-tRNA synthetase, beta subunit	4.42	88.92
194	SACOL1155	Q5HGT9	TrxA	1	thioredoxin	4.14	11.44
195	SACOL1159	Q5HGT5	SdhA	1	succinate dehydrogenase, flavoprotein subunit	5.32	65.5
196	SACOL1163	Q5HGT1	-	1	conserved hypothetical protein TIGR00040	5.2	19.26
197	SACOL1189	Q5HGQ5	-	1	acetyltransferase, GNAT family	4.61	17
198	SACOL1191	Q5HGQ3	MraZ	1	mraZ protein	4.52	17.24
199	SACOL1192	Q5HGQ2	-	1	S-adenosyl-methyltransferase MraW	5.98	35.71
200	SACOL1196	Q5HGP8	MurD	1	UDP-N-acetylmuramoylalanine--D-glutamate ligase	5.49	49.84
201	SACOL1198	Q5HGP6	FtsA	1	cell division protein FtsA	4.22	52.93
202	SACOL1199	Q5HGP5	FtsZ	1	cell division protein FtsZ	4.6	41.04
203	SACOL1200	Q5HGP4	-	1	conserved hypothetical protein TIGR00726	6.23	30.2
204	SACOL1201	Q5HGP3	-	2	conserved hypothetical protein TIGR00044	5.73	25.6
205	SACOL1204	Q5HGP0	YlmH	1	YlmH protein	6.52	30.06

#	locus[1]	AC[2]	protein symbol	protein spots[3]	description	pI	MW [kDa]
206	SACOL1205	Q5HGN9	-	1	cell-division initiation protein, putative	4.64	23.51
207	SACOL1206	Q5HGN8	IleS	2	isoleucyl-tRNA synthetase	5.13	104.89
208	SACOL1209	Q5HGN5	-	1	ribosomal large subunit pseudouridine synthases, RluD subfamily	6.01	34.6
209	SACOL1210	Q5HGN4	PyrR	1	pyrimidine operon regulatory protein	5.09	19.85
210	SACOL1212	Q5HGN2	PyrB	1	aspartate carbamoyltransferase	5.76	33.26
211	SACOL1213	Q5HGN1	PyrC	1	dihydroorotase	4.85	46.37
212	SACOL1214	Q5HGN0	CarA	1	carbamoyl-phosphate synthase, small subunit	6.19	40.39
213	SACOL1215	Q5HGM9	CarB	1	carbamoyl-phosphate synthase, large subunit	4.6	117.19
214	SACOL1216	Q5HGM8	PyrF	1	orotidine 5-phosphate decarboxylase	6.1	25.6
215	SACOL1217	Q5HGM7	PyrE	1	orotate phosphoribosyltransferase	4.79	22.04
216	SACOL1219	Q5HGM5	-	1	conserved hypothetical protein	4.14	14.86
217	SACOL1221	Q5HGM3	Gmk	1	guanylate kinase	5.1	24.04
218	SACOL1223	Q5HGM1	CoaBC	5	phosphopantothenoylcysteine decarboxylase/phosphopantothenate--cysteine ligase	5.87	44.4
219	SACOL1224	Q5HGM0	PriA	1	primosomal protein N`	6.74	92.52
220	SACOL1229	Q5HGL5	Sun	1	sun protein	6.06	50.09
221	SACOL1236	Q5HGL0	-	1	thiamine pyrophosphokinase	6.08	23.88
222	SACOL1239	Q5HGK8	-	1	conserved hypothetical protein	5.87	13.39
223	SACOL1243	Q5HGK4	PlsX	1	fatty acid/phospholipid synthesis protein PlsX	6.27	35.43
224	SACOL1245	Q5HGK2	FabG1	1	3-oxoacyl-(acyl-carrier-protein) reductase	5.1	25.89
225	SACOL1270	Q5HGH9	HslV	1	heat shock protein HslVU, ATPase subunit HslV	6.12	19.57
226	SACOL1271	Q5HGH8	HslU	1	heat shock protein HslVU, ATPase subunit HslU	4.95	52.31
227	SACOL1272	Q5HGH7	CodY	1	transcriptional regulator CodY	6.08	28.76
228	SACOL1274	Q5HGH6	RpsB	2	ribosomal protein S2	5.06	29.4
229	SACOL1276	Q5HGH4	Tsf	1	translation elongation factor Ts	4.77	32.49
230	SACOL1277	Q5HGH3	PyrH	1	uridylate kinase	6.29	26.15
231	SACOL1282	Q5HGG8	ProS	1	prolyl-tRNA synthetase	4.85	63.83
232	SACOL1285	Q5HGG5	-	1	N utilization substance protein A, putative	4.3	43.74
233	SACOL1288	Q5HGG2	InfB	2	translation initiation factor IF-2	4.84	77.87
234	SACOL1291	Q5HGF9	RibF	1	riboflavin biosynthesis protein RibF	5.89	36.69
235	SACOL1292	Q5HGF8	RpsO	1	ribosomal protein S15	11.12	10.61
236	SACOL1293	Q5HGF7	Pnp	1	polyribonucleotide nucleotidyltransferase	4.61	77.36
237	SACOL1294	Q5HGF6	-	1	metallo-beta-lactamase family protein	6.1	58.79
238	SACOL1296	Q5HGF4	-	1	transcriptional regulator, GntR family, putative	4.34	26.95
239	SACOL1304	Q5HGE6	-	2	recA protein	4.85	37.66
240	SACOL1307	Q5HGE3	-	1	conserved hypothetical protein TIGR00282	6.61	29.88
241	SACOL1308	Q5HGE2	-	1	pyruvate ferredoxin oxidoreductase, alpha subunit	5.06	64.56
242	SACOL1316	Q5HGD5	HexB	2	DNA mismatch repair protein HexB	5.05	76.85
243	SACOL1320	Q5HGD2	GlpK	1	glycerol kinase	4.67	55.63
244	SACOL1321	Q5HGD1	GlpD	1	aerobic glycerol-3-phosphate dehydrogenase	7.04	62.39
245	SACOL1323	Q5HGC9	MiaA	1	tRNA delta(2)-isopentenylpyrophosphate transferase	6.81	35.87
246	SACOL1325	Q5HGC7	GpxA1	1	glutathione peroxidase	6.78	18.12
247	SACOL1329	Q5HGC3	FemC	1	glutamine synthetase FemC	4.84	50.84

#	locus[1]	AC[2]	protein symbol	protein spots[3]	description	pI	MW [kDa]
248	SACOL1349	Q5HGA5	-	1	conserved hypothetical protein	6.19	38.63
249	SACOL1360	Q5HG94	-	2	aspartate kinase	6.39	52
250	SACOL1362	Q5HG92	Hom	2	homoserine dehydrogenase	5.04	46.87
251	SACOL1363	Q5HG91	ThrC	1	threonine synthase	6.18	37.87
252	SACOL1364	Q5HG90	ThrB	1	homoserine kinase	5.15	33.27
253	SACOL1365	Q5HG89	-	1	hydrolase, haloacid dehalogenase-like family	4.33	29.82
254	SACOL1366	Q5HG88	-	2	conserved hypothetical protein	6.52	12.28
255	SACOL1368	Q5HG86	KatA	1	catalase	4.98	54.94
256	SACOL1371	Q5HG83	GuaC	1	GMP reductase	7.06	36.12
257	SACOL1377	Q5HG77	Tkt	1	transketolase	4.71	72.25
258	SACOL1385	Q5HG69	AcnA	1	aconitate hydratase	4.59	98.97
259	SACOL1386	Q5HG68	-	1	conserved hypothetical protein TIGR00051	5.35	18.18
260	SACOL1387	Q5HG67	-	3	conserved hypothetical protein	3.88	11.5
261	SACOL1390	Q5HG64	ParC	1	DNA topoisomerase IV, A subunit	6.37	91
262	SACOL1402	Q5HG53	-	1	glutamyl aminopeptidase, putative	5.25	37.85
263	SACOL1411	Q5HG44	-	1	femB protein	5.38	49.68
264	SACOL1413	Q5HG42	-	2	hypothetical protein	4.4	18.46
265	SACOL1419	Q5HG36	-	1	oligoendopeptidase F, putative	4.39	69.3
266	SACOL1427	Q5HG28	-	3	ABC transporter, ATP-binding protein	4.58	60.26
267	SACOL1429	Q5HG26	Asd	1	aspartate-semialdehyde dehydrogenase	4.65	36.28
268	SACOL1430	Q5HG25	DapA	4	dihydrodipicolinate synthase	4.54	32.58
269	SACOL1431	Q5HG24	DapB	1	dihydrodipicolinate reductase	5.14	26.65
270	SACOL1432	Q5HG23	DapD	1	2,3,4,5-tetrahydropyridine-2,6-dicarboxylate N-succinyltransferase	4.44	25.26
271	SACOL1433	Q5HG22	-	3	peptidase, M20/M25/M40 family	5.35	43.16
272	SACOL1434	Q5HG21	-	2	alanine racemase family protein	6.43	41.41
273	SACOL1435	Q5HG20	LysA	1	diaminopimelate decarboxylase	5.66	47.03
274	SACOL1437	Q5HG18	-	1	cold shock protein, CSD family	4.23	7.32
275	SACOL1439	Q5HG16	-	1	acylphosphatase	4.71	10.16
276	SACOL1441	Q5HG14	-	1	tellurite resistance protein, putative	5.96	43.41
277	SACOL1445	Q5HG10	-	1	CbbQ/NirQ/NorQ/GpvN family protein	4.69	29.45
278	SACOL1447	Q5HG08	-	2	conserved hypothetical protein	4.08	30.08
279	SACOL1448	Q5HG07	SucB	1	2-oxoglutarate dehydrogenase, E2 component, dihydroipoamide succinyltransferase	4.57	46.67
280	SACOL1449	Q5HG06	SucA	2	2-oxoglutarate dehydrogenase, E1 component	5.4	105.34
281	SACOL1457	Q5HFZ9	-	2	PTS system, IIA component	4.27	17.96
282	SACOL1460	Q5HFZ8	-	2	degV family protein	5.78	30.64
283	SACOL1461	Q5HFZ7	FolA	1	dihydrofolate reductase	6.3	18.25
284	SACOL1462	Q5HFZ6	ThyA	1	thymidylate synthase	5.37	36.84
285	SACOL1464	Q5HFZ5	-	1	conserved hypothetical protein	4.15	16.01
286	SACOL1483	Q5HFX9	-	3	conserved hypothetical protein	4.79	43.33
287	SACOL1503	Q5HFW0	-	6	TPR domain protein	3.79	48.42
288	SACOL1505	Q5HFV8	AroB	1	3-dehydroquinate synthase	6.09	40.32
289	SACOL1506	Q5HFV7	AroC	3	chorismate synthase	5.72	43.05
290	SACOL1509	Q5HFV4	-	2	nucleoside diphosphate kinase	4.82	16.57

#	locus[1]	AC[2]	protein symbol	protein spots[3]	description	pI	MW [kDa]
291	SACOL1514	Q5HFU9	GpsA	1	glycerol-3-phosphate dehydrogenase, NAD-dependent	6.26	36.07
292	SACOL1515	Q5HFU8	-	1	GTP-binding protein, Era/TrmE family	5	48.98
293	SACOL1516	Q5HFU7	RpsA	1	ribosomal protein S1	4.24	43.29
294	SACOL1519	Q5HFU5	AnsA	2	L-asparaginase	6.37	35.58
295	SACOL1520	Q5HFU4	-	2	pyridine nucleotide-disulfide oxidoreductase	5.4	36.74
296	SACOL1535	Q5HFT0	SrrA	1	DNA-binding response regulator SrrA	5.03	28.16
297	SACOL1541	Q5HFS4	-	1	transcriptional regulator, Fur family	6.16	17.24
298	SACOL1543	Q5HFS2	-	3	oxidoreductase, aldo/keto reductase family	5.08	33.51
299	SACOL1546	Q5HFR9	ProC	1	pyrroline-5-carboxylate reductase	4.77	29.84
300	SACOL1549	Q5HFR7	Zwf	1	glucose-6-phosphate 1-dehydrogenase	5.19	56.97
301	SACOL1553	Q5HFR3	-	1	glyoxalase family protein	4.12	13.89
302	SACOL1554	Q5HFR2	Gnd	1	6-phosphogluconate dehydrogenase, decarboxylating	4.75	51.8
303	SACOL1555	Q5HFR1	-	1	peptidase, M20/M25/M40 family	4.8	40.24
304	SACOL1560	Q5HFQ6	-	1	2-oxoisovalerate dehydrogenase, E2 component, dihydrolipoamide acetyltransferase	5.47	46.74
305	SACOL1561	Q5HFQ5	-	1	2-oxoisovalerate dehydrogenase, E1 component, beta subunit	4.52	36.06
306	SACOL1562	Q5HFQ4	-	1	2-oxoisovalerate dehydrogenase, E1 component, alpha subunit	4.95	36.23
307	SACOL1564	Q5HFQ2	RecN	1	DNA repair protein RecN	4.73	64.3
308	SACOL1565	Q5HFQ1	ArgR	1	arginine repressor	5.28	17.1
309	SACOL1566	Q5HFQ0	IspA	5	geranyltranstransferase	4.49	32.81
310	SACOL1569	Q5HFP7	NusB	1	N utilization substance protein B	4.81	15.06
311	SACOL1571	Q5HFP5	AccC	2	acetyl-CoA carboxylase, biotin carboxylase	5.15	50.05
312	SACOL1572	Q5HFP4	AccB	1	acetyl-CoA carboxylase, biotin carboxyl carrier protein	4.23	17.12
313	SACOL1587	Q5HFN0	Efp	1	translation elongation factor P	4.46	20.55
314	SACOL1588	Q5HFM9	-	1	proline dipeptidase	5.06	39.34
315	SACOL1591	Q5HFM6	-	1	lipoate-protein ligase A family protein	5.15	31.88
316	SACOL1593	Q5HFM4	-	1	glycine cleavage system P protein, subunit 2	5.92	54.78
317	SACOL1604	Q5HFL3	Glk	1	glucokinase	5.05	35.08
318	SACOL1610	Q5HFK7	SodA2	1	superoxide dismutase	4.86	22.71
319	SACOL1611	Q5HFK6	-	1	transcriptional regulator, Fur family	6.07	15.92
320	SACOL1614	Q5HFK3	Nfo	3	endonuclease IV	5.9	33.16
321	SACOL1618	Q5HFJ9	RpoD	1	RNA polymerase sigma factor RpoD	4.73	42.17
322	SACOL1620	Q5HFJ7	-	1	conserved hypothetical protein	4.93	30.78
323	SACOL1622	Q5HFJ5	GlyS	2	glycyl-tRNA synthetase	4.74	53.62
324	SACOL1624	Q5HFJ3	Era	1	GTP-binding protein Era	6.52	34.33
325	SACOL1627	Q5HFJ0	-	1	conserved hypothetical protein TIGR00043	4.08	17.95
326	SACOL1630	Q5HFI7	-	2	conserved hypothetical protein	5.68	35.18
327	SACOL1636	Q5HFI1	DnaJ	2	dnaJ protein	5.79	41.76
328	SACOL1637	Q5HFI0	DnaK	1	dnaK protein	4.39	66.36
329	SACOL1638	Q5HFH9	GrpE	1	heat shock protein GrpE	4.2	24.01
330	SACOL1648	Q5HFG9	-	1	iojap-related protein	4.43	13.48
331	SACOL1649	Q5HFG8	-	2	conserved hypothetical protein TIGR00488	5.15	22.46
332	SACOL1651	Q5HFG6	-	1	conserved hypothetical protein TIGR00253	5.66	11.05

#	locus[1]	AC[2]	protein symbol	protein spots[3]	description	pI	MW [kDa]
333	SACOL1652	Q5HFG5	AroE	1	shikimate 5-dehydrogenase	5.61	29.88
334	SACOL1655	Q5HFG2	Mtn	2	5-methylthioadenosine/S-adenosylhomocysteine nucleosidase	4.56	24.53
335	SACOL1661	Q5HFF6	-	2	acetyl-CoA carboxylase, biotin carboxylase, putative	6.37	50.22
336	SACOL1665	Q5HFF2	GreA	1	transcription elongation factor GreA	4.24	17.74
337	SACOL1666	Q5HFF1	Udk	1	uridine kinase	6.04	23.5
338	SACOL1669	Q5HFE8	-	1	O-methyltransferase family protein	5.32	24.53
339	SACOL1670	Q5HFE7	-	2	conserved hypothetical protein	3.57	11.95
340	SACOL1672	Q5HFE5	-	1	conserved hypothetical protein	4.5	10.3
341	SACOL1673	Q5HFE4	AlaS	1	alanyl-tRNA synthetase	4.75	98.52
342	SACOL1676	Q5HFE1	TrmU	2	tRNA (5-methylaminomethyl-2-thiouridylate)-methyltransferase	5.16	42.15
343	SACOL1677	Q5HFE0	-	3	aminotransferase, class V	4.53	42.48
344	SACOL1681	Q5HFD6	-	1	Rrf2 family protein	4.79	15.64
345	SACOL1685	Q5HFD3	AspS	1	aspartyl-tRNA synthetase	4.69	66.6
346	SACOL1688	Q5HFD0	-	1	conserved hypothetical protein TIGR00256	7.34	16.7
347	SACOL1695	Q5HFC3	QueA	2	S-adenosylmethionine:tRNA ribosyltransferase-isomerase	5.3	38.97
348	SACOL1697	Q5HFC1	RuvA	3	Holliday junction DNA helicase RuvA	6.03	22.26
349	SACOL1710	Q5HFA8	ValS	2	valyl-tRNA synthetase	4.74	101.72
350	SACOL1714	Q5HFA5	HemL1	1	glutamate-1-semialdehyde-2,1-aminomutase	5.3	46.39
351	SACOL1722	Q5HF97	Tig	2	trigger factor	4.06	48.61
352	SACOL1724	Q5HF95	-	1	MutT/nudix family protein	4.2	22.95
353	SACOL1729	Q5HF90	ThrS	1	threonyl-tRNA synthetase	5.04	74.46
354	SACOL1735	Q5HF85	CoaE	4	dephospho-CoA kinase	4.47	23.62
355	SACOL1737	Q5HF83	PolA	3	DNA polymerase I	4.77	99.19
356	SACOL1741	Q5HF79	Icd	1	isocitrate dehydrogenase, NADP-dependent	4.57	46.42
357	SACOL1742	Q5HF78	GltA	1	citrate synthase	5.34	42.59
358	SACOL1745	Q5HF76	Pyk	1	pyruvate kinase	5	63.1
359	SACOL1746	Q5HF75	PfkA	2	6-phosphofructokinase	5.71	34.84
360	SACOL1747	Q5HF74	AccA	2	acetyl-CoA carboxylase, carboxyl transferase, alpha subunit	4.96	35.07
361	SACOL1749	Q5HF72	-	1	NADP-dependent malic enzyme, putative	4.82	44.23
362	SACOL1751	Q5HF70	-	1	DHH subfamily 1 protein	4.47	35.1
363	SACOL1756	Q5HF67	PepQ	3	proline dipeptidase	4.73	39.55
364	SACOL1759	Q5HF64	-	1	universal stress protein family	5.68	18.47
365	SACOL1760	Q5HF63	AckA	2	acetate kinase	5.89	44.04
366	SACOL1762	Q5HF61	-	1	thiol peroxidase, putative	4.34	18.01
367	SACOL1768	Q5HF55	-	2	GAF domain protein	4.67	17.11
368	SACOL1769	Q5HF54	RpsD	1	ribosomal protein S4	10.56	23.01
369	SACOL1772	Q5HF51	-	1	aminotransferase, class V	7.24	42.85
370	SACOL1773	Q5HF50	SerA	2	D-3-phosphoglycerate dehydrogenase	5.31	57.62
371	SACOL1778	Q5HF45	TyrS	1	tyrosyl-tRNA synthetase	5.05	47.58
372	SACOL1782	Q5HF42	Fhs	2	formate--tetrahydrofolate ligase	6.81	61.25
373	SACOL1786	Q5HF38	CcpA	1	catabolite control protein A	5.51	36.06
374	SACOL1787	Q5HF37	-	4	chorismate mutase/phospho-2-dehydro-3-deoxyheptonate aldolase	6.03	40.62
375	SACOL1790	Q5HF34	MurC	1	UDP-N-acetylmuramate--alanine ligase	4.77	49.19

#	locus[1]	AC[2]	protein symbol	protein spots[3]	description	pI	MW [kDa]
376	SACOL1792	Q5HF32	-	2	conserved hypothetical protein	4.46	21.69
377	SACOL1793	Q5HF31	-	1	conserved hypothetical protein	6.38	33.07
378	SACOL1794	Q5HF30	-	1	thioredoxin, putative	4.14	11.86
379	SACOL1795	Q5HF29	PepA1	1	glutamyl aminopeptidase	5.15	39.78
380	SACOL1800	Q5HF24	Dat	1	D-alanine aminotransferase	4.68	31.89
381	SACOL1801	Q5HF23	-	1	peptidase, M20/M25/M40 family	4.32	52.82
382	SACOL1808	Q5HF16	LeuS	1	leucyl-tRNA synthetase	4.79	91.79
383	SACOL1817	Q5HF08	RibH	2	6,7-dimethyl-8-ribityllumazine synthase	5.78	16.4
384	SACOL1820	Q5HF05	RibD	1	riboflavin biosynthesis protein RibD	6.44	38.95
385	SACOL1831	Q5HEZ4	Tal	1	transaldolase	4.45	25.71
386	SACOL1837	Q5HEY9	MetK	1	S-adenosylmethionine synthetase	4.62	43.64
387	SACOL1889	Q5HEU2	HemE	1	uroporphyrinogen decarboxylase	6.32	39.35
388	SACOL1891	Q5HEU0	-	1	RNAIII-activating protein TRAP	6.6	19.55
389	SACOL1894	Q5HET7	-	1	HIT family protein	4.66	15.94
390	SACOL1895	Q5HET6	-	1	conserved hypothetical protein	5.92	13.21
391	SACOL1898	Q5HET3	Cbf1	1	cmp-binding-factor 1	6.57	35.78
392	SACOL1902	Q5HET0	-	1	conserved hypothetical protein	4.04	13.31
393	SACOL1908	Q5HES4	FumC	1	fumarate hydratase, class II	4.98	51.11
394	SACOL1912	Q5HES0	-	1	glucosamine-6-phosphate isomerase, putative	5.15	22.34
395	SACOL1919	Q5HER3	-	1	transcriptional regulator, Fur family	5.47	17.18
396	SACOL1920	Q5HER2	-	1	D-isomer specific 2-hydroxyacid dehydrogenase family protein	5.69	35.95
397	SACOL1921	Q5HER1	Bcp	1	bacterioferritin comigratory protein	5.34	17.26
398	SACOL1922	Q5HER0	HemL2	1	glutamate-1-semialdehyde-2,1-aminomutase	4.91	46.76
399	SACOL1931	Q5HEQ1	-	1	FAD-binding RecX family protein	5.93	32.24
400	SACOL1933	Q5HEP9	-	1	ThiJ/PfpI family protein	4.36	18.63
401	SACOL1936	Q5HEP6	-	1	cytosolic long-chain acyl-CoA thioester hydrolase family protein	6.63	19.99
402	SACOL1937	Q5HEP5	PepS	1	aminopeptidase PepS	4.59	47.12
403	SACOL1942	Q5HEP0	VraR	1	DNA-binding response regulator VraR	5.39	23.55
404	SACOL1952	Q5HEN0	-	1	ferritins family protein	4.36	19.59
405	SACOL1965	Q5HEL8	LigA	2	DNA ligase, NAD-dependent	4.94	75.08
406	SACOL1968	Q5HEL5	-	1	conserved hypothetical protein	4.27	11.52
407	SACOL1969	Q5HEL4	PurB	1	adenylosuccinate lyase	5.67	49.6
408	SACOL1974	Q5HEK9	NadE	1	NH(3)-dependent NAD+ synthetase	5	30.7
409	SACOL1975	Q5HEK8	-	1	conserved hypothetical protein	5.51	55.89
410	SACOL1982	Q5HEK1	PpaC	1	manganese-dependent inorganic pyrophosphatase	4.45	34.07
411	SACOL1984	Q5HEJ9	AldA2	1	aldehyde dehydrogenase	7.13	51.74
412	SACOL1985	Q5HEJ8	-	3	conserved hypothetical protein	4.84	38.55
413	SACOL1992	Q5HEJ1	-	4	conserved hypothetical protein	4.96	21.92
414	SACOL2000	Q5HEI3	-	2	aminotransferase, putative	4.88	48.15
415	SACOL2016	Q5HEH2	GroEL	1	chaperonin, 60 kDa	4.27	57.63
416	SACOL2017	Q5HEH1	GroES	1	chaperonin, 10 kDa	4.57	10.42
417	SACOL2020	Q5HEG8	-	4	conserved hypothetical protein	5.12	24
418	SACOL2028	Q5HEG1	-	3	fructokinase, putative	4.39	34.99
419	SACOL2029	Q5HEG0	CscA	2	sucrose-6-phosphate hydrolase	4.97	57.82

#	locus[1]	AC[2]	protein symbol	protein spots[3]	description	pI	MW [kDa]
420	SACOL2035	Q5HEF5	-	1	conserved hypothetical protein	5.12	23.6
421	SACOL2038	Q5HEF2	-	2	metalloendopeptidase, putative, glycoprotease family	4.8	36.82
422	SACOL2042	Q5HEE8	IlvD	3	dihydroxy-acid dehydratase	6.35	60.12
423	SACOL2043	Q5HEE7	IlvB	2	acetolactate synthase, large subunit, biosynthetic type	5.02	64.49
424	SACOL2044	Q5HEE6	-	1	acetolactate synthase, small subunit, truncation	4.96	9.62
425	SACOL2045	Q5HEE5	IlvC	1	ketol-acid reductoisomerase	5.04	36.96
426	SACOL2046	Q5HEE4	LeuA	1	2-isopropylmalate synthase	5.76	55.67
427	SACOL2047	Q5HEE3	LeuB	1	3-isopropylmalate dehydrogenase	5.24	38.21
428	SACOL2048	Q5HEE2	LeuC	4	3-isopropylmalate dehydratase, large subunit	5.57	50.33
429	SACOL2049	Q5HEE1	LeuD	2	3-isopropylmalate dehydratase, small subunit	5.63	21.58
430	SACOL2050	Q5HEE0	IlvA2	1	threonine dehydratase	5.46	46.94
431	SACOL2053	Q5HED8	-	3	S1 RNA binding domain protein	6.25	80.93
432	SACOL2054	Q5HED7	RpoF	1	RNA polymerase sigma-37 factor	5.65	29.44
433	SACOL2055	Q5HED6	RsbW	1	anti-sigma B factor	4.45	17.92
434	SACOL2056	P60071	RsbV	1	anti-anti-sigma factor RsbV	4.04	12.2
435	SACOL2057	Q5HED4	RsbU	1	sigma factor B regulator protein	5.44	38.43
436	SACOL2074	Q5HEB7	-	2	D-alanine--D-alanine ligase	4.63	40.23
437	SACOL2091	Q5HEA1	FabZ	1	beta-hydroxyacyl-(acyl-carrier-protein) dehydratase FabZ	5.78	16.08
438	SACOL2092	Q5HEA0	MurAA	1	UDP-N-acetylglucosamine 1-carboxyvinyltransferase 1	5.78	45
439	SACOL2094	Q5HE98	AtpC	1	ATP synthase F1, epsilon subunit	5.66	14.84
440	SACOL2095	Q5HE97	AtpD	7	ATP synthase F1, beta subunit	4.42	51.4
441	SACOL2096	Q5HE96	AtpG	1	ATP synthase F1, gamma subunit	5.93	32.11
442	SACOL2097	Q5HE95	AtpA	1	ATP synthase F1, alpha subunit	4.59	54.57
443	SACOL2098	Q5HE94	AtpH	2	ATP synthase F1, delta subunit	6.55	20.5
444	SACOL2104	Q5HE88	Upp	1	uracil phosphoribosyltransferase	6.52	23.05
445	SACOL2105	Q5HE87	GlyA	1	serine hydroxymethyltransferase	5.99	45.17
446	SACOL2106	Q5HE86	-	1	conserved hypothetical protein TIGR01440	6.23	18.89
447	SACOL2110	Q5HE82	PrfA	1	peptide chain release factor 1	4.54	40.35
448	SACOL2114	Q5HE78	-	2	aldehyde dehydrogenase	4.77	51.97
449	SACOL2116	Q5HE76	MurAB	1	UDP-N-acetylglucosamine 1-carboxyvinyltransferase 2	5.46	45.07
450	SACOL2117	Q5HE75	FbaA	3	fructose-bisphosphate aldolase, class II	4.75	30.84
451	SACOL2119	Q5HE73	PyrG	1	CTP synthase	4.76	59.99
452	SACOL2125	Q5HE67	-	2	peptidase, M20/M25/M40 family	5.27	42.96
453	SACOL2126	Q5HE66	LuxS	1	autoinducer-2 production protein LuxS	5.46	17.51
454	SACOL2130	Q5HE62	DeoD2	1	purine nucleoside phosphorylase	4.57	25.91
455	SACOL2131	Q5HE61	-	1	Dps family protein	4.29	16.69
456	SACOL2133	Q5HE59	-	1	conserved hypothetical protein	6.04	52.95
457	SACOL2135	Q5HE57	ManA1	2	mannose-6-phosphate isomerase, class I	4.33	35.64
458	SACOL2136	Q5HE56	-	1	conserved hypothetical protein	4.7	24.03
459	SACOL2145	Q5HE49	GlmS	1	glucosamine--fructose-6-phosphate aminotransferase (isomerizing)	4.67	65.85
460	SACOL2148	Q5HE46	-	1	PTS system, mannitol-specific IIA component	4.1	15.54
461	SACOL2149	Q9RL68	MtlD	1	mannitol-1-phosphate 5-dehydrogenase	4.66	40.95

#	locus[1]	AC[2]	protein symbol	protein spots[3]	description	pI	MW [kDa]
462	SACOL2151	Q5HE43	GlmM	1	phosphoglucosamine mutase GlmM	4.38	49.27
463	SACOL2156	Q5HE39	-	1	ATP-binding protein, Mrp/Nbp35 family	5.07	38.36
464	SACOL2161	Q5HE34	-	2	UTP-glucose-1-phosphate uridylyltransferase family protein	4.6	44.89
465	SACOL2163	Q5HE32	-	1	conserved hypothetical protein	6.53	10.01
466	SACOL2171	Q5HE24	-	2	aerobactin biosynthesis protein, IucA/IucC family	6.1	76.05
467	SACOL2173	Q5HE23	Asp23	1	alkaline shock protein 23	4.84	19.19
468	SACOL2192	Q5HE04	-	1	oxidoreductase, aldo/keto reductase family	4.38	32.2
469	SACOL2196	Q5HE00	-	2	conserved hypothetical protein	5.22	31.84
470	SACOL2213	Q5HDY4	RpoA	1	DNA-directed RNA polymerase, alpha subunit	4.39	35.01
471	SACOL2218	Q5HDX9	Adk	1	adenylate kinase	4.55	23.97
472	SACOL2220	Q5HDX7	RplO	1	ribosomal protein L15	11.03	15.6
473	SACOL2238	Q5HDV9	RplD	2	ribosomal protein L4	10.6	22.46
474	SACOL2264	Q5HDT6	MoaE	1	molybdenum cofactor biosynthesis protein E	5.35	17.34
475	SACOL2266	Q5HDT4	-	3	molybdopterin biosynthesis MoeA protein, putative	5.51	45.02
476	SACOL2268	Q5HDT2	MoaB	1	molybdenum cofactor biosynthesis protein B	5.68	18.5
477	SACOL2283	Q5HDR7	UreE	2	urease accessory protein UreE	5.24	17.34
478	SACOL2293	Q5HDQ7	-	1	NAD/NADP octopine/nopaline dehydrogenase family protein	4.57	40.74
479	SACOL2296	Q5HDQ4	-	1	D-isomer specific 2-hydroxyacid dehydrogenase family protein	4.91	34.67
480	SACOL2301	Q5HDP9	-	2	formate dehydrogenase, alpha subunit, putative	4.76	111.24
481	SACOL2313	Q5HDN7	-	2	hydrolase, haloacid dehalogenase-like family	4.6	23.57
482	SACOL2321	Q5HDM9	-	4	oxidoreductase, short chain dehydrogenase/reductase family	4.39	31.73
483	SACOL2327	Q5HDM3	HutG	2	formiminoglutamase	5.36	34.51
484	SACOL2332	Q5HDL8	GalM	1	aldose 1-epimerase	4.83	39.28
485	SACOL2335	Q5HDL5	-	1	ABC transporter, ATP-binding protein	6.61	33.72
486	SACOL2344	Q5HDK7	-	1	hypothetical protein	4.93	16.31
487	SACOL2362	Q5HDJ0	Mqo1	1	malate:quinone oxidoreductase	6.69	54.81
488	SACOL2367	Q5HDI5	-	1	quinone oxidoreductase, YhdH/YhfP family	5.65	36.57
489	SACOL2379	Q5HDH4	-	1	conserved hypothetical protein	4.6	15.89
490	SACOL2385	Q5HDG8	-	1	heat shock protein, Hsp20 family	4.22	16.31
491	SACOL2400	Q5HDF4	-	2	acetyltransferase, GNAT family	4.57	20.43
492	SACOL2415	Q5HDD9	Gpm	1	phosphoglycerate mutase	5.05	26.68
493	SACOL2453	Q5HDA5	-	1	amino acid ABC transporter, ATP-binding protein	6.15	46.17
494	SACOL2459	Q5HDA0	PnbA	1	para-nitrobenzyl esterase	6.6	52
495	SACOL2484	Q5HD77	-	1	alkylhydroperoxidase, AhpD family	5.66	16.48
496	SACOL2488	Q5HD73	-	2	oxidoreductase, short-chain dehydrogenase/reductase family	4.91	24.6
497	SACOL2499	Q5HD63	-	1	helicase, putative	6.08	109.9
498	SACOL2501	Q5HD61	-	1	phosphoglucomutase/phosphomannomutase family protein	6.55	68.35
499	SACOL2518	Q5HD45	-	1	conserved hypothetical protein	5.78	27.17
500	SACOL2519	Q5HD44	-	1	conserved hypothetical protein	4.75	19.01
501	SACOL2532	Q5HD32	-	1	acetyltransferase, GNAT family	4.55	10.55
502	SACOL2534	Q5HD30	Frp	1	NAD(P)H-flavin oxidoreductase	5.62	25.39

#	locus[1]	AC[2]	protein symbol	protein spots[3]	description	pI	MW [kDa]
503	SACOL2535	Q5HD29	-	1	D-isomer specific 2-hydroxyacid dehydrogenase family protein	4.9	36.68
504	SACOL2563	Q5HD02	-	1	ATP-dependent Clp protease, putative	4.63	77.84
505	SACOL2574	Q5HCZ1	-	6	D-isomer specific 2-hydroxyacid dehydrogenase family protein	5.26	37.22
506	SACOL2575	Q5HCZ0	-	1	aminotransferase, class I	5.14	42.73
507	SACOL2596	Q5HCX0	-	1	conserved hypothetical protein	4.7	38.19
508	SACOL2597	Q5HCW9	-	1	hydrolase, alpha/beta hydrolase fold family	4.44	31.01
509	SACOL2609	Q5HCV8	-	1	conserved hypothetical protein	4.22	16.94
510	SACOL2615	Q5HCV3	PanB	1	3-methyl-2-oxobutanoate hydroxymethyltransferase	5.58	29.26
511	SACOL2616	Q5HCV2	-	1	2-dehydropantoate 2-reductase PanE, putative	6.07	32.36
512	SACOL2618	Q5HCV0	Ldh2	1	L-lactate dehydrogenase	4.54	34.42
513	SACOL2622	Q5HCU6	FdaB	2	fructose-bisphosphate aldolase, class I	4.65	33.05
514	SACOL2623	Q5HCU5	Mqo2	3	malate:quinone oxidoreductase	6.52	56
515	SACOL2624	Q5HCU4	-	1	acetyl-CoA synthetase, putative	5.5	59.75
516	SACOL2650	Q5HCR9	-	1	transcriptional regulator, putative	7.09	17.69
517	SACOL2667	Q5HCQ2	-	1	isochorismatase family protein	5.32	20.73
518	SACOL2708	Q5HCL3	-	1	ABC transporter, ATP-binding protein	6.01	64.14
519	SACOL2710	Q5HCL1	-	1	conserved hypothetical protein	5.94	31.44
520	SACOL2722	Q5HCJ9	-	1	N-acetyltransferase family protein	6.44	30.44
521	SACOL2737	Q5HCI4	GidA	8	glucose inhibited division protein A	5.15	70.12

[1] based on JCVI CMR (http://cmr.jcvi.org/)
[2] Uniprot accession number (http://www.uniprot.org/)
[3] number of associated protein spots on the reference map
