# Supplementary material for: Aureolib — A Proteome Signature Library: Towards an Understanding of Staphylococcus aureus Pathophysiology
Source: PLoS One. 2013 Aug 13;8(8):e70669. doi: 10.1371/journal.pone.0070669 (PMC3742771; doi:10.1371/journal.pone.0070669)
Supplement: Table S3 — Hypothetical proteins identified on the reference map. (PDF) [file pone.0070669.s010.pdf]

Supplementary Table S3. Hypothetical proteins identified on the reference map

Protein	Protein	Protein	Protein	Protein	Protein	Protein
SACOL0051	SACOL0467	SACOL0721	SACOL1200	SACOL1447	SACOL1792	SACOL2133
SACOL0157	SACOL0495	SACOL0776	SACOL1201	SACOL1464	SACOL1793	SACOL2136
SACOL0271	SACOL0521	SACOL0785	SACOL1219	SACOL1483	SACOL1895	SACOL2163
SACOL0279	SACOL0565	SACOL0830	SACOL1239	SACOL1620	SACOL1902	SACOL2196
SACOL0314	SACOL0579	SACOL0879	SACOL1307	SACOL1627	SACOL1968	SACOL2344
SACOL0427	SACOL0597	SACOL0930	SACOL1349	SACOL1630	SACOL1975	SACOL2379
SACOL0435	SACOL0613	SACOL1008	SACOL1366	SACOL1649	SACOL1985	SACOL2518
SACOL0445	SACOL0614	SACOL1009	SACOL1386	SACOL1651	SACOL1992	SACOL2519
SACOL0455	SACOL0615	SACOL1089	SACOL1387	SACOL1670	SACOL2020	SACOL2596
SACOL0456	SACOL0633	SACOL1120	SACOL1402	SACOL1672	SACOL2035	SACOL2609
SACOL0457	SACOL0656	SACOL1163	SACOL1413	SACOL1688	SACOL2106	SACOL2710
