# Supplementary material for: Aureolib — A Proteome Signature Library: Towards an Understanding of Staphylococcus aureus Pathophysiology
Source: PLoS One. 2013 Aug 13;8(8):e70669. doi: 10.1371/journal.pone.0070669 (PMC3742771; doi:10.1371/journal.pone.0070669)
Supplement: Table S4 — Mass spectrometry results for the identified proteins. (PDF) [file pone.0070669.s011.pdf]

Supplementary Table S4. Mass spectrometry results for the identified proteins.

Label[1]	Locus	Symbol	GI#	protein score[2]	total ion score[3]	sequence coverage
AccA 1	SACOL1747	AccA	57650570	251	169	0.3
AccA 2	SACOL1747	AccA	57650570	98	40	0.24
AccC	SACOL1571	AccC	57650483	163	69	0.21
AckA 1	SACOL1760	AckA	57652009	411	108	0.73
AckA 2	SACOL1760	AckA	57652009	284	73	0.49
AcnA 1	SACOL1385	AcnA	57650354	454	137	0.44
AcnA 2	SACOL1385	AcnA	57650354	447	97	0.43
Adk	SACOL2218	Adk	57650813	127	-	0.55
AhpC 1	SACOL0452	AhpC	57652639	436	329	0.61
AhpC 2	SACOL0452	AhpC	57652639	50	43	0.07
AhpC 3	SACOL0452	AhpC	57652639	225	144	0.46
AhpF	SACOL0451	AhpF	57652638	250	95	0.44
AlaS	SACOL1673	AlaS	57650500	262	132	0.3
AldA2	SACOL1984	AldA2	57650674	56	-	0.19
AnsA	SACOL1519	AnsA	57650433	159	85	0.31
ArgR	SACOL1565	ArgR	57650477	387	275	0.52
ArgS	SACOL0663	ArgS	57650073	88	-	0.31
AroB	SACOL1505	AroB	57650420	239	164	0.36
AroC	SACOL1506	AroC	57650421	336	254	0.22
AroE	SACOL1652	AroE	57651988	212	87	0.54
Asd	SACOL1429	Asd	57651894	394	213	0.53
Asp23 1	SACOL2173	Asp23	#NV	59	26	0.33
Asp23 2	SACOL2173	Asp23	#NV	192	129	0.33
Asp23 3	SACOL2173	Asp23	#NV	336	165	0.82
Asp23 4	SACOL2173	Asp23	#NV	336	227	0.68
Asp23 5	SACOL2173	Asp23	57652199	278	163	0.68
Asp23 6	SACOL2173	Asp23	57286385	276	141	0.67
AspS 1	SACOL1685	AspS	57650511	461	162	0.58
AspS 2	SACOL1685	AspS	57650511	167	37	0.27
AtpA 1	SACOL2097	AtpA	57652127	418	199	0.42
AtpA 2	SACOL2097	AtpA	57652127	271	138	0.28
AtpC	SACOL2094	AtpC	57652124	106	28	0.48
AtpD 1	SACOL2095	AtpD	57652125	505	261	0.66
AtpG	SACOL2096	AtpG	57652126	343	222	0.43
AtpH	SACOL2098	AtpH	57652128	341	173	0.51
Bcp	SACOL1921	Bcp	57650653	230	118	0.43
Cap5G	SACOL0142	Cap5G	57651159	336	72	0.64
Cap5O	SACOL0150	Cap5O	57651167	264	104	0.4
Cap5P	SACOL0151	Cap5P	57651168	282	77	0.44
CarA	SACOL1214	CarA	57651771	340	265	0.18
CarB	SACOL1215	CarB	57651772	141	41	0.2
Cbf1	SACOL1898	Cbf1	57650631	401	154	0.73
CcpA	SACOL1786	CcpA	57652034	323	123	0.53
ClpB 1	SACOL0979	ClpB	57651666	547	242	0.45

Label[1]	Locus	Symbol	GI#	protein score[2]	total ion score[3]	sequence coverage
ClpB 2	SACOL0979	ClpB	57651666	594	243	0.51
ClpP 1	SACOL0833	ClpP	57650109	119	50	0.34
ClpP 2	SACOL0833	ClpP	57650109	89	48	0.18
CoaBC	SACOL1223	CoaBC	57651780	299	128	0.59
CoaD	SACOL1134	CoaD	57651734	215	76	0.75
CoaE	SACOL1735	CoaE	57650559	337	147	0.6
CodY 1	SACOL1272	CodY	57651824	582	268	0.81
CodY 2	SACOL1272	CodY	57651824	255	115	0.41
CscA	SACOL2029	CscA	57650713	156	57	0.29
CtsR 1	SACOL0567	CtsR	57651399	244	116	0.56
CtsR 2	SACOL0567	CtsR	57651399	190	85	0.54
CysK 1	SACOL0557	CysK	57651389	440	148	0.82
CysK 2	SACOL0557	CysK	57651389	230	100	0.46
CysK 3	SACOL0557	CysK	57651389	327	117	0.81
CysS	SACOL0576	CysS	57651406	276	158	0.3
DapA	SACOL1430	DapA	57651895	437	218	0.68
DapB 1	SACOL1431	DapB	57651896	496	343	0.63
DapB 2	SACOL1431	DapB	57651896	262	145	0.47
DapD	SACOL1432	DapD	57651897	174	137	0.29
Dat	SACOL1800	Dat	57652048	521	177	0.97
Def1	SACOL1100	Def1	57651700	215	118	0.56
DeoB	SACOL0124	DeoB	57651145	204	120	0.37
DeoC1	SACOL0123	DeoC1	57651144	348	205	0.54
DeoD2	SACOL2130	DeoD2	57652160	100	50	0.38
DltA 1	SACOL0935	DltA	57651624	440	271	0.37
DltA 2	SACOL0935	DltA	57651624	256	150	0.23
DnaB	SACOL0016	DnaB	57651124	271	124	0.32
DnaJ 1	SACOL1636	DnaJ	57651972	218	135	0.41
DnaK	SACOL1637	DnaK	57651973	309	78	0.5
DnaN 1	SACOL0002	DnaN	57651110	300	126	0.45
DnaN 2	SACOL0002	DnaN	57651110	518	234	0.62
DnaX	SACOL0520	DnaX	57651359	200	34	0.47
Efp	SACOL1587	Efp	57651923	194	116	0.4
Eno 1	SACOL0842	Eno	57650118	596	334	0.57
Eno 2	SACOL0842	Eno	57650118	472	284	0.48
Eno 3	SACOL0842	Eno	57650118	510	333	0.51
Eno 4	SACOL0842	Eno	57650118	561	294	0.58
Era	SACOL1624	Era	57651960	447	253	0.53
Est	SACOL0845	Est	57650121	318	244	0.27
FabF	SACOL0988	FabF	57650176	234	120	0.27
FabG1	SACOL1245	FabG1	57651799	433	294	0.6
FabH	SACOL0987	FabH	57650175	382	254	0.39
FabI 1	SACOL1016	FabI	57650202	232	192	0.13
FabI 2	SACOL1016	FabI	57650202	422	228	0.64
FabI 3	SACOL1016	FabI	57650202	405	239	0.63

Label[1]	Locus	Symbol	GI#	protein score[2]	total ion score[3]	sequence coverage
FabZ	SACOL2091	FabZ	57650772	168	109	0.52
FbaA 1	SACOL2117	FbaA	57652147	176	129	0.27
FbaA 2	SACOL2117	FbaA	57652147	152	61	152
FbaA 3	SACOL2117	FbaA	57652147	333	144	0.64
FbaA 4	SACOL2117	FbaA	57652147	171	86	0.38
FdaB 1	SACOL2622	FdaB	57652341	282	122	0.59
FdaB 2	SACOL2622	FdaB	57652341	221	84	0.48
FemC 1	SACOL1329	FemC	57650300	304	97	0.49
FemC 2	SACOL1329	FemC	57650300	297	176	0.35
Fhs 1	SACOL1782	Fhs	57652030	424	292	0.33
Fhs 2	SACOL1782	Fhs	57652030	243	195	0.12
FolA 1	SACOL1461	FolA	57650382	258	189	0.33
FolA 2	SACOL1461	FolA	57650382	353	247	0.33
FolD	SACOL1072	FolD	57651672	391	282	0.4
Frp	SACOL2534	Frp	57651031	220	102	0.49
FtsA	SACOL1198	FtsA	57651755	159	-	0.42
FtsZ	SACOL1199	FtsZ	57651756	331	201	0.35
FumC	SACOL1908	FumC	57650640	135	55	0.27
FusA 1	SACOL0593	FusA	57651423	379	127	0.55
FusA 2	SACOL0593	FusA	57651423	320	145	0.43
GalM	SACOL2332	GalM	57650881	195	95	0.46
GapA1 1	SACOL0838	GapA1	57650114	218	104	0.4
GapA1 2	SACOL0838	GapA1	57650114	208	97	0.46
GapR	SACOL0837	GapR	57650113	110	-	0.39
GcvH 1	SACOL0877	GcvH	57650151	179	155	0.44
GcvH 2	SACOL0877	GcvH	57650151	287	253	0.44
GidA	SACOL2737	GidA	57651105	279	125	0.3
Glk	SACOL1604	Glk	57651940	269	143	0.57
GlmM	SACOL2151	GlmM	57652179	145	27	0.26
GlmS 1	SACOL2145	GlmS	57652173	420	240	0.3
GlmS 2	SACOL2145	GlmS	57652173	515	252	0.51
GlpD 1	SACOL1321	GlpD	57651870	305	108	0.41
GlpD 2	SACOL1321	GlpD	57651870	266	49	0.45
GlpD 3	SACOL1321	GlpD	57651870	158	14	0.3
GlpK	SACOL1320	GlpK	57651869	337	162	0.38
GltA	SACOL1742	GltA	57650566	338	127	0.52
GltD	SACOL0515	GltD	57651354	142	109	0.18
GltX	SACOL0574	GltX	57651404	375	90	0.52
GluD	SACOL0961	GluD	57651649	366	153	0.44
GlyA 1	SACOL2105	GlyA	57652135	264	172	0.22
GlyA 2	SACOL2105	GlyA	57652135	405	200	0.38
GlyA 3	SACOL2105	GlyA	57652135	393	207	0.4
GlyS 1	SACOL1622	GlyS	57651958	103	66	0.14
GlyS 2	SACOL1622	GlyS	57651958	515	344	0.53
GlyS 3	SACOL1622	GlyS	57651958	107	68	

Label[1]	Locus	Symbol	GI#	protein score[2]	total ion score[3]	sequence coverage
Gmk	SACOL1221	Gmk	57651778	91	-	0.42
Gnd	SACOL1554	Gnd	57650466	370	189	0.45
Gpm	SACOL2415	Gpm	57650960	248	109	0.67
GpsA	SACOL1514	GpsA	57650429	183	62	0.37
GpxA1	SACOL1325	GpxA1	57651874	239	142	0.51
GreA 1	SACOL1665	GreA 1	57652001	206	98	0.6
GreA 2	SACOL1665	GreA 2	57652001	80	26	0.4
GroEL	SACOL2016	GroEL	57650701	527	289	0.49
GroES	SACOL2017	GroES	57650702	176	58	0.89
GrpE	SACOL1638	GrpE	57651974	347	271	0.33
GuaA 1	SACOL0461	GuaA	57652648	333	167	0.38
GuaA 2	SACOL0461	GuaA	57652648	304	184	0.27
GuaB 2	SACOL0460	GuaB	57652647	387	177	0.72
GuaC	SACOL1371	GuaC	57650340	108	-	0.52
GyrA 1	SACOL0006	GyrA	57651114	69	32	0.17
GyrA 2	SACOL0006	GyrA	57651114	216	67	0.33
GyrB 1	SACOL0005	GyrB	57651113	379	69	0.52
GyrB 2	SACOL0005	GyrB	57651113	128	58	0.15
HemL1	SACOL1714	HemL1	57650539	346	57	0.5
HemL2	SACOL1922	HemL2	57652083	485	322	0.57
HexB	SACOL1316	HexB	57651866	170	113	0.2
Hom	SACOL1362	Hom	57650331	288	155	0.37
HprK	SACOL0825	HprK	57650101	290	56	0.66
HslU 1	SACOL1271	HslU	57651823	257	127	0.39
HslU 2	SACOL1271	HslU	57651823	241	142	0.34
HslV	SACOL1270	HslV	57651822	156	83	0.4
HutG	SACOL2327	HutG	57650876	426	309	0.32
Icd 1	SACOL1741	Icd	57650565	332	147	0.43
Icd 2	SACOL1741	Icd	57650565	308	203	0.33
IleS 2	SACOL1206	IleS	57651763	133	77	0.16
IlvA2 1	SACOL2050	IlvA2	57650733	361	121	0.48
IlvA2 2	SACOL2050	IlvA2	57650733	334	125	0.5
IlvA2 3	SACOL2050	IlvA2	57650733	159	70	0.41
IlvB 1	SACOL2043	IlvB	57650726	379	230	0.38
IlvB 2	SACOL2043	IlvB	57650726	300	206	0.28
IlvB 3	SACOL2043	IlvB	57650726	210	128	0.33
IlvC 1	SACOL2045	IlvC	57650728	563	360	0.55
IlvC 2	SACOL2045	IlvC	57650728	464	336	0.52
IlvC 3	SACOL2045	IlvC	57650728	381	255	0.69
IlvC 4	SACOL2045	IlvC	57650728	827	521	0.91
IlvD 1	SACOL2042	IlvD	57650725	421	222	0.45
IlvD 2	SACOL2042	IlvD	57650725	463	283	0.54
IlvD 3	SACOL2042	IlvD	57650725	237	83	0.39
IlvE	SACOL0600	IlvE	57651430	343	150	0.67
InfB	SACOL1288	InfB	57651839	276	23	0.49

Label[1]	Locus	Symbol	GI#	protein score[2]	total ion score[3]	sequence coverage
IpdC	SACOL0173	IpdC	57651189	239	176	0.35
IspA	SACOL1566	IspA	57650478	105	38	0.44
IspD	SACOL0236	IspD	57652511	121	37	0.52
KatA 1	SACOL1368	KatA	57650337	339	182	0.47
KatA 2	SACOL1368	KatA	57650337	304	123	0.43
KatA 3	SACOL1368	KatA	57650337	328	148	0.51
KatA 4	SACOL1368	KatA	57650337	177	76	0.23
KatA 5	SACOL1368	KatA	57650337	300	162	0.48
KsgA	SACOL0536	KsgA	57651375	194	70	0.42
Ldh1 1	SACOL0222	Ldh1	57652499	207	129	0.21
Ldh1 2	SACOL0222	Ldh1	57652499	320	159	0.59
Ldh2	SACOL2618	Ldh2	57652337	501	280	0.69
LeuA 1	SACOL2046	LeuA	57650729	321	148	0.45
LeuA 2	SACOL2046	LeuA	57650729	573	305	0.64
LeuA 3	SACOL2046	LeuA	57650729	457	231	0.64
LeuB	SACOL2047	LeuB	57650730	465	293	0.62
LeuC 1	SACOL2048	LeuC	57650731	287	139	0.36
LeuC 2	SACOL2048	LeuC	57650731	171	92	0.33
LeuD	SACOL2049	LeuD	57650732	244	132	0.74
LeuS	SACOL1808	LeuS	57652056	54	25	0.13
LigA 1	SACOL1965	LigA	57650655	126	65	0.21
LigA 2	SACOL1965	LigA	57650655	169	85	0.33
LuxS	SACOL2126	LuxS	57652156	87	57	0.27
LysA 1	SACOL1435	LysA	57651900	475	157	0.58
LysA 2	SACOL1435	LysA	57651900	153	43	0.5
LysS 1	SACOL0562	LysS	57651394	375	184	0.41
LysS 2	SACOL0562	LysS	57651394	171	45	0.32
ManA1	SACOL2135	ManA1	57652165	106	61	0.14
MenB	SACOL1054	MenB	57650239	138	14	0.42
MenD	SACOL1052	MenD	57650237	122	38	0.21
MetE	SACOL0428	MetE	57652616	72	21	0.17
MetK 1	SACOL1837	MetK	57650575	393	234	0.39
MetK 2	SACOL1837	MetK	57650575	398	230	0.25
MiaA	SACOL1323	MiaA	57651872	54	15	0.22
MoaB	SACOL2268	MoaB	57652208	248	104	0.89
MoaE	SACOL2264	MoaE	57650856	108	33	0.58
Mqo1	SACOL2362	Mqo1	57650909	287	184	0.26
Mqo2 1	SACOL2623	Mqo2	57652342	255	172	0.34
Mqo2 2	SACOL2623	Mqo2	57652342	225	172	0.23
Mqo2 3	SACOL2623	Mqo2	57652342	144	80	0.25
MraZ	SACOL1191	MraZ	57650290	162	75	0.65
MtlD	SACOL2149	MtlD	57652177	400	182	0.51
Mtn	SACOL1655	Mtn	57651991	257	167	0.4
MurAA	SACOL2092	MurAA	57650773	225	63	0.52
MurAB 1	SACOL2116	MurAB	57652146	181	86	0.27

Label[1]	Locus	Symbol	GI#	protein score[2]	total ion score[3]	sequence coverage
MurAB 2	SACOL2116	MurAB	57652146	236	154	0.37
MurC	SACOL1790	MurC	57652038	377	228	0.39
MurD	SACOL1196	MurD	57650295	294	203	0.3
MurE	SACOL1023	MurE	57650209	495	275	0.49
MvaD	SACOL0637	MvaD	57651466	117	95	
NadE	SACOL1974	NadE	57650664	363	154	0.74
Nfo	SACOL1614	Nfo	57651950	369	203	0.59
NrdE 1	SACOL0792	NrdE	57651568	114	82	0.06
NrdE 2	SACOL0792	NrdE	57651568	367	203	0.44
NrdF 1	SACOL0793	NrdF	57651569	405	219	0.58
NrdF 2	SACOL0793	NrdF	57651569	162	105	0.32
NusB	SACOL1569	NusB	57650481	148	52	0.7
NusG	SACOL0582	NusG	57651412	303	205	0.6
PabA	SACOL0773	PabA	57651552	145	51	0.79
PanB	SACOL2615	PanB	57652334	388	183	0.63
ParC 1	SACOL1390	ParC	57650359	234	60	0.42
ParC 2	SACOL1390	ParC	57650359	113	8	0.28
PdhA 1	SACOL1102	PdhA	57651702	137	32	0.44
PdhA 2	SACOL1102	PdhA	57651702	134	54	0.25
PdhB	SACOL1103	PdhB	57651703	425	193	0.67
PdhC 1	SACOL1104	PdhC	57651704	251	147	0.3
PdhC 2	SACOL1104	PdhC	57651704	81	-	
PdhC 3	SACOL1104	PdhC	57651704	376	278	0.36
PdhD 1	SACOL1105	PdhD	57651705	516	294	0.43
PdhD 2	SACOL1105	PdhD	57651705	434	173	0.48
PdhD 3	SACOL1105	PdhD	57651705	460	341	0.44
PepA1	SACOL1795	PepA1	57652043	283	160	0.37
PepF	SACOL1005	PepF	57650193	200	41	0.25
PepQ	SACOL1756	PepQ	57652005	255	160	0.33
PepS 1	SACOL1937	PepS	57652098	338	136	0.54
PepS 2	SACOL1937	PepS	57652098	112	-	0.47
PfkA 1	SACOL1746	PfkA	57650569	99	21	0.33
PfkA 2	SACOL1746	PfkA	57650569	249	65	0.57
PfkA 3	SACOL1746	PfkA	57650569	218	51	0.38
PflB 3	SACOL0204	PflB	57651220	189	9	0.37
PflB 4	SACOL0204	PflB	57651220	250	83	0.35
PflB 5	SACOL0204	PflB	57651220	167	43	0.26
Pgi	SACOL0966	Pgi	57651654	404	173	0.63
Pgk 1	SACOL0839	Pgk	57650115	294	137	0.54
Pgk 2	SACOL0839	Pgk	57650115	240	99	0.55
Pgk 3	SACOL0839	Pgk	57650115	222	153	0.39
Pgk 4	SACOL0839	Pgk	57650115	350	242	0.51
Pgm 1	SACOL0841	Pgm	57650117	405	194	0.45
Pgm 2	SACOL0841	Pgm	57650117	506	349	0.39
Pgm 4	SACOL0841	Pgm	57650117	298	157	0.31

Label[1]	Locus	Symbol	GI#	protein score[2]	total ion score[3]	sequence coverage
PheS	SACOL1148	PheS	57651747	248	41	0.63
PheT	SACOL1149	PheT	57651748	471	211	0.42
PlsX	SACOL1243	PlsX	57651797	300	111	0.71
PnbA	SACOL2459	PnbA	57652268	99	21	0.3
Pnp	SACOL1293	Pnp	57651844	234	119	0.31
PolA	SACOL1737	PolA	57650561	541	357	0.35
PpaC	SACOL1982	PpaC	57650672	224	140	0.41
PrfA	SACOL2110	PrfA	57652140	325	172	0.55
PriA 1	SACOL1224	PriA	57651781	241	129	0.25
ProC	SACOL1546	ProC	57650459	266	163	0.59
ProS 1	SACOL1282	ProS	57651833	505	227	0.53
ProS 2	SACOL1282	ProS	57651833	266	112	0.32
PrsA 1	SACOL0544	PrsA	57650041	495	252	0.76
PrsA 2	SACOL0544	PrsA	57650041	441	262	0.79
PrsA 3	SACOL0544	PrsA	57650041	356	212	0.54
PrsA 4	SACOL0544	PrsA	57650041	51	-	0.39
Pta	SACOL0634	Pta	57651463	482	310	0.62
PtsI 1	SACOL1092	PtsI	57651692	300	174	0.38
PtsI 2	SACOL1092	PtsI	57651692	252	132	0.32
PurA	SACOL0018	PurA	57651126	434	260	0.5
PurB	SACOL1969	PurB	57650659	403	222	0.45
PurC	SACOL1075	PurC	57651675	305	64	0.77
PurD	SACOL1083	PurD	57651683	343	185	0.45
PurE	SACOL1073	PurE	57651673	64	-	0.78
PurF 1	SACOL1079	PurF	57651679	333	152	0.41
PurF 2	SACOL1079	PurF	57651679	71	-	0.28
PurH 1	SACOL1082	PurH	57651682	454	215	0.44
PurH 2	SACOL1082	PurH	57651682	363	195	0.32
PurH 3	SACOL1082	PurH	57651682	371	212	0.32
PurK	SACOL1074	PurK	57651674	241	138	0.26
PurM	SACOL1080	PurM	57651680	316	207	0.35
PurN	SACOL1081	PurN	57651681	172	110	0.27
PurQ	SACOL1077	PurQ	57651677	297	158	0.78
Pyc 1	SACOL1123	Pyc	57651723	99	25	0.24
Pyc 2	SACOL1123	Pyc	57651723	349	106	0.4
Pyc 3	SACOL1123	Pyc	57651723	539	318	0.36
Pyk 1	SACOL1745	Pyk	57650568	381	149	0.5
Pyk 2	SACOL1745	Pyk	57650568	405	163	0.41
Pyk 3	SACOL1745	Pyk	57650568	301	263	0.22
PyrB	SACOL1212	PyrB	57651769	423	120	0.61
PyrC	SACOL1213	PyrC	57651770	246	108	0.64
PyrE	SACOL1217	PyrE	57651774	82	11	0.58
PyrF	SACOL1216	PyrF	57651773	364	145	0.93
PyrG	SACOL2119	PyrG	57652149	225	76	0.45
PyrH	SACOL1277	PyrH	57651828	371	271	0.42

Label[1]	Locus	Symbol	GI#	protein score[2]	total ion score[3]	sequence coverage
PyrR	SACOL1210	PyrR	57651767	403	183	0.93
QueA	SACOL1695	QueA	57650521	200	89	0.3
RadA	SACOL0572	RadA	57651402	276	118	0.37
RecN	SACOL1564	RecN	57650476	161	43	0.4
RelA1	SACOL1010	RelA1	57650198	571	431	0.66
RexA	SACOL0971	RexA	57651659	115	82	0.07
RibD	SACOL1820	RibD	57652067	73	9	0.25
RibF	SACOL1291	RibF	57651842	368	149	0.61
RibH	SACOL1817	RibH	57652064	445	280	0.7
RplD	SACOL2238	RplD	57650833	208	62	0.79
RplJ	SACOL0585	RplJ	57651415	210	40	0.81
RplL	SACOL0586	RplL	57651416	159	146	0.18
RplO	SACOL2220	RplO	57650815	155	80	0.48
RplY	SACOL0545	RplY	57650042	231	130	0.41
RpoA	SACOL2213	RpoA	57650808	245	83	0.5
RpoC 1	SACOL0589	RpoC	57651419	168	80	0.16
RpoC 2	SACOL0589	RpoC	57651419	209	124	0.2
RpoD	SACOL1618	RpoD	57651954	236	97	0.42
RpoF	SACOL2054	RpoF	57650736	92	-	0.38
RpsA	SACOL1516	RpsA	57650431	419	244	0.59
RpsB 1	SACOL1274	RpsB	57651825	308	121	0.64
RpsB 2	SACOL1274	RpsB	57651825	228	59	0.62
RpsD 1	SACOL1769	RpsD	57652018	334	191	0.59
RpsD 2	SACOL1769	RpsD	57652018	85	79	0.09
RpsD 3	SACOL1769	RpsD	57652018	332	151	0.66
RpsF 1	SACOL0437	RpsF	57652625	227	179	0.65
RpsF 2	SACOL0437	RpsF	57652625	194	146	0.65
RpsO	SACOL1292	RpsO	57651843	60	26	0.49
RsbU	SACOL2057	RsbU	57650739	227	93	0.32
RsbV 1	SACOL2056	RsbV	57650738	90	40	0.92
RsbV 2	SACOL2056	RsbV	57650738	302	217	0.72
RsbW 1	SACOL2055	RsbW	57650737	62	-	0.4
RsbW 2	SACOL2055	RsbW	57650737	348	203	0.59
RuvA	SACOL1697	RuvA	57650523	160	104	0.39
SACOL0004	SACOL0004	-	57651112	136	-	0.44
SACOL0012	SACOL0012	-	57651120	192	141	0.19
SACOL0051	SACOL0051	-	57652420	134	104	0.07
SACOL0111 1	SACOL0111	-	57652474	310	182	0.59
SACOL0111 2	SACOL0111	-	57652474	347	226	0.44
SACOL0135 1	SACOL0135	-	57651152	224	129	0.29
SACOL0135 2	SACOL0135	-	57651152	151	77	0.26
SACOL0135 3	SACOL0135	-	57651152	118	30	0.1
SACOL0157	SACOL0157	-	57651174	327	244	0.4
SACOL0162	SACOL0162	-	57651178	182	149	0.13
SACOL0240	SACOL0240	-	#NV	358	196	0.68

Label[1]	Locus	Symbol	GI#	protein score[2]	total ion score[3]	sequence coverage
SACOL0241	SACOL0241	-	57652516	256	186	0.36
SACOL0243 1	SACOL0243	-	57652518	134	24	0.36
SACOL0243 5	SACOL0243	-	57652518	61	1	
SACOL0257	SACOL0257	-	57652531	71	-	0.32
SACOL0271	SACOL0271	-	57652545	276	157	0.64
SACOL0279	SACOL0279	-	57652553	67	27	0.38
SACOL0314	SACOL0314	-	57651241	125	67	0.46
SACOL0399	SACOL0399	-	57652588	149	47	0.21
SACOL0427 1	SACOL0427	-	57652615	588	409	0.75
SACOL0427 2	SACOL0427	-	57652615	198	126	0.43
SACOL0429 1	SACOL0429	-	57652617	511	305	0.46
SACOL0429 2	SACOL0429	-	57652617	235	80	0.42
SACOL0429 3	SACOL0429	-	57652617	64	9	0.28
SACOL0429 4	SACOL0429	-	57652617	542	240	0.47
SACOL0430 1	SACOL0430	-	57652618	346	158	0.51
SACOL0430 2	SACOL0430	-	57652618	300	151	0.48
SACOL0430 3	SACOL0430	-	57652618	304	157	0.53
SACOL0431 2	SACOL0431	-	57652619	154	111	0.15
SACOL0431 3	SACOL0431	-	57652619	318	156	0.35
SACOL0431 4	SACOL0431	-	57652619	314	155	0.38
SACOL0435	SACOL0435	-	57652623	577	371	0.64
SACOL0445	SACOL0445	-	57652632	109	36	0.73
SACOL0456	SACOL0456	-	57652643	217	102	0.63
SACOL0457	SACOL0457	-	57652644	60	5	0.51
SACOL0467	SACOL0467	-	57651308	129	76	0.25
SACOL0495	SACOL0495	-	57651334	99	38	0.19
SACOL0503	SACOL0503	-	57651342	194	122	0.29
SACOL0509 1	SACOL0509	-	57651348	75	44	0.3
SACOL0509 2	SACOL0509	-	57651348	60	39	0.29
SACOL0521	SACOL0521	-	57651360	45	37	0.2
SACOL0534	SACOL0534	-	57651373	287	172	0.43
SACOL0540	SACOL0540	-	57650037	85	44	0.54
SACOL0549	SACOL0549	-	57650046	154	118	0.24
SACOL0556	SACOL0556	-	57651388	352	233	0.33
SACOL0565	SACOL0565	-	57651397	429	216	0.7
SACOL0578	SACOL0578	-	57651408	336	203	0.49
SACOL0579	SACOL0579	-	57651409	106	36	0.57
SACOL0596	SACOL0596	-	57651426	167	61	0.31
SACOL0597	SACOL0597	-	57651427	206	80	0.5
SACOL0602	SACOL0602	-	57651432	288	235	0.29
SACOL0603	SACOL0603	-	57651433	160	89	0.51
SACOL0607	SACOL0607	-	57651436	427	250	0.78
SACOL0613	SACOL0613	-	57651442	310	153	0.44
SACOL0614	SACOL0614	-	57651443	189	83	0.41
SACOL0615	SACOL0615	-	57651444	56	37	0.24

Label[1]	Locus	Symbol	GI#	protein score[2]	total ion score[3]	sequence coverage
SACOL0617	SACOL0617	-	57651446	300	203	0.5
SACOL0618 1	SACOL0618	-	57651447	92	58	0.26
SACOL0618 2	SACOL0618	-	57651447	380	324	0.26
SACOL0633	SACOL0633	-	57651462	321	204	0.38
SACOL0638	SACOL0638	-	57650049	200	73	0.47
SACOL0656	SACOL0656	-	57650067	51	23	0.41
SACOL0658	SACOL0658	-	57650068	383	159	0.43
SACOL0660	SACOL0660	-	57650070	121	43	0.34
SACOL0668	SACOL0668	-	57650078	324	265	0.44
SACOL0721	SACOL0721	-	57651504	280	211	0.33
SACOL0731	SACOL0731	-	57651513	153	37	0.48
SACOL0776	SACOL0776	-	57651553	386	179	0.72
SACOL0785	SACOL0785	-	57651561	207	78	0.56
SACOL0789 1	SACOL0789	-	57651565	140	93	0.31
SACOL0789 2	SACOL0789	-	57651565	72	60	0.06
SACOL0815	SACOL0815	-	57650091	270	151	0.44
SACOL0821	SACOL0821	-	57650097	419	275	0.53
SACOL0830	SACOL0830	-	57650106	103	-	0.43
SACOL0872	SACOL0872	-	57650146	118	10	0.8
SACOL0875	SACOL0875	-	57650149	188	102	0.68
SACOL0879	SACOL0879	-	57650152	126	61	0.31
SACOL0890	SACOL0890	-	57650163	65	26	0.54
SACOL0914	SACOL0914	-	57651604	192	57	0.62
SACOL0916 1	SACOL0916	-	57651606	455	244	0.63
SACOL0916 2	SACOL0916	-	57651606	345	224	0.44
SACOL0930	SACOL0930	-	57651619	172	50	0.91
SACOL0931	SACOL0931	-	57651620	349	246	0.43
SACOL0932	SACOL0932	-	57651621	272	187	0.38
SACOL0939 1	SACOL0939	-	57651628	49	21	0.38
SACOL0939 2	SACOL0939	-	57651628	161	134	0.38
SACOL0944	SACOL0944	-	57651632	294	98	0.44
SACOL0945 1	SACOL0945	-	57651633	295	227	0.08
SACOL0945 2	SACOL0945	-	57651633	437	325	0.42
SACOL0957	SACOL0957	-	57651645	235	133	0.46
SACOL0959	SACOL0959	-	57651647	363	243	0.49
SACOL0973 2	SACOL0973	-	57651660	195	66	0.69
SACOL0973 3	SACOL0973	-	57651660	201	79	0.71
SACOL0975	SACOL0975	-	57651662	304	61	0.59
SACOL0976	SACOL0976	-	57651663	279	112	0.59
SACOL1003	SACOL1003	-	57650191	85	45	0.15
SACOL1008	SACOL1008	-	57650196	65	-	0.4
SACOL1009	SACOL1009	-	57650197	110	62	0.79
SACOL1034	SACOL1034	-	57650219	119	70	0.35
SACOL1058	SACOL1058	-	57650243	217	123	0.42
SACOL1089	SACOL1089	-	57651689	322	152	0.43

Label[1]	Locus	Symbol	GI#	protein score[2]	total ion score[3]	sequence coverage
SACOL1096	SACOL1096	-	57651696	185	107	0.52
SACOL1108	SACOL1108	-	57651708	374	257	0.39
SACOL1120	SACOL1120	-	57651720	176	72	0.69
SACOL1163	SACOL1163	-	57650262	269	138	0.7
SACOL1189	SACOL1189	-	57650288	373	275	0.48
SACOL1192	SACOL1192	-	57650291	502	269	0.67
SACOL1200	SACOL1200	-	57651757	239	128	0.47
SACOL1201	SACOL1201	-	57651758	194	72	0.54
SACOL1205	SACOL1205	-	57651762	277	219	0.33
SACOL1209	SACOL1209	-	57651766	328	231	0.36
SACOL1219	SACOL1219	-	57651776	79	59	0.35
SACOL1236	SACOL1236	-	57651791	68	-	0.56
SACOL1239	SACOL1239	-	57651793	107	21	0.78
SACOL1285	SACOL1285	-	57651836	225	-	0.47
SACOL1294 1	SACOL1294	-	57651845	316	75	0.35
SACOL1294 2	SACOL1294	-	57651845	140	51	0.11
SACOL1296	SACOL1296	-	57651847	141	69	0.5
SACOL1304	SACOL1304	-	57651855	334	142	0.48
SACOL1307	SACOL1307	-	57651858	463	196	0.75
SACOL1308	SACOL1308	-	57651859	297	135	0.35
SACOL1349	SACOL1349	-	57650318	88	13	0.22
SACOL1360	SACOL1360	-	57650329	85	-	0.46
SACOL1365	SACOL1365	-	57650334	164	124	0.25
SACOL1366	SACOL1366	-	57650335	103	32	0.52
SACOL1386	SACOL1386	-	57650355	101	10	0.6
SACOL1387	SACOL1387	-	57650356	148	101	0.52
SACOL1402	SACOL1402	-	57650370	117	11	
SACOL1411	SACOL1411	-	57651876	297	196	0.34
SACOL1413	SACOL1413	-	57651878	81	25	0.38
SACOL1419	SACOL1419	-	57651884	88	8	0.26
SACOL1427 1	SACOL1427	-	57651892	404	199	0.39
SACOL1427 2	SACOL1427	-	57651892	252	160	0.26
SACOL1433	SACOL1433	-	57651898	398	195	0.46
SACOL1434	SACOL1434	-	57651899	273	104	0.53
SACOL1437	SACOL1437	-	57651902	188	154	0.78
SACOL1441	SACOL1441	-	57651906	166	62	0.3
SACOL1445	SACOL1445	-	57651910	122	-	0.5
SACOL1447 1	SACOL1447	-	57651912	77	54	0.29
SACOL1447 2	SACOL1447	-	57651912	132	92	0.33
SACOL1457	SACOL1457	-	57650380	307	185	0.65
SACOL1460	SACOL1460	-	57650381	131	52	0.34
SACOL1464	SACOL1464	-	57650384	76	23	0.41
SACOL1483	SACOL1483	-	57650399	199	147	0.21
SACOL1503 1	SACOL1503	-	57650418	84	13	0.29
SACOL1503 2	SACOL1503	-	57650418	124	59	0.4

Label[1]	Locus	Symbol	GI#	protein score[2]	total ion score[3]	sequence coverage
SACOL1509	SACOL1509	-	57650424	115	64	0.35
SACOL1515	SACOL1515	-	57650430	412	186	0.52
SACOL1520	SACOL1520	-	57650434	148	75	0.23
SACOL1541	SACOL1541	-	57650454	110	32	0.71
SACOL1543 1	SACOL1543	-	57650456	130	76	0.3
SACOL1543 2	SACOL1543	-	57650456	231	157	0.46
SACOL1553	SACOL1553	-	57650465	213	103	0.53
SACOL1555	SACOL1555	-	57650467	269	161	0.32
SACOL1560	SACOL1560	-	57650472	349	184	0.47
SACOL1561	SACOL1561	-	57650473	169	81	0.27
SACOL1562 1	SACOL1562	-	57650474	317	200	0.57
SACOL1562 2	SACOL1562	-	57650474	396	290	0.31
SACOL1588	SACOL1588	-	57651924	347	167	0.45
SACOL1591	SACOL1591	-	57651927	108	-	0.55
SACOL1593	SACOL1593	-	57651929	303	194	0.24
SACOL1611	SACOL1611	-	57651947	137	86	0.72
SACOL1620	SACOL1620	-	57651956	443	238	0.67
SACOL1627	SACOL1627	-	57651963	62	21	0.44
SACOL1630	SACOL1630	-	57651966	105	54	0.23
SACOL1648	SACOL1648	-	57651984	109	35	0.73
SACOL1649	SACOL1649	-	57651985	269	205	0.37
SACOL1651	SACOL1651	-	57651987	48	24	0.23
SACOL1661	SACOL1661	-	57651997	141	41	0.47
SACOL1669	SACOL1669	-	57650496	344	248	0.41
SACOL1670	SACOL1670	-	57650497	78	66	0.1
SACOL1672	SACOL1672	-	57650499	96	-	0.84
SACOL1677	SACOL1677	-	57650504	238	116	0.37
SACOL1681	SACOL1681	-	57650508	139	35	0.68
SACOL1688	SACOL1688	-	57650514	105	16	0.49
SACOL1724	SACOL1724	-	57650549	343	263	0.55
SACOL1749	SACOL1749	-	57650572	358	273	0.43
SACOL1751	SACOL1751	-	57650574	172	76	0.35
SACOL1759 1	SACOL1759	-	57652008	371	209	0.87
SACOL1759 2	SACOL1759	-	57652008	199	97	0.63
SACOL1762 1	SACOL1762	-	57652011	272	170	0.52
SACOL1762 2	SACOL1762	-	57652011	272	170	0.52
SACOL1768	SACOL1768	-	57652017	143	92	0.29
SACOL1772 1	SACOL1772	-	57652021	283	171	0.43
SACOL1772 3	SACOL1772	-	57652021	435	270	0.52
SACOL1772 4	SACOL1772	-	57652021	134	57	0.32
SACOL1787 1	SACOL1787	-	57652035	66	1	0.32
SACOL1787 2	SACOL1787	-	57652035	366	162	0.55
SACOL1787 3	SACOL1787	-	57652035	330	185	0.44
SACOL1792	SACOL1792	-	57652040	238	104	0.5
SACOL1793	SACOL1793	-	57652041	166	31	0.5

Label[1]	Locus	Symbol	GI#	protein score[2]	total ion score[3]	sequence coverage
SACOL1794	SACOL1794	-	57652042	280	215	0.78
SACOL1801	SACOL1801	-	57652049	452	227	0.57
SACOL1891 1	SACOL1891	-	57650624	434	221	0.82
SACOL1891 2	SACOL1891	-	57650624	120	13	0.51
SACOL1894	SACOL1894	-	57650627	122	27	0.84
SACOL1895 1	SACOL1895	-	57650628	62	29	0.42
SACOL1895 2	SACOL1895	-	57650628	285	203	0.64
SACOL1902 1	SACOL1902	-	57650634	259	175	0.89
SACOL1902 2	SACOL1902	-	57650634	323	209	0.99
SACOL1912 1	SACOL1912	-	57650644	150	57	0.62
SACOL1912 2	SACOL1912	-	57650644	252	158	0.68
SACOL1912 3	SACOL1912	-	57650644	426	291	0.81
SACOL1919	SACOL1919	-	57650651	54	26	0.38
SACOL1920	SACOL1920	-	57650652	298	155	0.46
SACOL1931	SACOL1931	-	57652092	49	24	0.25
SACOL1933	SACOL1933	-	57652094	201	69	0.76
SACOL1936	SACOL1936	-	57652097	119	90	0.4
SACOL1952 1	SACOL1952	-	57652113	133	70	0.25
SACOL1952 2	SACOL1952	-	57652113	190	76	0.46
SACOL1952 3	SACOL1952	-	57652113	330	248	0.43
SACOL1952 4	SACOL1952	-	57652113	162	105	0.42
SACOL1968	SACOL1968	-	57650658	72	37	0.53
SACOL1975	SACOL1975	-	57650665	285	119	0.36
SACOL1985	SACOL1985	-	57650675	453	164	0.92
SACOL1992	SACOL1992	-	57650682	145	-	0.74
SACOL2000	SACOL2000	-	57650690	301	186	0.33
SACOL2020	SACOL2020	-	57650705	62	5	0.31
SACOL2028	SACOL2028	-	57650712	281	186	0.41
SACOL2035	SACOL2035	-	57650718	286	116	0.59
SACOL2038	SACOL2038	-	57650721	259	168	0.41
SACOL2044	SACOL2044	-	57650727	167	142	0.26
SACOL2053 1	SACOL2053	-	57650735	359	76	0.41
SACOL2053 2	SACOL2053	-	57650735	217	44	0.25
SACOL2074	SACOL2074	-	57650756	345	185	0.47
SACOL2106	SACOL2106	-	57652136	173	117	0.24
SACOL2114 1	SACOL2114	-	57652144	342	138	0.48
SACOL2114 2	SACOL2114	-	57652144	177	62	0.37
SACOL2125	SACOL2125	-	57652155	238	75	0.34
SACOL2131	SACOL2131	-	57652161	191	132	0.47
SACOL2131 1	SACOL2131	-	57652161	333	281	0.55
SACOL2131 2	SACOL2131	-	57652161	245	214	0.35
SACOL2133 1	SACOL2133	-	57652163	406	275	0.31
SACOL2133 2	SACOL2133	-	57652163	80	20	0.15
SACOL2136	SACOL2136	-	57652166	459	237	0.92
SACOL2148	SACOL2148	-	57652176	284	216	0.34

Label[1]	Locus	Symbol	GI#	protein score[2]	total ion score[3]	sequence coverage
SACOL2156	SACOL2156	-	57652183	281	149	0.41
SACOL2161	SACOL2161	-	57652188	341	189	0.51
SACOL2163	SACOL2163	-	57652190	66	15	0.74
SACOL2171	SACOL2171	-	57652198	92	34	0.19
SACOL2192	SACOL2192	-	57650788	119	78	0.32
SACOL2196	SACOL2196	-	57650792	298	171	0.59
SACOL2266	SACOL2266	-	57652206	457	288	0.51
SACOL2293	SACOL2293	-	57652233	314	88	0.61
SACOL2296	SACOL2296	-	57652236	388	120	0.62
SACOL2301	SACOL2301	-	57652241	245	107	0.3
SACOL2313	SACOL2313	-	57650862	253	177	0.49
SACOL2321 1	SACOL2321	-	57650870	522	383	0.44
SACOL2321 2	SACOL2321	-	57650870	228	166	0.39
SACOL2335	SACOL2335	-	57650884	80	32	0.27
SACOL2344	SACOL2344	-	57650892	152	48	0.76
SACOL2367	SACOL2367	-	57650914	132	-	0.46
SACOL2379 1	SACOL2379	-	57650925	305	189	0.57
SACOL2379 2	SACOL2379	-	57650925	238	162	0.62
SACOL2385	SACOL2385	-	57650931	138	75	0.42
SACOL2400	SACOL2400	-	57650945	34	23	0.2
SACOL2453	SACOL2453	-	57652263	245	79	0.32
SACOL2484	SACOL2484	-	57650984	265	165	0.72
SACOL2484	SACOL2484	-	57650984	65	25	
SACOL2488	SACOL2488	-	57650988	286	109	0.76
SACOL2499	SACOL2499	-	57650998	219	54	0.34
SACOL2501	SACOL2501	-	57651000	108	16	0.26
SACOL2518	SACOL2518	-	57651016	295	151	0.6
SACOL2519 1	SACOL2519	-	57651017	53	27	0.43
SACOL2519 2	SACOL2519	-	57651017	112	103	0.17
SACOL2532	SACOL2532	-	57651029	116	36	0.82
SACOL2535 1	SACOL2535	-	57651032	301	69	0.63
SACOL2535 2	SACOL2535	-	57651032	84	-	0.34
SACOL2563	SACOL2563	-	57651059	198	29	0.3
SACOL2574	SACOL2574	-	57652296	494	265	0.54
SACOL2575	SACOL2575	-	57652297	233	131	0.2
SACOL2596	SACOL2596	-	57652317	185	87	0.5
SACOL2597	SACOL2597	-	57652318	280	167	0.44
SACOL2609	SACOL2609	-	57652329	162	76	0.52
SACOL2616	SACOL2616	-	57652335	385	298	0.39
SACOL2624 1	SACOL2624	-	57652343	205	116	0.34
SACOL2624 2	SACOL2624	-	57652343	430	240	0.44
SACOL2650	SACOL2650	-	57652368	90	-	0.59
SACOL2667	SACOL2667	-	57652385	253	146	0.59
SACOL2708	SACOL2708	-	57651076	140	12	0.33
SACOL2710	SACOL2710	-	57651078	379	239	0.43

Label[1]	Locus	Symbol	GI#	protein score[2]	total ion score[3]	sequence coverage
SACOL2722	SACOL2722	-	57651090	226	150	0.33
SaeR	SACOL0766	SaeR	57651545	387	293	0.36
SdhA	SACOL1159	SdhA	57650258	244	125	0.29
SecA	SACOL0816	SecA	57650092	180	-	0.28
SerA 1	SACOL1773	SerA	57652022	416	178	0.43
SerA 2	SACOL1773	SerA	57652022	204	77	0.3
SerS	SACOL0009	SerS	57651117	300	232	0.3
SodA1	SACOL0118	SodA1	57652481	179	149	0.18
SodA2 2	SACOL1610	SodA2	57651946	188	160	0.21
SpoVG 1	SACOL0541	SpoVG	57650038	282	181	0.68
SpoVG 2	SACOL0541	SpoVG	57650038	114	66	0.62
SrrA	SACOL1535	SrrA	57650448	342	210	0.47
Ssb2	SACOL0438	Ssb2	57652626	513	254	0.84
SucA 1	SACOL1449	SucA	57651914	221	108	0.29
SucA 2	SACOL1449	SucA	57651914	98	58	0.13
SucB	SACOL1448	SucB	57651913	346	221	0.25
SufB 1	SACOL0918	SufB	57651608	182	89	0.18
SufB 2	SACOL0918	SufB	57651608	286	102	0.52
SufD 1	SACOL0915	SufD	57651605	331	158	0.54
SufD 2	SACOL0915	SufD	57651605	258	88	0.51
SufD 3	SACOL0915	SufD	57651605	352	217	0.44
SufD 4	SACOL0915	SufD	57651605	63	-	0.32
Sun	SACOL1229	Sun	57651786	265	155	0.25
Tal	SACOL1831	Tal	57652078	103	-	0.56
ThiD1	SACOL0626	ThiD1	57651455	143	94	0.38
ThrB	SACOL1364	ThrB	57650333	234	162	0.44
ThrC	SACOL1363	ThrC	57650332	380	220	0.4
ThrS 1	SACOL1729	ThrS	57650554	320	174	0.42
ThrS 2	SACOL1729	ThrS	57650554	378	263	0.29
ThrS 3	SACOL1729	ThrS	57650554	137	78	0.15
ThyA	SACOL1462	ThyA	57650383	106	34	0.26
Tig 1	SACOL1722	Tig	57650547	193	64	0.35
Tig 2	SACOL1722	Tig	57650547	344	211	0.4
Tkt 1	SACOL1377	Tkt	57650346	223	137	0.3
Tkt 2	SACOL1377	Tkt	57650346	257	133	0.32
TpiA 1	SACOL0840	TpiA	57650116	186	54	0.67
TpiA 2	SACOL0840	TpiA	57650116	427	248	0.78
TrmU	SACOL1676	TrmU	57650503	132	29	0.4
TrxA	SACOL1155	TrxA	57651754	210	90	0.99
TrxB 1	SACOL0829	TrxB	57650105	317	163	0.65
TrxB 2	SACOL0829	TrxB	57650105	161	41	0.52
Tsf 1	SACOL1276	Tsf	57651827	180	35	0.55
Tsf 2	SACOL1276	Tsf	57651827	132	25	0.42
Tuf 2	SACOL0594	Tuf	57651424	437	200	0.72
Tuf 3	SACOL0594	Tuf	57651424	537	206	0.85

Label[1]	Locus	Symbol	GI#	protein score[2]	total ion score[3]	sequence coverage
Tuf 4	SACOL0594	Tuf	57651424	111	74	0.22
Tuf 5	SACOL0594	Tuf	57651424	341	138	0.58
Tuf 7	SACOL0594	Tuf	57651424	138	54	0.21
Tuf 8	SACOL0594	Tuf	57651424	59	19	0.23
TypA	SACOL1118	TypA	57651718	295	147	0.32
TyrS	SACOL1778	TyrS	57652027	274	92	0.51
Udk	SACOL1666	Udk	57652002	263	99	0.85
Ung	SACOL0627	Ung	57651456	88	14	0.49
Upp 1	SACOL2104	Upp	57652134	242	67	0.91
UreE	SACOL2283	UreE	57652223	107	32	0.64
UvrA 1	SACOL0824	UvrA	57650100	121	42	0.24
UvrA 2	SACOL0824	UvrA	57650100	173	53	0.26
ValS 1	SACOL1710	ValS	57650536	110	56	0.21
ValS 2	SACOL1710	ValS	57650536	208	130	0.24
VraR	SACOL1942	VraR	57652103	362	163	0.93
YlmH	SACOL1204	YlmH	57651761	43	12	0.22
YycF	SACOL0019	YycF	57651127	197	78	0.49
Zwf 1	SACOL1549	Zwf	57650461	295	138	0.28
Zwf 2	SACOL1549	Zwf	57650461	152	59	0.16

[1] On the 2D master gel identified protein spots were labeled with the gene symbol, if available, or locus tag. Multiple Spots of the same proteins were numbered consecutively.

[2] provided by MASCOT

[3] if MS/MS are available
